# Aryl iodane-induced cascade arylation–1,2-silyl shift–heterocyclization of propargylsilanes under copper catalysis

**DOI:** 10.3762/bjoc.21.154

**Published:** 2025-09-26

**Authors:** Rasma Kroņkalne, Rūdolfs Beļaunieks, Armands Sebris, Anatoly Mishnev, Māris Turks

**Affiliations:** 1 Institute of Chemistry and Chemical Technology, Faculty of Natural Sciences and Technology, Riga Technical University, P. Valdena str. 3, LV-1048, Riga, Latvia; 2 Latvian Institute of Organic Synthesis, Aizkraukles str. 21, LV-1006, Riga, Latvia

**Keywords:** arylation reactions, copper-catalysis, iodanes, propargylsilanes, 1,2-silyl shift

## Abstract

A novel copper-catalyzed arylation strategy for propargylsilanes utilizing diaryl-λ^3^-iodanes has been developed, enabling a cascade sequence involving 1,2-silyl migration and heterocyclization. The β-silicon effect facilitates the formation of stabilized allyl cation intermediates that undergo regioselective trapping by internal *O*- and *N*-nucleophiles furnishing functionalized heterocycles. This method provides access to tetrahydrofuran or pyrrolidine frameworks, each bearing a trifunctionalized (*E*)-configured vinyl side chain. The use of a shorter linker provides entry to 1,2,3,6-tetrahydropyridines. Additionally, in the absence of internal nucleophiles, this methodology yields aryl-substituted 1,3-dienes. This work introduces a palladium-free, single-step alternative to multistep heterocycle construction from propargylsilanes and highlights the synthetic potential of iodane-mediated carbofunctionalization under copper catalysis.

## Introduction

**Scheme 1:**
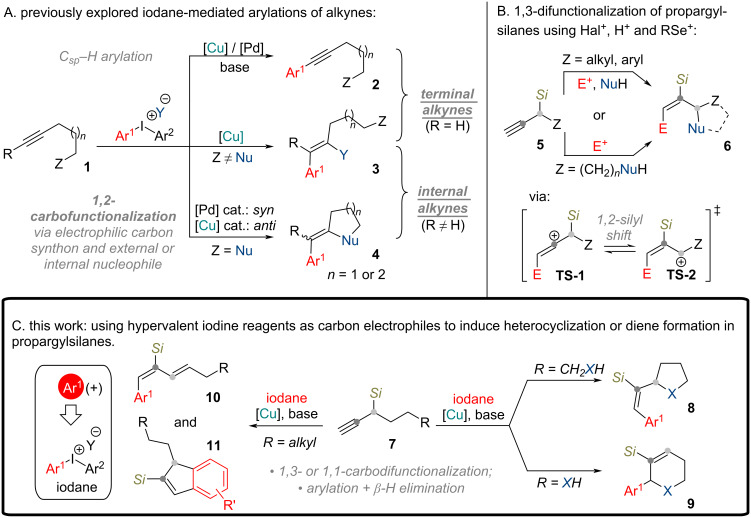
Alkyne arylation with diaryl-λ^3^-iodanes in the context of 1,2-silyl shift and potential cyclization.

Highly electrophilic hypervalent iodine(III) reagents are considered as arene electrophilic synthons, making them the reagents of choice for arylation reactions, where an umpolung of reactivity is required [1]. Arylations employing diaryl-λ^3^-iodanes can be performed under metal-free [2] or metal-catalyzed conditions. For alkyne arylations [Cu] [3] or [Pd] catalysis [4–6] is typically employed. Internal alkynes undergo 1,2-carbofunctionalization, where the highly electrophilic Ar–M species adds to the alkyne, generating a vinyl cation intermediate [7], which typically reacts with an internal nucleophile to form five- [8–9] or six-membered rings [7,9–10] (Scheme 1A). Thus far the internal nucleophilic species are limited to aryl- [7–8,10] or heteroaryl groups [7–8]. In one example methyl ethers were used [9]. Under [Pd]-catalyzed conditions a *syn*-type addition is observed [8,11], while [Cu] catalysts promote *anti*-addition [7,10]. In substrates prone to cationic rearrangements (or hydride shifts), more interesting reaction patterns can be observed. For example, branched aliphatic chain-containing alkynes are arylated and carbocyclized into cyclopentene derivatives [12–13].

The triflate moiety of the diaryl-λ^3^-iodane may act as an external nucleophile, resulting in 1,2-carbotriflation products [14]. Interestingly, this also works for terminal alkynes, which are typically known to undergo direct C(sp)–H arylation instead [15–16]. In the context of 1,2-carbofunctionalization, terminal alkynes are more scarcely studied. Among those few examples is a trifluoromethylative thiocyclization reaction [17] and a [4 + 2] annulation reaction between *o*-carboxylic ester-containing diaryl-λ^3^-iodanes and some terminal alkynes [18].

Looking to expand the possibilities for terminal alkyne carbofunctionalization, we turned our attention to propargylsilanes, which are prone to undergo cationic rearrangements via the 1,2-silyl shift, enabled by the β-cation-stabilizing properties of silyl groups [19–20]. This phenomenon has been successfully employed in 1,3-difunctionalization events in both intermolecular [21] and intramolecular fashion [22] (Scheme 1B). Thus far such propargylsilane rearrangements have been induced by addition of external halogen or selenium electrophiles and Brønsted acids. This encouraged us to develop a methodology involving a copper-catalyzed terminal alkyne arylation of propargylsilanes by diaryl-λ^3^-iodanes, followed by 1,2-silyl shift and terminated by nucleophile addition on intermediate allyl cation (Scheme 1C). The obtained tetrahydrofuran and pyrrolidine derivatives with highly substituted vinyl side-chains are regarded as privileged structures in medical chemistry [23–24]. Moreover, the resulting styryl functionality (Ph-C=C-) is often found in drug molecules as it improves lipophilicity, oral absorption and biological activity [25].

## Results and Discussion

### Arylation of aliphatic chain-containing propargylsilanes

**Scheme 2:**
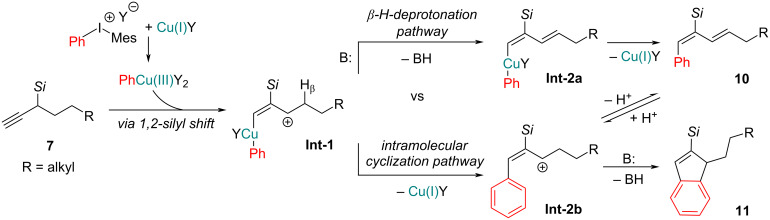
Competing mechanistic pathways for diene **10** and indene **11** formation.

We started our investigation with the arylation of aliphatic chain-containing propargylsilanes. The starting material – *tert*-butyl(hept-1-yn-3-yl)dimethylsilane (**7a**) – and analogous aliphatic chain-containing propargylsilanes **7b**,**c** were obtained from the commercial hept-1-yne [26]. While searching for appropriate arylation conditions (Table 1, entry 1) we observed the formation of both the arylated diene **10a** and silylindene **11a** (≈75:25). Both products are likely formed via the allylic cation intermediate **Int-1** (Scheme 2), from where on two competing mechanistic pathways are possible. Deprotonation of the β-H and reductive elimination affords diene **10**. Alternatively, an intramolecular cyclization leads to silylindenes **11**.

**Table 1:** Reaction conditions optimization for the arylation of aliphatic chain-containing propargylsilane **7a**.

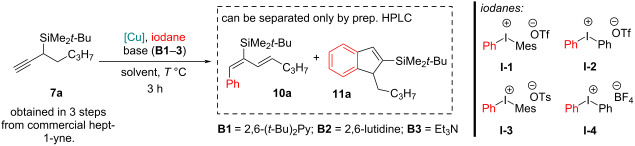								

Entry	Iodane (equiv)	[Cu] (mol %)	Base (equiv)	Solvent (abs.)^a^	*T* (°C)	Recovered **7a** (%)^b^	(1*E,*3*E*)-**10a**/**11a** (ratio)^c^	(1*E,*3*E*)-**10a**, (%)^b^

1^d^	**I-1** (1)	CuCl (20)	**B1** (1.3)	EtOAc	60	52	75:25	35

2^d^	**I-1** (1)	[CuOTf]_2_∙PhH (15)	**B1** (1.3)	EtOAc	60	42	68:32	32

3^d^	**I-1** (1)	Cu(OTf)_2_ (23)	**B1** (1.3)	EtOAc	60	63	60:40	12

4^d^	**I-1** (1)	CuCl (20)	**B1** (1.3)	Tol	60	78	77:33	17

5^d^	**I-1** (1)	CuCl (20)	**B1** (1.3)	DCE	60	53	72:28	34

6^d^	**I-1** (1)	CuCl (20)	**B1** (1.3)	MeNO_2_	60	69	–	0

7^d^	**I-1** (1)	CuCl (20)	**B2** (1.2)	EtOAc	60	95	–	0

8^d^	**I-1** (1)	CuCl (20)	**B3** (1.2)	EtOAc	60	61	–	2

9^d^	**I-1** (3)	CuCl (20)	**B1** (3.0)	EtOAc	60	0	60:40	60

10^d^	**I-1** (3)	CuCl (5)	**B1** (3.0)	EtOAc	60	0	67:33	35

11^e^	**I-2** (3)	CuCl (20)	**B1** (1.2)	EtOAc	60	0	84:16	38

12^d^	**I-3** (3)	CuCl (20)	**B1** (1.3)	EtOAc	60	97	–	0

13^d^	**I-4** (4)	CuCl (20)	**B1** (1.3)	EtOAc	60	98	–	0

14^d^	**I-1** (3)	Cu(MeCN)_4_BF_4_ (8)	**B1** (1.3)	EtOAc	60	0	63:37	60

15^e^	**I-1** (3)	CuCl (20)	**B1** (1.2)	EtOAc	70	0	71:29	59

16^e^	**I-2** (3)	CuCl (20)	**B1** (1.2)	EtOAc	70	0	85:15	**63**

17^f^	**I-2** (3)	CuCl (20)	**B1** (1.2)	EtOAc	70	0	87:13	**66**

18^e^	**I-2** (3)	CuCl (20)	**B1** (1.2)	EtOAc *c***_7a_** = 0.05 mmol/mL	70	0	**98:2**	35

^a^Starting material **7a** concentration in solvent was 0.1 mmol/mL unless stated otherwise. ^b^NMR yield. ^c^Molar ratio of products in the crude mixture, determined by ^1^H NMR in CDCl_3_. ^d^Reaction scale: 0.24 mmol of **7a**. ^e^Reaction scale: 0.48 mmol of **7a**. ^f^Reaction scale: 1.90 mmol of **7a**.

We were interested to see whether the reaction selectivity can be directed towards the formation of arylated dienes **10**, which would directly contribute to our previous work [26]. Therefore, we performed optimization of the arylation conditions (Table 1) to improve the reaction selectivity towards the aryldiene **10a** formation, indicated by the diene/indene **10a**/**11a** molar ratio (determined by NMR).

Among the tested diaryl-λ^3^-iodanes, only those containing a triflate anion showed reactivity. PhMesIOTf (**I-1**) and Ph_2_IOTf (**I-2**) showed comparable results in terms of diene yield, however, the symmetrical iodane **I-2** displayed better chemoselectivity towards diene formation (Table 1, entries 15 and 16).

In accordance with the proposed reaction mechanism (Scheme 2), an equimolar amount of protons is generated in the reaction, which would likely induce the formation of additional side-products either by protodecupration [27] or the acid-catalyzed propargylsilane activation pathway [26]. Therefore, the presence of a base was imperative. The only applicable base was found to be the non-nucleophilic 2,6-di-*tert-*butylpyridine (**B1**, Table 1, entries 1–6, 9–18). We also considered the structurally similar, but less sterically hindered 2,6-lutidine (**B2**, Table 1, entry 7) and Et_3_N (**B3**, Table 1, entry 8), both of which effectively halted the reaction. The same was observed when using *N*,*N*,*N*′,*N*′-tetramethylnaphthalene-1,8-diamine (“Proton sponge”) or TMEDA (see Supporting Information File 1 for details). In the case of Et_3_N (**B3**) and TMEDA, partial degradation of the starting material **7a** was also observed.

Among the tested solvents, EtOAc gave the best results (Table 1, entries 1–3, 7–18). As for reaction catalysts, Cu(I) salts demonstrated far better reactivity than Cu(II) salts, likely because the reaction proceeds via the Cu(I/III) catalytic cycle [28]. However, in the case of Cu(OTf)_2_, the active catalytic species must be generated in situ. The highest diene **10a** NMR yields (63–66%) were achieved by using 20 mol % of CuCl, 3.0 equiv of Ph_2_IOTf (**I-2**) and 1.2 equiv of 2,6-di-*tert-*butylpyridine (**B1**) in EtOAc at 70 °C (Table 1, entries 16 and 17). The obtained diene **10a** contained 13–15% of silylindene **11a** as impurity. Products **10a** and **11a** are separable by preparative HPLC. Lowering the starting material concentration in the reaction mixture to 0.05 mmol/mL significantly decreased the diene **10a** yield due to the formation of other unidentified impurities. However, it turned out to be the key to achieving the best diene/indene ratio **10a**/**11a =** 98:2. With the optimized conditions for aryldiene synthesis (Table 1, entries 16 and 17) we proceeded to explore the substrate scope (Table 2).

**Table 2:** Aryldiene scope.

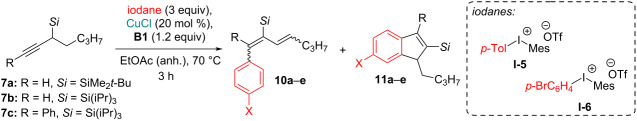						

Entry	Product	Iodane	**10**/**11**^a^ (mol/mol)	Yield **10** + **11** (%)	Isomeric ratio for diene **10** (mol/mol)^b^	

					(1*E*,3*E*)	∑(1*E*,3*Z*) + (1*Z*,3*E*) + (1*Z*,3*Z*)

1	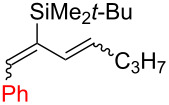 **10a**	**I-2**	87:13	51^c^	92	8
2	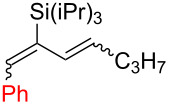 **10b**	**I-2**	67:33	61^d^	93	7
3	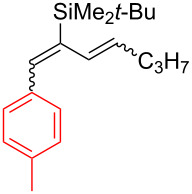 **10c**	**I-5**	85:15	69^d^	87	13
4	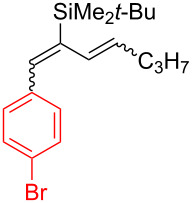 **10d**	**I-6**	98:2	37^d^	95	5
5	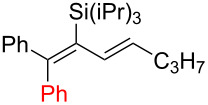 **10e**	**I-2**	–	0^e^	–	

^a^Ratio after column chromatography. ^b^Determined after C18 silica gel column chromatography by ^1^H NMR in CDCl_3_. ^c^Reaction scale: 1.90 mmol of **7**. ^d^Reaction scale: 0.48 mmol of **7**. ^e^No reaction at 70 °C in EtOAc (3 h) or 110 °C in BuOAc (17 h).

Arylations employing aryl groups with electron-donating substituents demonstrated higher yields (*p*-Tol > *p*-BrC_6_H_4_) (Table 2, entries 3 and 4). The *p*-bromo-substituted iodane **I-6**, albeit in a more modest yield, gave the diene **10d** with 98:2 selectivity. Switching to the more sterically challenged triisopropylsilyl (TIPS) group (Table 2, entry 2) resulted in higher indene proportion in the resulting mixture (**10b**/**11b** = 67:33). In all cases the (1*E*,3*E*)-dienes **10a**–**e** were obtained as the major isomer (87–95% isomeric purity). In small quantities the other isomers (1*Z*,3*E*) and (1*E*,3*Z*) or (1*Z*,3*Z*) were also detected by their characteristic signals in the ^1^H NMR spectra.

Interestingly, the internal alkyne **7c** (R = Ph, *Si* = TIPS) was completely unreactive under both standard arylation conditions (70 °C in EtOAc) and higher reaction temperatures (110 °C in BuOAc). In these cases, the starting material was recovered with no signs of degradation. This indicates that the provided method is limited to propargylsilanes containing a terminal alkyne group.

### Arylation of internal nucleophile-containing propargylsilanes

Internal nucleophile-containing substrates are interesting, because they offer the possibility of heterocyclization, as described in our previous work [22]. The internal *O*-nucleophile-containing starting material, 4-(*tert*-butyldimethylsilyl)hex-5-yn-1-ol (**7d**), was obtained from the commercial hex-5-yn-1-ol by a *O*-silylation and subsequent retro-Brook reaction sequence [29]. Arylation of silane **7d** with 1.2 equiv PhMesIOTf (**I-1**), 5 mol % CuCl and 1.2 equiv 2,6-(*t*-Bu)_2_Py in EtOAc gave the styryl side chain-containing tetrahydrofuran **8a** with 82% NMR yield after 3 h at 60 °C (Table 3, entry 2). The formed double-bond geometry in product **8a** was proved by the 2D-NOESY spectrum. The corresponding (*Z*)-isomer was not detected by NMR spectroscopy techniques, indicating that the observed arylation–cyclization cascade reaction is highly stereospecific. With this result in hand, we performed copper catalyst screening (Table 3).

**Table 3:** Conditions screening for copper-catalyzed arylation–cyclization of propargylsilane **7d**.

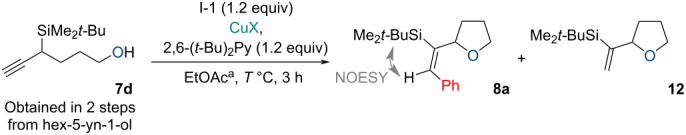				

Entry	CuX (mol %)	*T* (°C)	**8a**, % (NMR)	**12**, % (NMR)

1^b^	CuCl (2.5)	20	0	0
**2** **^c^**	**CuCl (5)**	**60**	**82, 76** **^d^**	**0**
3^b^	CuI (5)	60	0	0
**4** **^e,f^**	**[CuOTf]** **_2_** **∙PhH (5)**	**60**	**84**	**0**
5^e^	Cu(MeCN)_4_BF_4_ (5)	70	77	0
6^e^	Cu_2_O (5)	70	6	2
7^e^	[CuOTf]_2_∙PhH (2.5)	70	70	0
8^e,g^	[CuOTf]_2_∙PhH (2.5)	70	43	0
9^b^	[CuOTf]_2_∙PhH (11)	70	47	0

^a^*c***_7d_** = 0.10 mmol/mL, ^b^Reaction scale: 0.24 mmol of **7d**. ^c^Reaction scale: 4.2 mmol of **7d**. ^d^Isolated yield (%). ^e^Reaction scale: 0.47 mmol of **7d**. ^f^Reaction time: 2 h (full conv.). ^g^15 mol % of H_2_O as additive.

We observed that CuCl and the CuOTf benzene complex gave similarly good yields (82–84% by NMR, Table 3, entries 2 and 4), while the CuBF_4_ acetonitrile complex was the next best choice (77% by NMR, Table 3, entry 5). Heterogenous catalysis using Cu_2_O gave a mixture of the arylated product **8a** and protodecupration side-product **12**, both in poor yields (Table 3, entry 6). Increased catalyst loading (11 mol % of [CuOTf]_2_∙PhH) resulted in the formation of more side products (Table 3, entry 9), and we therefore decided to proceed with 5 mol % of the catalyst for the substrate scope.

We also observed that, in the absence of base, even at room temperature, only the protodecupration product **12** was obtained (with up to 69% NMR yield). A reaction temperature of 60 °C was found to be optimal and lower temperatures resulted in incomplete conversion.

**Scheme 3:**
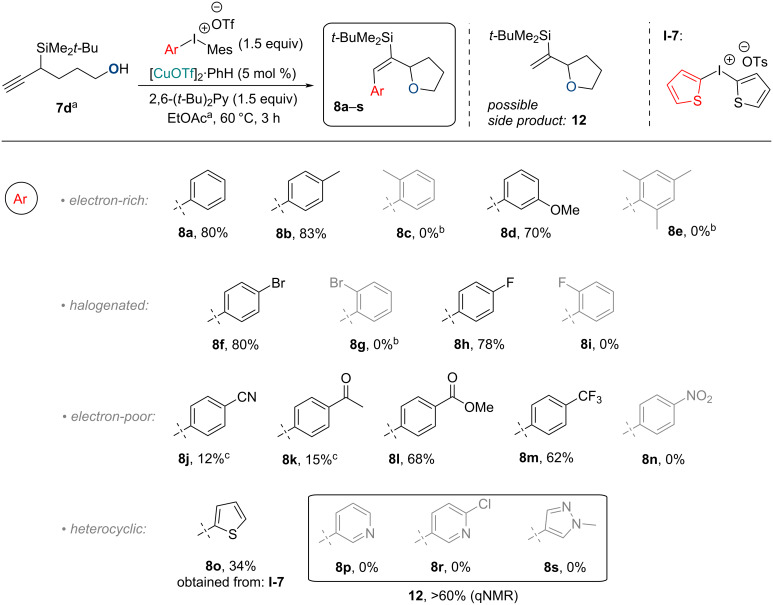
Reaction scope for the synthesis of arylated tetrahydrofurans **8**. Conditions: All reactions were performed on a 0.47 mmol scale. ^a^Starting material **7d** concentration: 0.1 mmol/mL. ^b^No reaction at 60 and 120 °C. ^c^Same result at 120 °C.

Under the optimized conditions we explored the reaction scope (Scheme 3). Unlike the previously explored dienes **10**, the obtained tetrahydrofuran products **8** were easy to purify by standard column chromatography techniques (30–50% DCM). Iodanes, containing electron-rich aryl groups (*p*-Tol, *m*-MeO-C_6_H_4_, Ph) or halogens (Br, F) gave the highest tetrahydrofuran **8** yields (70–83%). In contrast, electron-withdrawing group-containing iodanes, especially those holding nucleophilic heteroatoms (CN ≈ COR >> NO_2_) reacted poorly. Iodanes, containing electron-withdrawing groups with low nucleophilicity (CF_3_), however, gave fair yields. We also found that *ortho*-substituted iodanes did not provide arylation products, which can be attributed to the unfavorable steric hindrance [30].

Iodanes containing *N*-heterocycles, such as pyridine and pyrazole, provided the non-arylated tetrahydrofuran **12** as the main reaction product (Scheme 3), which is likely formed via the protodecupration pathway. However, to our delight the thiophenyl group was successfully added to the propargylsilane with 34% yield by using the symmetrical dithiophen-2-yliodonium *p*-tosylate (**I-7**). It should be noted that all products **8** were obtained with exclusive (*E*)-selectivity.

Tetrahydrofuran **8a** and some analogous compounds could be synthesized previously from alcohol **7d**, but in two steps, employing a halogen electrophile-induced propargylsilane **7d** halogenation–cyclization cascade and Suzuki–Miyaura cross-coupling in the second step [22]. Thus, we have developed a faster and palladium-free route towards tetrahydrofurans **8**. The latter can still be modified further through silicon–halide exchange followed by cross-coupling chemistry as described by us recently, among other possible transformations [21–22].

Interestingly, the addition of *O*-nucleophiles to form 1,3-carbofunctionalization products, can only be achieved in an intramolecular fashion. In the presence of an excess (5 equiv) of external an *O*-nucleophile R–OX (alcohol, carboxylic acid, its sodium salt or the silylated carboxylic acid), the arylation reaction of aliphatic chain-containing propargylsilane **7b** (standard conditions; see Scheme 3) only resulted in the arylation of the oxygen nucleophile itself.

**Scheme 4:**
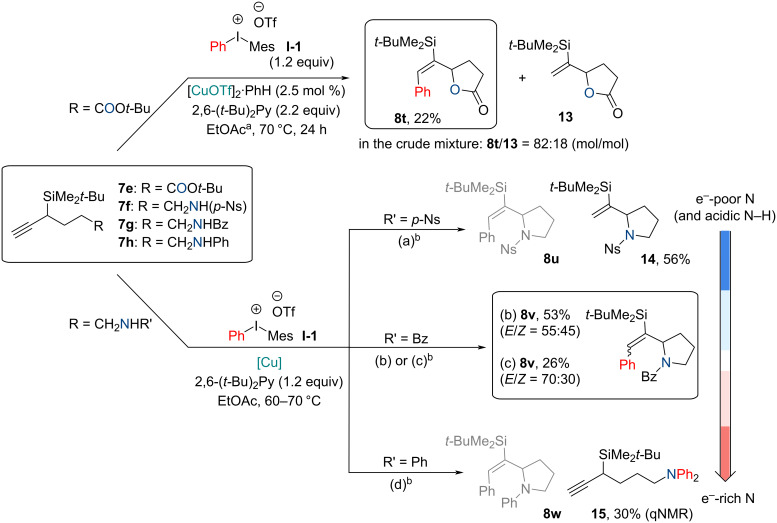
Synthesis of lactone and pyrrolidine derivatives. Conditions: ^a^*c***_7e_** = 0.1 mmol/mL. ^b^Reaction conditions modifications: a) 1.2 equiv **I-1**, CuCl (5 mol %), 60 °C, 3 h, *c***_7f_** = 0.3 mmol/mL; b) 3.0 equiv **I-1**, CuCl (10 mol %), 70 °C, 3 h, *c***_7g_** = 0.1 mmol/mL; c) 1.2 equiv **I-1**, [CuOTf]_2_·PhH (2.5 mol %), 70 °C, 22 h, *c***_7g_** = 0.1 mmol/mL; d) 1.2 equiv **I-1**, [CuOTf]_2_·PhH (2.5 mol %), 70 °C, 3 h, *c***_7h_** = 0.1 mmol/mL.

Next, we proceeded to expand the substrate scope by exploring other internal nucleophiles (Scheme 4), that could be used instead of the alcohol. The carboxylic acid-containing silane **7** (R = COOH), which was obtained by stepwise oxidation of the alcohol **7d**, failed to give the desired lactone **8t** product due to *O*-arylation of the carboxylic acid, leading to phenyl alkyl ester formation. Its silylated version (**7**, where R = COOSi(Me)_3_) only resulted in starting material degradation. Interestingly, *tert*-butyl ester **7e** (R = COO*t*-Bu) provided the desired arylated lactone **8t**, along with the protodecupration product **13**, which was formed in excess under the standard arylation conditions (Scheme 4). An additional equivalent of base (2.2 equiv of 2,6-(*t*-Bu)_2_Py) helped to suppress the formation of the non-arylated product **13**, making the arylated lactone **8t** the main reaction product. By adding a larger excess of base, conversion rates were significantly reduced. For detailed reaction optimization see Supporting Information File 1.

Next, we switched to internal *N*-nucleophiles. *N*-Nosylated starting material **7f** under standard arylation conditions gave the 2-(1-(*tert*-butyldimethylsilyl)vinyl)-1-((4-nitrophenyl)sulfonyl)pyrrolidine (**14**) with a 56% yield. Most likely in this case sulfonamide N–H is too acidic and this may lead to protodecupration. The arylation–cyclization of benzamide **7g** afforded the pyrrolidine derivative **8v** as a mixture of (*E*/*Z*)-isomers. By treating the isomeric mixture (*E*/*Z* = 55:45) with HNTf_2_ (0.9 equiv) in DCM (0.03 mmol/mL) and stirring at 0 °C to rt for 3 h, (Z)-**8v** selectively degraded and the pure product (*E*)-**8v** could be isolated with 47% yield. On the other hand, if the amine group was too nucleophilic, as in the case of *N*-(4-(*tert*-butyldimethylsilyl)hex-5-yn-1-yl)aniline (**7h**), the amine N–H was arylated instead of the alkyne, resulting in the formation of *N*-(4-(*tert*-butyldimethylsilyl)hex-5-yn-1-yl)-*N*-phenylaniline (**15**). Thus, acylamides were found to have optimal p*K*_a_ and nucleophilicity to react according to our desired pathway (Scheme 4).

**Scheme 5:**
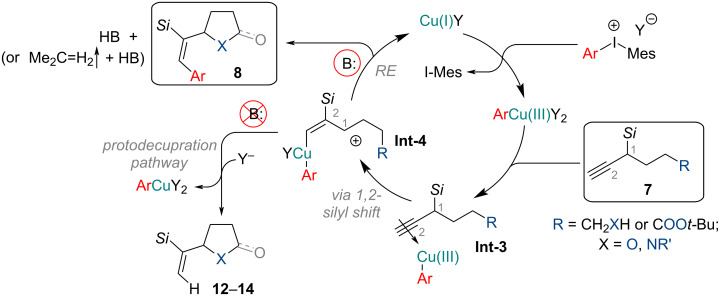
Proposed arylation–heterocyclization mechanism for internal nucleophile-containing silanes **7**.

For the heterocyclization mechanism under arylating conditions, we propose a Cu(I/III) catalyzed pathway (Scheme 5). Firstly, the copper(I) salt reacts with the diaryl-λ^3^-iodane (ArMesIY) to generate the strongly electrophilic arylcopper(III) species. The latter activates the propargylic system to induce the 1,2-silyl shift, producing the allylic cation intermediate **Int-4**, which is stabilized by the β-silicon effect. A following attack of an internal nucleophile (*O*-, *N*-) and reductive elimination affords the arylated product **8** and regenerates the Cu(I) catalyst. The added base (B:) traps the H^+^, generated in this catalytic cycle. In the case of *tert*-butyl esters (**7**: R = COO*t*-Bu) an equivalent of isobutylene gas is released as a side-product. In the absence of a base, the protodecupration pathway is predominant, affording non-arylated heterocycles **12**–**14** instead.

**Scheme 6:**
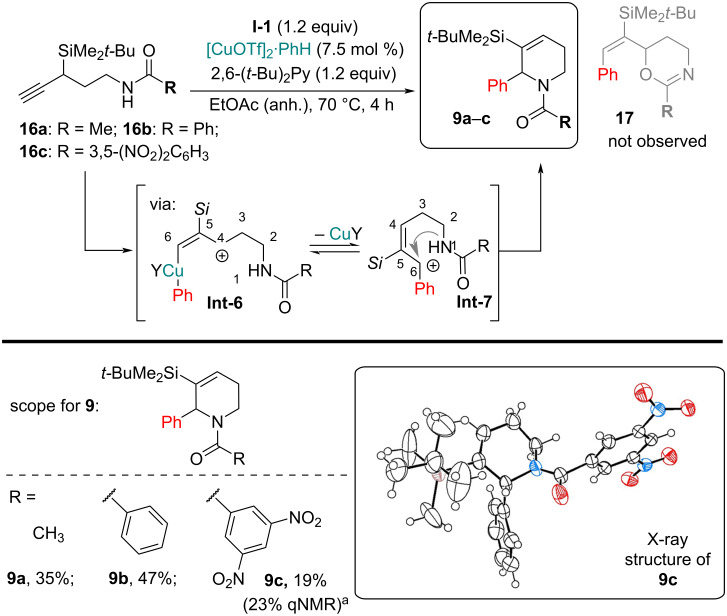
Arylation of C5-chain containing acylamides **16a**–**c**. ^a^The reaction was performed under modified conditions: **I-2** (3.0 equiv), 20 h because under standard reaction conditions incomplete conversion was observed (with 48% recovered **16c**).

When considering shorter carbon chain-containing acylamides **16a**–**c** as starting materials, we expected a similar cyclization pattern, involving imidate formation [10]. Instead aminopentynes **16a**–**c** underwent allylic rearrangement, affording tetrahydropyridines **9** (Scheme 6). The tetrahydropyridine core was confirmed unambiguously by X-ray structure analysis of dinitrobenzamide **9c**. More electron-rich acylamides reacted faster and gave less side-products, compared to more electron-poor analogs, that needed longer reaction times to reach full conversion (4 h for R = Ph, Me; 20 h for R = 3,5-(NO_2_)_2_C_6_H_3_).

## Conclusion

In this work we have reported copper-catalyzed arylation reactions of propargylsilanes, using iodanes as the electrophilic aryl synthon equivalents. For internal nucleophile-containing propargylsilanes subsequent heterocyclization was observed, and, depending on the chain length, giving either 1,3- or 1,1-carbofunctionalization products: tetrahydrofuran **8a**–**o** and pyrrolidine **8v** derivatives, containing a functionalized styryl group in the side chain, and 1,2,3,6-tetrahydropyridine derivatives **9a**–**c**. We observed a distinct preference for (*E*)-geometry formation in the resulting C=C bond. Internal alcohol, ester, and acylamide functional groups were found to be compatible with the reaction conditions, whereas for external nucleophiles *N*- or *O*-arylation precedes that of the alkyne. In the absence of reactive nucleophiles, aryl-substituted 1,3-dienes were obtained. The presented conditions were best applicable for arylations introducing aryl fragments with medium to high electron density, although some less electron-rich iodanes could be used, albeit in more modest yields. In contrast to previously studied alkyne 1,2-carbofunctionalization reactions [7–10], which were mostly limited to internal alkynes, only terminal alkyne-containing propargylsilanes were found to be reactive under these conditions. In summary, we have demonstrated that under copper-catalyzed conditions aryl iodanes can arylate propargylsilanes and induce a 1,2-silyl shift to generate β-Si-stabilized allyl cations. Compared to the previously employed two-step halocyclization–cross-coupling sequence [22], this approach offers a shorter, [Pd]-free synthetic sequence to the modified styryl-containing tetrahydrofuran **8a** and its previously unknown analogs. Efforts to broaden the scope of propargylsilanes by modifying their backbones, which connect the propargylic position with the internal nucleophile, are currently underway in our laboratory and will be reported elsewhere.

## Supporting Information

File 1 Experimental data, synthesis procedures, ^1^H and ^13^C NMR spectra, and X-ray data.

## Data Availability

All data that supports the findings of this study is available in the published article and/or the supporting information of this article.
