# Supplementary material for: Aryl iodane-induced cascade arylation–1,2-silyl shift–heterocyclization of propargylsilanes under copper catalysis
Source: Beilstein J Org Chem. 2025 Sep 26;21:1984–94. doi: 10.3762/bjoc.21.154 (PMC12477899; doi:10.3762/bjoc.21.154)
Supplement: File 1 — Experimental data, synthesis procedures, 1H and 13C NMR spectra, and X-ray data. [file Beilstein_J_Org_Chem-21-1984-s001.pdf]

BEILSTEIN JOURNAL OF ORGANIC CHEMISTRY

Supporting Information

for

Aryl iodane-induced cascade arylation–1,2-silyl shift–heterocyclization of propargylsilanes under copper catalysis

Rasma Kroņkalne, Rūdolfs Beļaunieks, Armands Sebris, Anatoly Mishnev and Māris Turks

Beilstein J. Org. Chem. **2025,** *21,* 1984–1994. doi:10.3762/bjoc.21.154

Experimental data, synthesis procedures, ^{1}H and ^{13}C NMR spectra, and X-ray data

License and Terms: This is a supporting information file under the terms of the Creative Commons Attribution License (https://creativecommons.org/licenses/by/4.0). Please note that the reuse, redistribution and reproduction in particular requires that the author(s) and source are credited and that individual graphics may be subject to special legal provisions.
The license is subject to the *Beilstein Journal of Organic Chemistry* terms and conditions: (https://www.beilstein-journals.org/bjoc/terms)

Table of contents

General information S2

Optimization tables S3

Synthesis procedures/characterization data for starting materials.. S8

Synthesis procedure for (5-azidopent-1-yn-3-yl)(*tert*-butyl)dimethylsilane (**S3**) S8

General procedure A for the synthesis of C5-chain-containing acyl amides **16a**,**b** from azide S3 by using acid anhydrides as acylating reagents.. S9

Synthesis of *N*-(3-(*tert*-butyldimethylsilyl)pent-4-yn-1-yl)-3,5-dinitrobenzamide (**16c**) S11

Three-step procedure for the synthesis of *tert*-butyl 4-(*tert*-butyldimethylsilyl)hex-5-ynoate (**7e**) S12

Two step procedure for the synthesis of *N*-(4-(*tert*-butyldimethylsilyl)hex-5-yn-1-yl)aniline (**7h**).. S13

Synthesis procedures/characterization data for arylation products S15

General procedure B for the synthesis of silyl dienes S15

General procedure C for the synthesis of tetrahydrofuran derivatives......................... S21

Synthesis of (*E*)-5-(1-(*tert*-butyldimethylsilyl)-2-phenylvinyl)dihydrofuran-2(3*H*)-one (**8t**) S26

Synthesis of (2-(1-(*tert*-butyldimethylsilyl)-2-phenylvinyl)pyrrolidin-1-yl)(phenyl)methanone (**8u**) S27

Cyclization reaction of *N*-(4-(*tert*-butyldimethylsilyl)hex-5-yn-1-yl)-4-nitrobenzenesulfonamide (**7f**) S28

Synthesis of 6-phenyl-1,2,3,6-tetrahydropyridine **9a**–**c** derivatives............................. S29

^{1}H and ^{13}C NMR spectra.. S33

X-ray data.. S72

Crystal parameters and refinement metrics of compound **9c**....................................... S73

References S75

General information

The solvents used in the reactions were dried with standard drying agents and freshly distilled prior to use. Commercially available reagents were used as received. Compounds **S1**, **7a–d** and **7f** were prepared according to the procedures described in the literature [1-3].

All reactions were followed by TLC on E. Merck Kieselgel 60 F254, with detection by UV Full Paper light or developed using generic $KMnO_4$ or I_2 stain; GC analysis or NMR analysis.

Direct phase column chromatography was performed on silica gel (60 Å, 40–63 µm, ROCC). Reversed-phase column chromatography was performed on C18 silica gel (25–40 µm, LiChroprep RP-18). Preparative HPLC was performed using an Agilent Technologies 1200 Series system equipped with Eclipse XDB-C18 column, 9.4 × 250 mm, particle size 5 µm. Preparative TLC was done using Merck silica gel 60 F_{254} glass plates (20 × 20 cm).

^{1}H and ^{13}C{1H} NMR spectra were recorded with a Bruker Avance Neo 500 MHz spectrometer in $CDCl_3$, DCM-d_2, DMSO-d_6 or CD_3CN. Chemical shifts (δ) are reported in ppm and coupling constants (*J*) in Hz. Residual solvent (^{1}H) or solvent (^{13}C) peaks were used as internal reference ($CDCl_3$: δ = 7.26 ppm for ^{1}H NMR, δ = 77.16 ppm for ^{13}C{^{1}H} NMR; DCM-d_2: δ = 5.32 ppm for ^{1}H NMR, δ = 53.84 for ^{13}C{^{1}H} NMR; DMSO-d_6: δ =2.50 ppm for ^{1}H NMR, δ = 39.52 ppm for ^{13}C{^{1}H} NMR; CD_3CN: δ = 1.94 ppm for ^{1}H NMR, δ = 1.32 or 118.26 ppm for ^{13}C{^{1}H} NMR). Multiplicities are indicated as follows: s (singlet), d (doublet), t (triplet), q (quartet), sextet (sext), hept (heptet), m (multiplet), br (broad). The structural assignments were made with additional information from gCOSY, gHSQC, and gHMBC experiments.

Crystallographic diffraction data were collected with a NoniusKappa CCD diffractometer (Mo Kα, λ = 0.71073 Å) equipped with a low-temperature Oxford Cryosystems Cryostream Plus device.

GC analyses were performed using a Hewlett-Packard Agilent Technologies 6890 gas chromatograph with mass selective detector, equipped with capillary column Agilent Technologies DB-1MS (30 m × 0.32 mm × 0.25 µm). Injection temperature: 250 °C; splitless and injection volume 1 µL or split 1:300 and injection volume 0.2 µL; gas type: helium; flow rate: 1.2 mL/min; detector temperature: 230 °C; MS detector (EI, 70 eV).

High resolution mass spectra (ESI) were recorded with an Agilent 1290 Infinity series UPLC connected to an Agilent 6230 TOF mass spectrometer (calibration at *m*/*z* = 121.050873 and *m*/*z* = 922.009798), (ESI′) Thermo Fisher Scientific Orbitrap Exploris 120 mass spectrometer operating in full scan mode at the 120000 resolutions, (ESI′′) Q-TOF Micromass and elemental analyses on a Carlo-Erba EA1108 analyzer or (APCI) on 7T solariX XR (Bruker Daltonik GmbH) fourier-transform ion cyclotron resonance mass spectrometer (FT-ICR-HRMS) equipped with an APCI source. The source parameters were as follows: polarity – positive; corona needle – 4000 nA; capillary voltage – 3000 V; APCI head and transfer line – 295 °C. The mass spectrometer parameters were as follows: mode – absorption; size (transient length), 512k (0.245 s); accumulation time – 100 ms; scanning range – 100 – 1000 *m*/*z*. 1 µL of sample (in DCM) was introduced into the source using DB-5MS capillary column (15 m × 0.25 mm × 0.25 µm) using a generic temperature gradient program (80 to 320 °C). Mass accuracy during measurements was maintained using polysiloxane ($[((CH_3)_2SiO)_5 + H]^+$, 371.101233 *m*/*z*) as the lock mass.

Optimization tables

Table S1: Full optimization table for the synthesis of *tert*-butyldimethyl((1*E*,3*E*)-1-phenylhepta-1,3-dien-2-yl)silane (10a)

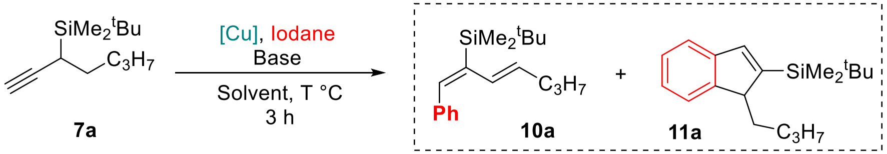

Can be separated only by prepHPLC

Iodanes:

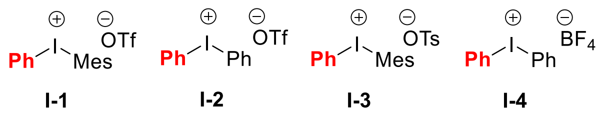

Entry	Iodane (equiv)	[Cu] (mol %)	Base (equiv)	Solvent (abs.)[a]	Additive	*T* °C	Recovered 7a, %[e]	(1*E*,3*E*)-10a/11a ratio[f]	(1*E*,3*E*)-10a, %[e]
1[b]	**I-1** (1)	CuCl (20)	2,6-*t*-Bu_2Py (1.2–1.3)	EtOAc	-	60	**52**	75:25	35
2[b]		$[CuOTf]_2$·PhH (15)					42	68:32	32
3[b]		$Cu(OTf)_2$ (23)					63	60:40	12
4[b]		CuI (22)					82	-	0
5[b]		CuCN (23)					71	-	0
6[b]		Cu (I) thiophene-2-carboxylate (22)					100	-	0
7[b]		CuCl (20)		Tol			78	77:33	17
8[b]				DCE			53	72:28	34
9[b]				$MeNO_2$			69	-	0
10[b]				DMF			54	-	0
11[b]				MeCN			91	-	0
12[b]				1,4-dioxane			76	71:29	10
13[b]				MEK			100	-	0
14[b,g]				PhCl			0	60:40	15
15[b,g]			-	Py			0	61:39	20

Table S1 continued

Entry	Iodane (equiv)	[Cu] (mol %)	Base (equiv)	Solvent (abs.)[a]	Additive	*T* °C	Recovered 7a, %[e]	(1*E*,3*E*)-10a/11a ratio[f]	(1*E*,3*E*)-10a, %[e]
16[b]	**I-1** (1)	CuCl (20)	2,6-*t*-Bu_2Py (1.2–1.3)	EtOAc	Cs_2CO_3 (1.2 equiv)	60	94	-	0
17[b]					NaOAc (4 equiv)		44	72:28	36
18[b]					PPh_3 (0.48)		91	-	0
19[b,h]					PPh_3 (0.24)	90	0	61:39	40
20[b,g]					XPhos (0.14)		0	66:34	43
21[b]			1,6-lutidine (1.2)		-	60	95	-	0
22[b]			Et_3N (1.2)				61	-	2
23[b]			TMEDA (1.2)				69	100:0	3
24[b]			Proton sponge (1.2)				98	-	0
25[b]	**I-1** (3)		DBU (1.4)				0	-[i]	10
26[b]			2,6-*t*-Bu_2Py (3.0)				0	60:40	60
27[b]		CuCl (5)					0	67:33	35
28[c]	**I-2** (3)	CuCl (20)	2,6-*t*-Bu_2Py (1.2–1.3)				0	84:16	38
29[b]	**I-3** (3)						97	-	0
30[b]	**I-4** (4)						98	-	0
31[b]	**I-1** (3)	Cu (33) + $Cu(OTf)_2$ (1)					0	68:32	13
32[b]		$Cu(MeCN)_4BF_4$ (8)					0	63:37	60
33[c]		**CuCl (20)**				70	0	71:29	59

Table S1 continued

Entry	Iodane (equiv)	[Cu] (mol %)	Base (equiv)	Solvent (abs.)[a]	Additive	*T* °C	Recovered 7a, %[e]	(1*E*,3*E*)-10a/11a ratio[f]	(1*E*,3*E*)-10a, %[e]
34[c]	**I-2** (3)	**CuCl (20)**	2,6-*t*-Bu_2Py (1.2-1.3)	EtOAc	-	70	0	85:15	**63**
35[d]							0	87:13	**66**
18[c]				EtOAc c_{7a}=0.05 mmol/ml	-		0	**98:2**	35

[a] – Starting material **7a** concentration in solvent was 0.1 mmol/ml unless stated otherwise; [b] – Reaction scale: 0.24 mmol of **7a**; [c] – Reaction scale: 0.48 mmol of **7a**; [d] – reaction scale: 1.90 mmol of **7a**; [e] – NMR yield; [f] – molar ratio of products in the crude mixture, determined by ^{1}H NMR in $CDCl_3$; [g] – 3.0–3.5 equiv of iodane; [h] – 2.5 equiv of iodane; [i] – indene could not be detected due to ^{1}H NMR signal overlap.

Table S2: Conditions screening for copper-catalyzed arylation-cyclization of propargyl silane 7d.

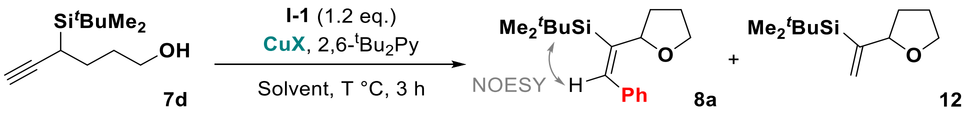

Entry	CuX	Solvent	T, °C	2,6-*t*-Bu_2Py (equiv)	Additives	8a, % (NMR)	12, % (NMR)
1[c]	CuCl (2.5 mol %)	EtOAc c_{7d}=0.10 mmol/mL	20	-	-	0	69
2[c]				1.2	-	0	0
3[a]	CuCl (5 mol %)		**60**		-	82, 76[e]	**0**
4[a,d]		THF c_{7d}=0.04 mmol/mL			-	25	
5[c]	CuI (5 mol %)	EtOAc c_{7d}=0.10 mmol/mL			-	0	0
**6[b,d]**	**$[CuOTf]_2$·PhH (5 mol %)**				-	**84**	**0**
7[b]	$Cu(MeCN)_4BF_4$ (5 mol %)		70		-	77	0
8[b]	Cu_2O (5 mol %)				-	6	2
9[b]	$[CuOTf]_2$·PhH (2.5 mol %)				-	70	0
10[b]					¼ drop of H_2O	43	0
11[c]	$[CuOTf]_2$·PhH (11 mol %)				-	47	0

[a] – Reaction scale: 4.2 mmol of **7d**; [b] – reaction scale: 0.47 mmol of **7d**; [c] – reaction scale: 0.24 mmol of **7d**; [d] – reaction time – 2 h (full conv.), [e] – isolated yield (%).

Table S3: Reaction optimization for the synthesis of (*E*)-5-(1-(*tert*-butyldimethylsilyl)-2-phenylvinyl)dihydrofuran-2(3*H*)-one (8t)

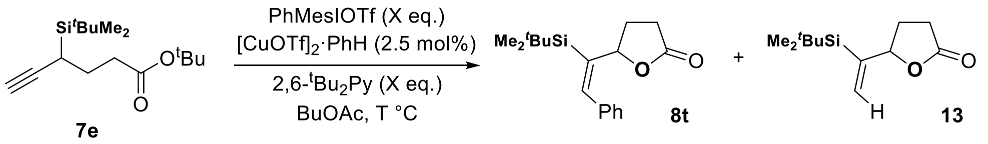

Entry	PhMesIOTf (mol%)	2,6-*t*-Bu$_2$Py (equiv)	c$_M$ of 7e in ROAc (mmol/mL)	*T* (°C)	*t* (h)	Result (qNMR) (%)			Ratio 8t:13 (n/n) by NMR[c]
						7e	8t	13	
1	1.2	1.2	0.035		14	0	15	47	24:76
2	1.2	1.2	0.095	100	3	0	16	66	22:78
3	3	2.2	0.095		20	0	32	21	65:35
**4**	**1.2**	**2.2**	**0.095**[a]	**70**	**24**[b]	**0**	**28**	**15**	**82:18**
5	1.1	2.1	0.033	100	6	0	22	42	32:68
6	1.2	10.0	0.021		6	48	15	20	43:57

[a] - EtOAc; [b] - After 6 h **7e** was still observed on GC; [c] - Molar ratio was determined in the crude mixture.

Synthesis procedure for (5-azidopent-1-yn-3-yl)(*tert*-butyl)dimethylsilane (S3)

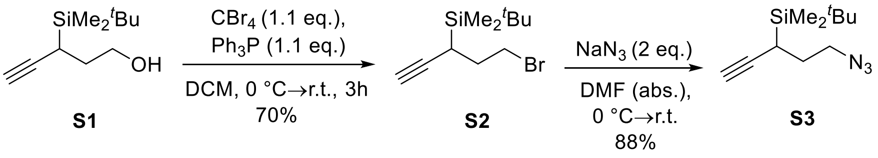

A 50 mL round-bottomed flask was charged with 3-(*tert*-butyldimethylsilyl)pent-4-yn-1-ol [1] (**S1**, 3.0 g, 15.1 mmol, 1.0 equiv) and DCM (30 mL). CBr_4 (5.5 g, 16.6 mmol, 1.1 equiv) was added to the solution while stirring. Next, the solution was cooled to 0 °C in an ice bath. Slowly and in small portions Ph_3P (4.4 g, 16.6 mmol, 1.1 equiv) was added to the cooled solution. The reaction was further stirred for 3 hours at room temperature. The solution was concentrated *in vacuo* and the concentrate was purified by column chromatography on silica (5–10% DCM/Hex). The bromide **S2** was obtained as a yellow oil (4.4 g, 63% purity by NMR, η = 70%) and used in the next step without additional purification. A small sample of the technical bromide **S2** was purified using preparative TLC (hexanes) to obtain the pure product **S2** as a clear oil.

A 25 mL round-bottomed flask (dried at 120 °C, degassed with argon), equipped with a Teflon-coated magnetic stirrer, was charged with NaN_3 (1.0 g, 15.3 mmol, 2.0 equiv), closed with a rubber septum and evacuated and backfilled with argon using the Schlenk line (3×). Dry DMF (9 mL) was added via syringe and the solution was cooled to 0 °C in an ice bath. Bromide **S2** (2.0 g, 7.7 mmol, 1.0 equiv) was added to the cooled solution via syringe. After stirring for 20 h, the solution was diluted with H_2O (20 mL) and transferred to a separatory funnel, followed by extraction with toluene (2 × 20 mL). The combined organic layers were concentrated *in vacuo*. The concentrate was dissolved in a mixture of DCM/Hex (1:10, 15 mL) and filtered through a silica plug (rinsed with 200 mL of 20% DCM/Hex). The filtrate was concentrated *in vacuo* to afford the azide **S3** as a yellow oil (2.2 g, 69% purity by NMR, η =88%). The azide **S3** could be further purified by column chromatography on silica (0–10% DCM/Hex) to afford a clear oil or used as is in the next step.

(5-Bromopent-1-yn-3-yl)(*tert*-butyl)dimethylsilane (**S2**):

Characterization data:

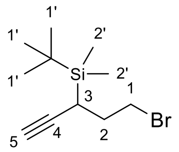

^{1}H NMR (500 MHz, $CDCl_3$): δ 3.71 (ddd, $^2J_{H\text{-}H}$ = 9.7 Hz, $^3J_{H\text{-}H}$ = 6.2, 4.8 Hz, 1H, $H_aC(1)$), 3.57 (ddd, $^2J_{H\text{-}H}$ = 9.7 Hz, $^3J_{H\text{-}H}$ = 8.3, 7.1 Hz, 1H, $H_bC(1)$), 2.06 – 1.89 (m, 4H, $H_2C(2)$, HC(3) and HC(5)), 0.97 (s, 9H, $H_3C(1')$), 0.10 (s, 3H, $H_3C(2')$), 0.04 (s, 3H, $H_3C(2')$).

^{13}C NMR (126 MHz, $CDCl_3$): δ 85.3, 70.2, 33.7, 33.3, 27.2, 17.8, 16.0, -7.1, -7.2.

HRMS (ESI'): $C_{11}H_{22}{}^{79}BrSi$ $[M+H]^+$ calc. 261.0674, found 261.0773.

$C_5H_6{}^{81}Br$ $[M\text{-}SiMe_2{}^tBu]^-$ calc. 146.9627, found 146.9661.

(5-Azidopent-1-yn-3-yl)(*tert*-butyl)dimethylsilane (**S3**):

Characterization data:

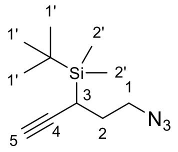

^{1}H NMR (500 MHz, $CDCl_3$): δ 3.58 (ddd, $^2J_{H\text{-}H}$ = 12.1 Hz, $^3J_{H\text{-}H}$ = 7.3, 4.1 Hz, 1H, $H_aC(1)$), 3.47 (ddd, $^2J_{H\text{-}H}$ = 12.1 Hz, $^3J_{H\text{-}H}$ = 8.9, 6.8 Hz, 1H $H_bC(1)$), 2.02 (d, $^4J_{H\text{-}H}$ = 2.8 Hz, 1H, HC(5)), 1.89 (ddd, $^3J_{H\text{-}H}$ = 12.2, 3.5 Hz, $^4J_{H\text{-}H}$ = 2.8 Hz, 1H, HC(3)), 1.74 (dddd, $^2J_{H\text{-}H}$ = 13.5 Hz, $^3J_{H\text{-}H}$ = 8.9, 7.3, 3.5 Hz, 1H, $H_aC(2)$), 1.66 (dddd, $^2J_{H\text{-}H}$ = 13.5 Hz, $^3J_{H\text{-}H}$ = 12.2, 6.8, 4.1 Hz, 1H, $H_bC(2)$), 0.7 (s, 9H, $H_3C(1')$), 0.10 (s, 3H, $H_3C(2')$), 0.04 (s, 3H, $H_3C(2')$).

^{13}C NMR (126 MHz, $CDCl_3$): δ 88.6, 70.3, 50.9, 29.4, 27.2, 17.7, 14.3, -7.1, -7.3.

HRMS: $C_{11}H_{22}N_3Si$ $[M+H]^+$ calc. 224.1583, found 224.1576.

General procedure A for the synthesis of C5-chain-containing acyl amides 16a,b from azide S3 by using acid anhydrides as acylating reagents

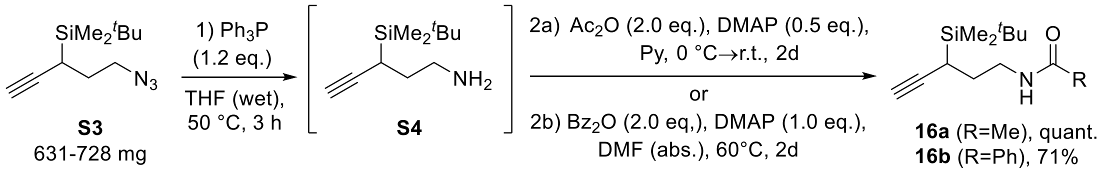

In a 50 mL round-bottomed flask, equipped with a Teflon-coated magnetic stirrer, (5-azidopent-1-yn-3-yl)(*tert*-butyl)dimethylsilane (**S3**, 2.82–3.26 mmol, 1.0 equiv) was dissolved in THF (wet; 20 mL). Ph_3P (1.2 equiv) was added portion-wise to the stirred solution at ambient temperature. A Snyder column was installed on the flask neck, and the reaction mixture was heated in an oil bath (50 °C) for 3 hours. The reaction mixture was concentrated in vacuo. DMAP (0.5–1.0 equiv) was added to the obtained amine/phosphines mixture. The flask was then sealed with a rubber septum, evacuated and filled with argon using the Schlenk line (3×). Pyridine or DMF (dry; 10 mL) was added via syringe. Ac_2O or Bz_2O (2.0 equiv) was added to the solution, and the reaction was stirred for 2 days. *(For the specific reaction temperatures and work-up procedures see each acyl amide separately.)* After work-up the crude mixture was purified using normal-phase column chromatography (30–60% EtOAc/Hex or 0–10% EtOAc/DCM).

N-(3-(*tert*-Butyldimethylsilyl)pent-4-yn-1-yl)acetamide (**16a**):

Synthesis procedure:

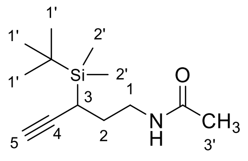

The reaction was performed following **General procedure A**: (5-azidopent-1-yn-3-yl)(*tert*-butyl)dimethylsilane (**S3**, 728 mg, 3.26 mmol, 1.0 equiv), Ph_3P (1026 mg, 3.91 mmol, 1.2 equiv), THF (wet; 20 mL), DMAP (199 mg, 1.63 mmol, 0.5 equiv), pyridine (10 mL), Ac_2O (0.61 mL, 6.52 mmol, 2.0 equiv). The addition of Ac_2O was done at 0 °C (ice bath), after which the reaction mixture was stirred at ambient temperature for 2 days.

For work-up the reaction mixture was transferred to a separatory funnel using EtOAc (30 mL), the organic phase was washed with 0.1 M HCl solution (3 × 40 mL) and saturated aqueous NaCl solution, dried over anhydrous Na_2SO_4, filtered, and the filtrate was concentrated in vacuo. Column chromatography (30–60% EtOAc/Hex) afforded the product **16a** as a yellow oil (725 mg, η = quant.).

Characterization data:

^{1}H NMR (500 MHz, $CDCl_3$) δ 5.79 (br s, 1H, H-N), 3.51 (dq, $^2J_{H\text{-}H}$ = 12.9 Hz, $^3J_{H\text{-}H}$ = 5.9 Hz, 1H, $H_aC(1)$), 3.39 (dq, $^2J_{H\text{-}H}$ = 12.9 Hz, $^3J_{H\text{-}H}$ = 6.7 Hz, 1H, $H_bC(1)$), 2.04 (d, $^4J_{H\text{-}H}$ = 2.6 Hz, 1H, HC(5)), 1.98 (s, 3H, $H_3C(3')$), 1.83 – 1.72 (m, 2H, $H_aC(2)$ and HC(3)), 1.55 (dt, $^2J_{H\text{-}H}$ = 12.7 Hz, $^3J_{H\text{-}H}$ = 6.7 Hz, 1H, $H_bC(2)$), 0.95 (s, 9H, $H_3C(1')$), 0.08 (s, 3H, $H_3C(2')$), 0.02 (s, 3H, $H_3C(2')$).

^{13}C NMR (126 MHz, $CDCl_3$) δ 170.2, 86.6, 70.3, 39.9, 29.4, 27.3, 23.6, 17.8, 14.7, -7.1, -7.3.

HRMS (ESI'): $C_{13}H_{26}NOSi$ $[M+H]^+$ calc. 240.1784, found 240.1777.

N-(3-(*tert*-Butyldimethylsilyl)pent-4-yn-1-yl)benzamide (**16b**):

Synthesis procedure:

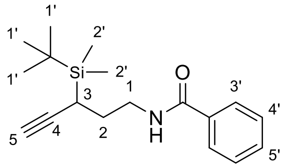

The reaction was performed following **General procedure A**: (5-azidopent-1-yn-3-yl)(*tert*-butyl)dimethylsilane (**S3**, 631 mg, 2.82 mmol, 1.0 equiv), Ph_3P (767 mg, 3.39 mmol, 1.2 equiv), THF (wet; 20 mL), DMAP (345 mg, 2.82 mmol, 1.0 equiv), DMF (dry; 10 mL), Bz_2O (1.278 g, 5.65 mmol, 2.0 equiv). The addition of Bz_2O was done at ambient temperature, after which the reaction mixture was heated in an oil bath (60 °C) for 2 days.

For work-up the resulting mixture was transferred to a separatory funnel using a mixture of toluene/EtOAc (40 mL; 4:1). The organic layer was washed with H_2O (3 × 40 mL), saturated aqueous NH_4Cl solution (30 mL) and saturated aqueous NaCl solution (30 mL), dried over anhydrous Na_2SO_4, filtered, and the filtrate was concentrated in vacuo. After column chromatography (0–10% EtOAc/DCM) the product **16b** contained significant quantities of benzoic acid, which was removed by dissolving the impure product in EtOAc (20 mL) and washing the organic phase with saturated aqueous $NaHCO_3$ solution (3 × 20 mL) and saturated aqueous NaCl solution (20 mL), followed by drying over anhydrous Na_2SO_4, filtration, and filtrate concentration in vacuo. The product **16b** was obtained as a yellow oil (605 mg, η = 71%).

Characterization data:

^{1}H NMR (500 MHz, $CDCl_3$) δ 7.78 (d, $^{3}J_{H\text{-}H}$ = 7.4 Hz, 2H, H-C(3')), 7.49 (t, $^{3}J_{H\text{-}H}$ = 7.4 Hz, 1H, H-C(5')), 7.43 (t, $^{3}J_{H\text{-}H}$ = 7.4 Hz, 2H, H-C(4')), 6.60 (br s, 1H, H-N), 3.74 – 3.59 (m, 2H, H_2C(1)), 2.10 (d, $^{4}J_{H\text{-}H}$ = 2.7 Hz, 1H, HC(5)), 1.97 – 1.79 (m, 2H, H_aC(2) and HC(3)), 1.73 – 1.62 (m, 1H, H_bC(2)), 0.96 (s, 9H, H_3C(1')), 0.10 (s, 3H, H_3C(2')), 0.04 (s, 3H, H_3C(2')).

^{13}C NMR (126 MHz, $CDCl_3$) δ 167.6, 134.9, 131.5, 128.7, 127.0, 87.0, 70.5, 40.6, 29.4, 27.3, 17.8, 15.1, -7.0, -7.3.

HRMS (ESI'): $C_{18}H_{28}NOSi$ $[M+H]^+$ calc. 302.1940, found 302.1934.

Synthesis of *N*-(3-(*tert*-butyldimethylsilyl)pent-4-yn-1-yl)-3,5-dinitrobenzamide (16c)

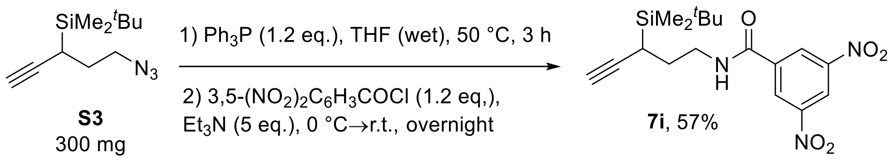

In a 25 mL round-bottomed flask, equipped with a Teflon-coated magnetic stirrer, (5-azidopent-1-yn-3-yl)(*tert*-butyl)dimethylsilane (**S3**, 300 mg, 1.34 mmol, 1.0 equiv) was dissolved in THF (wet; 10 mL). Ph_3P (423 mg, 1.61 mmol, 1.2 equiv) was added portion-wise to the stirred solution at ambient temperature. A Snyder column was installed on the flask neck, and the reaction mixture was heated in an oil bath (50 °C) for 3 hours. The reaction was cooled to 0 °C in an ice bath. Et_3N (0.94 mL, 6.71 mmol, 5.0 equiv) and 3,5-dinitrobenzoyl chloride (372 mg, 1.61 mmol, 1.2 equiv) was added, and the reaction was stirred for 18 hours (0→20 °C). For work-up the reaction mixture was then concentrated *in vacuo*, the residue was transferred to a separatory funnel with EtOAc (30 mL), washed with a saturated aqueous NH_4Cl solution (3 x 30 mL) and saturated aqueous NaCl solution, dried over anhydrous Na_2SO_4, filtered, and the filtrate was concentrated *in vacuo.* Normal-phase column chromatography (50-100% DCM/Hex) afforded the product **16c** as a yellow oil (405 mg, 74% purity (qNMR), η = 57%).

N-(3-(*tert*-Butyldimethylsilyl)pent-4-yn-1-yl)-3,5-dinitrobenzamide (**16c**):

Characterization data:

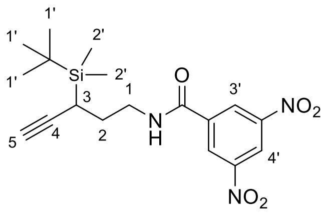

^{1}H NMR (500 MHz, $CDCl_3$) δ 9.17 (t, $^{4}J_{H\text{-}H}$ = 1.9 Hz, 1H, H-C(4')), 9.00 (d, $^{4}J_{H\text{-}H}$ = 1.9 Hz, 2H, H-C(3')), 7.22 (br s, 1H, H-N), 3.89 (dq, $^{2}J_{H\text{-}H}$ = 13.6 Hz, $^{3}J_{H\text{-}H}$ = 5.8 Hz, 1H, H_aC(1)), 3.63 (ddt, $^{2}J_{H\text{-}H}$ = 13.6 Hz, $^{3}J_{H\text{-}H}$ = 7.9, 5.1 Hz, 1H, H_bC(1)), 2.33 (d, $^{4}J_{H\text{-}H}$ = 2.6 Hz, 1H, HC(5)), 2.01 – 1.91 (m, 1H, H_aC(2)), 1.89 (dt, $^{3}J_{H\text{-}H}$ = 11.7 Hz, $^{3,4}J_{H\text{-}H}$ = 2.6 Hz, 1H, HC(3)), 1.79 (ddd, $^{2}J_{H\text{-}H}$ = 13.3 Hz, $^{3}J_{H\text{-}H}$ = 7.9, 5.8 Hz, 1H, H_bC(2)), 0.98 (s, 9H, H_3C(1')), 0.13 (s, 3H, H_3C(2')), 0.07 (s, 3H, H_3C(2')).

^{13}C NMR (126 MHz, $CDCl_3$) δ 162.5, 148.8, 138.2, 127.3, 121.1, 88.1, 71.3, 42.5, 29.1, 27.3, 17.8, 16.0, -7.0, -7.2.

HRMS (ESI'): $C_{18}H_{26}N_3O_5Si$ $[M+H]^+$ calc. 392.1642, found 392.1639.

Three-step procedure for the synthesis of *tert*-butyl 4-(*tert*-butyldimethylsilyl)hex-5-ynoate (7e)

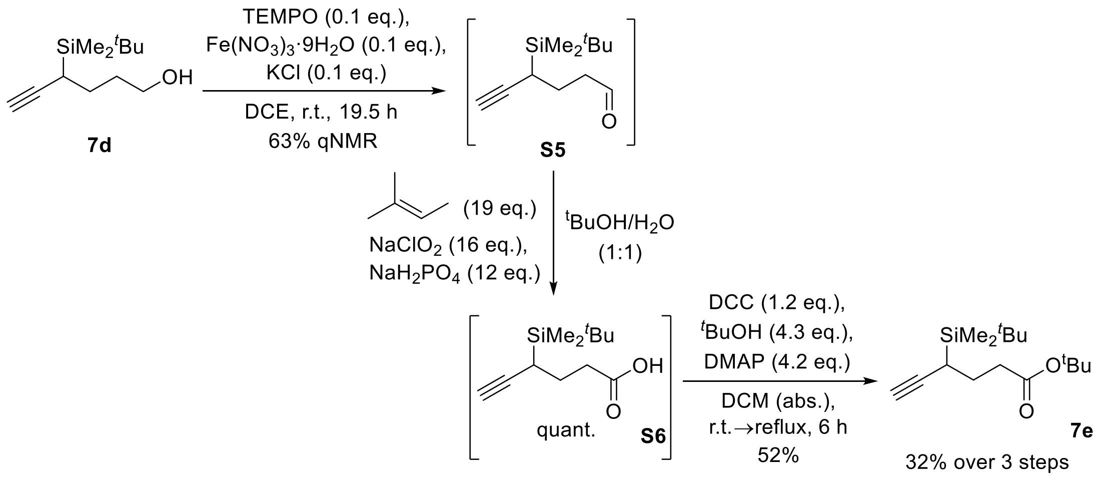

A 50 mL round-bottomed flask, equipped with a Teflon-coated magnetic stirrer, was charged with $Fe(NO_3)_3\cdot 9H_2O$ (191 mg, 0.47 mmol, 10 mol %), TEMPO (74 mg, 0.47 mmol, 10 mol %), KCl (35 mg, 0.47 mmol, 10 mol %), 4-(*tert*-butyldimethylsilyl)hex-5-yn-1-ol (**7d**) [1] (1.0 g, 4.71 mmol, 1.0 equiv) and DCE (20 mL). The reaction was stirred at ambient temperature for 19.5 hours under air [4]. For work-up the reaction mixture was filtered through a silica plug (the remaining product was removed from the silica gel using 100 mL EtOAc), and the filtrate was concentrated in vacuo. The crude aldehyde **S5** (1.068 g, 58% purity by NMR, $\eta_{NMR} = 63\%$) was used in the next step without additional purification.

Oxidation to the carboxylic acid was performed according to a scaled-up literature procedure [3]. In a 100 mL round-bottomed flask, equipped with a Teflon-coated magnetic stirrer, 4-(*tert*-butyldimethylsilyl)hex-5-ynal (**S5**, 617 mg, 2.93 mmol, 1.0 equiv) and 2-methylbut-2-ene (6 mL) were dissolved in *t*-BuOH (10 mL). To the stirred solution a mixture of $NaClO_2$ (4.120 g, 80%, 45.55 mmol, 16.0 equiv) and NaH_2PO_4 (4.250 g, 35.40 mmol, 12.0 equiv) in H_2O (10 mL) was added dropwise, and the resulting reaction mixture was stirred for 16 h at ambient temperature. The reaction mixture was concentrated in vacuo and then suspended in DCM (30 mL), washed with H_2O (3 × 30 mL), saturated aqueous NaCl (20 mL), dried over anhydrous Na_2SO_4, filtered, and concentrated in vacuo. The crude carboxylic acid **S6** (1.529 g, 43% purity by NMR, η_{NMR} = quant.) was used in the next step without additional purification.

The esterification reaction was performed in dry conditions and under an inert atmosphere (argon). A 25 mL two-necked round-bottomed flask (dried at 120 °C, degassed with argon), equipped with a Teflon-coated magnetic stirrer and a reflux condenser, was charged with DMAP (1.510 g, 12.36 mmol, 4.2 equiv) and closed with a rubber septum. The flask was evacuated and filled with argon using the Schlenk line (3×). Dry DCM (10 mL), *t*-BuOH (1.2 mL, 12.55 mmol, 4.3 equiv)) and 4-(*tert*-butyldimethylsilyl)hex-5-ynoic acid (**S6**, 664 mg, 2.93 mmol, 1.0 equiv) were added via syringe. DCC (704 mg, 3.41 mmol, 1.2 equiv) was added to the stirred mixture under argon flow. The resulting mixture was refluxed for 6 hours. For work-up the reaction mixture was concentrated in vacuo. The resulting crude mixture was purified by normal-phase column chromatography (1–2% EtOAc/Hex). The *tert*-butyl ester **7e** was obtained as a yellow oil (428 mg, η = 32% over 3 steps).

tert-Butyl 4-(*tert*-butyldimethylsilyl)hex-5-ynoate (**7e**):

Characterization data:

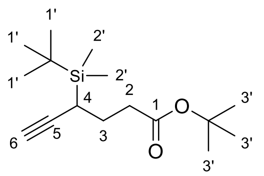

^{1}H NMR (500 MHz, CDCl$_3$) δ 2.60 (ddd, $^2J_{H\text{-}H}$ = 16.0 Hz, $^3J_{H\text{-}H}$ = 8.7, 4.8 Hz, 1H, H$_a$C(2)), 2.44 – 2.31 (m, 1H, H$_b$C(2)), 2.00 (d, $^4J_{H\text{-}H}$ = 2.7 Hz, 1H, HC(6)), 1.87 – 1.73 (m, 2H, H$_a$C(3) and HC(4)), 1.62 (dtd, $^2J_{H\text{-}H}$ = 17.5 Hz, $^3J_{H\text{-}H}$ = 8.8, 4.8 Hz, 1H, H$_b$C(3)), 1.45 (s, 9H, H$_3$C(3')), 0.96 (s, 9H, H$_3$C(1')), 0.09 (s, 3H, H$_3$C(2')), 0.04 (s, 3H, H$_3$C(2')).

^{13}C NMR (126 MHz, CDCl$_3$) δ 173.1, 86.5, 80.3, 69.9, 35.1, 28.3, 27.3, 25.4, 17.7, 16.5, -7.1, -7.3.

HRMS (ESI'): $C_{16}H_{31}O_2Si$ [M+H]$^+$ calc. 283.2093, found 283.2088.

Two step procedure for the synthesis of *N*-(4-(*tert*-butyldimethylsilyl)hex-5-yn-1-yl)aniline (7h)

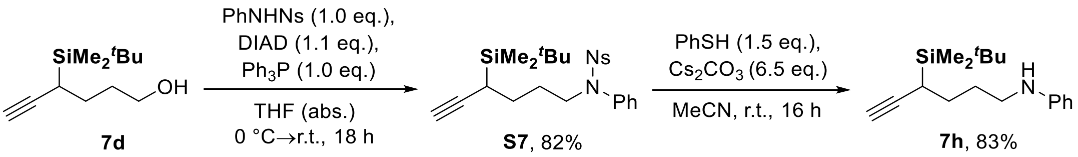

N-(4-(*tert*-Butyldimethylsilyl)hex-5-yn-1-yl)-4-nitro-*N*-phenylbenzenesulfonamide (**S7**):

Synthesis procedure:

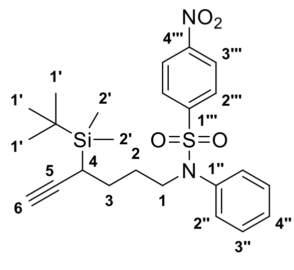

The reaction was performed in dry conditions and under an inert atmosphere (argon). A 50 mL round-bottomed flask (dried at 120 °C, degassed with argon), equipped with a Teflon-coated magnetic stirrer, was charged with Ph$_3$P (1.671 g, 6.37 mmol, 1.0 equiv), PhNHNs (1.773 g, 6.37 mmol, 1.0 equiv) and closed with a rubber septum. The flask was evacuated and filled with argon using the Schlenk line (3×). Dry THF and 4-(*tert*-butyldimethylsilyl)hex-5-yn-1-ol (**7d**, 1.353 g, 6.37 mmol, 1.0 equiv) was added to the mixture via syringe. The resulting solution was cooled to 0 °C in an ice bath. DIAD (1.38 mL, 7.01 mmol, 1.1 equiv) was added to the reaction mixture, and the reaction mixture was further stirred for 1 h at 0 °C, then 17 h at ambient temperature.

For work-up the reaction mixture was concentrated in vacuo, dissolved in 60% DCM/Hex (20 mL) and filtered through a silica plug, which was washed with additional 180 mL of 60% DCM/Hex. The filtrate was concentrated in vacuo. The impure product was precipitated from the concentrate by addition of 50% EtOH/H$_2$O (50 mL). The yellow, amorphous precipitate was collected by filtration. Further purification by column chromatography (30–50% DCM/Hex) afforded the product as an off-white amorphous solid (2.572 g, 96% purity (qNMR), η = 82%).

Characterization data:

^{1}H NMR (500 MHz, CDCl$_3$) δ 8.29 (d, $^3J_{H\text{-}H}$ = 8.8 Hz, 2H, H-C(3''')), 7.75 (d, $^3J_{H\text{-}H}$ = 8.8 Hz, 2H, H-C(2''')), 7.37 – 7.31 (m, 3H, H-C(2'', 4'')), 7.02 (dd, $^3J_{H\text{-}H}$ = 6.6, 3.0 Hz, 2H, H-C(3'')), 3.72 (dt, $^2J_{H\text{-}H}$ = 13.3 Hz, $^3J_{H\text{-}H}$ = 7.0 Hz, 1H, H$_a$C(1)), 3.54 (ddd, $^2J_{H\text{-}H}$ = 13.3 Hz, $^3J_{H\text{-}H}$ = 7.1,

5.2 Hz, 1H, $H_bC(1)$), 1.91 (d, $^4J_{H\text{-}H}$ = 2.8 Hz, 1H, HC(6)), 1.85 – 1.75 (m, 1H, $H_aC(2)$), 1.72 (dt, $^3J_{H\text{-}H}$ = 12.1 Hz, $^{3,4}J_{H\text{-}H}$ = 2.8 Hz, 1H, HC(4)), 1.62 – 1.51 (m, 2H, $H_bC(2)$ and $H_aC(3)$), 1.46 – 1.35 (m, 1H, $H_bC(3)$), 0.94 (s, 9H, $H_3C(1')$), 0.05 (s, 3H, $H_3C(2')$), -0.01 (s, 3H, $H_3C(2')$).

^{13}C NMR (126 MHz, $CDCl_3$) δ 150.13, 144.12, 138.17, 129.49, 128.94, 128.75, 128.60, 124.16, 86.59, 69.77, 50.23, 27.54, 27.26, 26.30, 17.71, 16.06, -7.07, -7.31.

HRMS (ESI'): $C_{24}H_{33}N_2O_4SSi^+$ $[M+H]^+$ calc. 473.1925, found 473.1932.

N-(4-(*tert*-Butyldimethylsilyl)hex-5-yn-1-yl)aniline (**7h**):

Synthesis procedure:

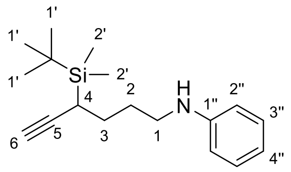

In a 100 mL round-bottomed flask, equipped with a Teflon-coated magnetic stirrer, *N*-(4-(*tert*-butyldimethylsilyl)hex-5-yn-1-yl)-4-nitro-*N*-phenylbenzenesulfonamide (**S7**, 1.75 g, 3.71 mmol, 1.0 equiv) was dissolved in MeCN (50 mL). Cs_2CO_3 (7.91 g, 24.28 mmol, 6.5 equiv) was added to the stirred solution at ambient temperature. The flask was sealed with a septum, and PhSH (0.57 mL, 5.57 mmol, 1.5 equiv) was added to the reaction dropwise via syringe. The reaction was then stirred for 16 h.
For work-up, H_2O (100 mL) was added, followed by extraction with DCM (3 × 100 mL). The combined organic layer was washed with saturated aqueous NaCl (200 mL), dried over anhydrous Na_2SO_4, filtered and concentrated in vacuo. The resulting crude mixture was purified by normal-phase column chromatography (10–30% DCM/Hex). The product **7h** was obtained as a yellow oil (885 mg, η = 83%).

Characterization data:

^{1}H NMR (500 MHz, $CDCl_3$) δ 7.17 (t, $^3J_{H\text{-}H}$ = 7.7 Hz, 2H, H-C(3")), 6.69 (t, $^3J_{H\text{-}H}$ = 7.7 Hz, 1H, H-C(4")), 6.61 (d, $^3J_{H\text{-}H}$ = 7.7 Hz, 2H, H-C(2")), 3.61 (br s, 1H, N-H), 3.22 – 3.05 (m, 2H, $H_2C(1)$), 2.08 – 1.95 (m, 2H, HC(6) and $H_aC(2)$), 1.82 – 1.67 (m, 2H, HC(4) and $H_bC(2)$), 1.67 – 1.46 (m, 2H, $H_2C(3)$), 0.96 (s, 9H, $H_3C(1')$), 0.09 (s, 3H, $H_3C(2')$), 0.02 (s, 3H, $H_3C(2')$).
^{13}C NMR (126 MHz, $CDCl_3$) δ 148.64, 129.36, 117.27, 112.84, 87.09, 69.74, 43.75, 29.48, 27.56, 27.31, 17.76, 16.83, -7.00, -7.22.
HRMS (ESI'): $C_{18}H_{30}NSi^+$ $[M+H]^+$ calc. 288.2142, found 288.2141.

Synthesis procedures / characterization data for arylation products

General Procedure B for the synthesis of silyl dienes

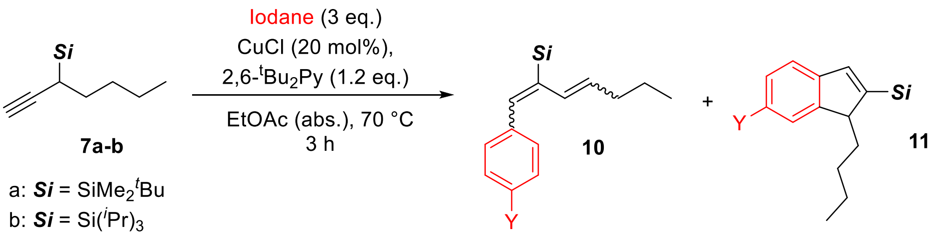

The reaction was performed in dry conditions and under an inert atmosphere (argon).

A 10 mL round-bottomed flask (dried at 120 °C, degassed with argon), equipped with a Teflon-coated magnetic stirrer, was charged with the iodane (3.0 equiv) and CuCl (20 mol %) and closed with a rubber septum. The flask was evacuated and filled with argon using the Schlenk line (3×). Dry EtOAc (5 mL), 2,6-*t*-Bu_2Py (1.2 equiv) and the silane **7** (0.48 mmol, 1 equiv) were added to the mixture at room temperature via syringe. The reaction mixture was stirred while heating in an oil bath (70 °C) for 3 h.

For work-up the resulting mixture was transferred to a separatory funnel using EtOAc (10 mL), washed with a saturated $NaHCO_3$ aq. solution (15 mL × 2) and saturated aq. NaCl solution (15 mL). The organic layer was separated and dried over anhydrous Na_2SO_4; the resulting suspension was filtered and the filtrate concentrated in vacuo. The concentrate was dissolved in hexane and filtered through a silica plug; the filtrate was then concentrated in vacuo. Subsequent reversed-phase column chromatography (50–100% $MeCN/H_2O$) afforded a mixture of diene **10** and silyl indene **11**. The diene **10** could be partially separated from the indene **11** by preparative HPLC.

Mixture of *tert*-butyldimethyl-1-phenylhepta-1,3-dien-2-yl)silane (**10a**) and *tert*-butyl(1-butyl-1*H*-inden-2-yl)dimethylsilane (**11a**)

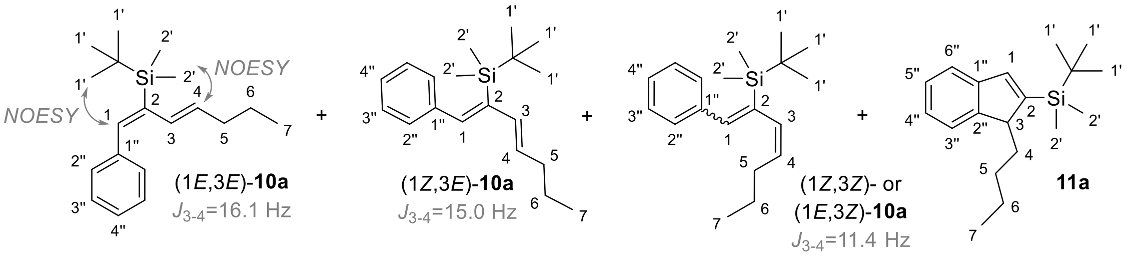

Diene/indene ratio for 1.90 mmol experiment:

In the crude mixture: the exact ratio of the minor isomers could not be determined by ^{1}H-NMR due to signal overlap with other impurities;

After C18 silica column: (1*E*,3*E*)-**10a** : (1*Z*,3*E*)-**10a** : (1*E*,3*Z*)- or (1*Z*,3*Z*)-**10a** : **11a** = 81:4:3:12;

After prepHPLC: (1*E*,3*E*)-**10a** : (1*Z*,3*E*)-**10a** : (1*E*,3*Z*)- or (1*Z*,3*Z*)-**10a** = 87:10:3

Synthesis procedure 1 (0.48 mmol of 7a):

The reaction was performed following **General procedure B** with *tert*-butyl(hept-1-yn-3-yl)dimethylsilane (**7a**, 100 mg, 0.48 mmol, 1.0 equiv), Ph_2IOTf (613 mg, 1.43 mmol, 3.0 equiv), CuCl (9 mg, 0.10 mmol, 20 mol %), 2,6-*t*-Bu_2Py (0.12 mL, 0.57 mmol, 1.2 equiv), abs. EtOAc (5 mL). Reversed-phase column chromatography (50–90% MeCN/H_2O) afforded a mixture of diene and indene ((1*E*,3*E*)-**10a**:(1*Z*,3*E*)-**10a**:(1*E*,3*Z*) or (1*Z*,3*Z*)-**10a**:**11a** = 85:3:2:10) as a yellow oil (51 mg, $\eta_{\mathbf{10a+11a}}$ = 37%).

Synthesis procedure 2 (1.90 mmol of 7a):

The reaction was performed following **General procedure B** with *tert*-butyl(hept-1-yn-3-yl)dimethylsilane (**7a**, 400 mg, 1.90 mmol, 1.0 equiv), Ph_2IOTf (2.453 g, 5.70 mmol, 3.0 equiv), CuCl (38 mg, 0.38 mmol, 20 mol %), 2,6-*t*-Bu_2Py (0.49 mL, 2.28 mmol, 1.2 equiv), abs. EtOAc (20 mL). Reversed-phase column chromatography (50–100% MeCN/H_2O) afforded a mixture of dienes and indene ((1*E*,3*E*)-**10a**:(1*Z*,3*E*)-**10a**:(1*E*,3*Z*) or (1*Z*,3*Z*)-**10a**:**11a** = 81:4:3:12) as a yellow oil (280 mg, $\eta_{\mathbf{10a+11a}}$ = 51%).

69 mg of the obtained diene/indene mixture was separated by preparative HPLC to afford the pure mixture of dienes ((1*E*,3*E*)-**10a**:(1*Z*,3*E*)-**10a**:(1*E*,3*Z*) or (1*Z*,3*Z*)-**10a** = 88:9:3) as a colorless oil (10 mg).

---

tert-Butyldimethyl((1*E*,3*E*)-1-phenylhepta-1,3-dien-2-yl)silane ((1*E*,3*E*)-**10a**):

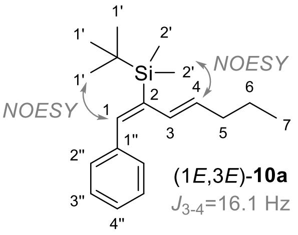

Characterization data:

^{1}H NMR (500 MHz, $CDCl_3$) δ 7.37 (d, $^3J_{H\text{-}H}$ = 7.4 Hz, 2H, H-C(2”)), 7.32 (t, $^3J_{H\text{-}H}$ = 7.4 Hz, 2H, H-C(3”)), 7.21 (t, $^3J_{H\text{-}H}$ = 7.4 Hz, 1H, H-C(4”)), 6.67 (s, 1H, HC(1)), 6.42 (d, $^3J_{H\text{-}H}$ = 16.1 Hz, 1H, HC(3)), 5.69 (dt, $^3J_{H\text{-}H}$ = 16.1, 7.1 Hz, 1H, HC(4)), 2.07 (q, $^3J_{H\text{-}H}$ = 7.1 Hz, 2H, $H_2C(5)$), 1.41 (sext, $^3J_{H\text{-}H}$ = 7.1 Hz, 2H, $H_2C(6)$), 0.98 – 0.88 (m, 3H, $H_3C(7)$), 0.93 (s, 9H, $H_3C(1')$) 0.21 (s, 6H, 2 x $H_3C(2')$).

^{13}C NMR (126 MHz, $CDCl_3$) δ 140.0, 139.6, 138.6, 134.2, 131.1, 129.6, 128.1, 126.9, 35.8, 27.4, 22.8, 17.7, 14.0, -4.2.

HRMS: $C_{19}H_{31}Si$ $[M+H]^+$ calc. 287.2195, found 287.2218.

Characteristic ^{1}H-NMR signal for (1*Z*,3*E*)-**10a** was observed at δ 6.25 (d, $^3J_{H\text{-}H}$ = 15.0 Hz, 1H, HC(3)); characteristic ^{1}H-NMR signals for (1*E*,3*Z*) or (1*Z*,3*Z*)-**10a** were observed at 6.12 (d, $^3J_{H\text{-}H}$ = 11.3 Hz, 1H, HC(3)), 5.25 (dt, $^3J_{H\text{-}H}$ = 11.3, 7.0 Hz, 1H, HC(4)). The remaining signals could not be identified accurately due to signal overlap and insufficient intensity on the correlation spectra.

---

tert-Butyl(1-butyl-1*H*-inden-2-yl)dimethylsilane (**11a**):

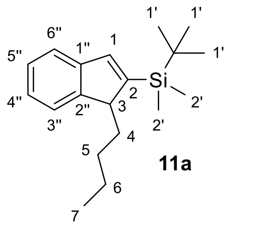

Spectral data matches those in the literature [2].

Mixture of triisopropyl(1-phenylhepta-1,3-dien-2-yl)silane (**10b**) and (1-butyl-1*H*-inden-2-yl)triisopropylsilane (**11b**)

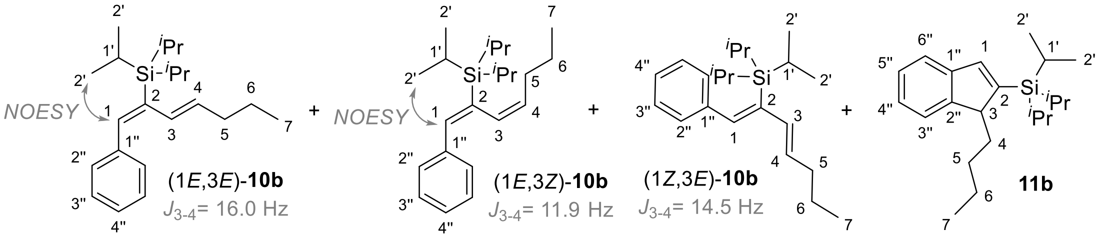

In the crude mixture: (1*E*,3*E*)-**10b** : (1*E*,3*Z*)-**10b** : **11b** = 63:6:31;
After C18 silica column: (1*E*,3*E*)-**10b** : (1*E*,3*Z*)-**10b** : **11b** = 62:5:33;
After prepHPLC: (1*E*,3*E*)-**10b** : (1*E*,3*Z*)-**10b** : (1*Z*,3*E*)-**10b** = 84:11:5

Synthesis procedure: The reaction was performed following **General pocedure B** with hept-1-yn-3-yltriisopropylsilane (**7b**, 120 mg, 0.48 mmol, 1.0 equiv), Ph_2IOTf (613 mg, 1.43 mmol, 3.0 equiv), CuCl (9 mg, 0.10 mmol, 20 mol %), 2,6-*t*-Bu_2Py (0.12 mL, 0.57 mmol, 1.2 equiv), abs. EtOAc (5 mL). Reversed-phase column chromatography (50–100% MeCN/H_2O) afforded a mixture of dienes and indene ((1*E*,3*E*)-**10b**:(1*E*,3*Z*)-**10b**:**11b** = 62:5:33) as a colorless oil (95 mg, $\eta_{\mathbf{10b+11b}}$ = 61%).

85 mg of the obtained dienes/indene mixture was separated by preparative HPLC to afford the pure mixture of dienes (1*E*,3*E*)-**10b**:(1*E*,3*Z*)-**10b**:(1*Z*,3*E*)-**10b** = 88:9:3) as a colorless oil (12 mg) and indene (**11b**) as a colorless oil (22 mg).

Triisopropyl((1*E*,3*E*)-1-phenylhepta-1,3-dien-2-yl)silane ((1*E*,3*E*)-**10b**):

Characterization data:

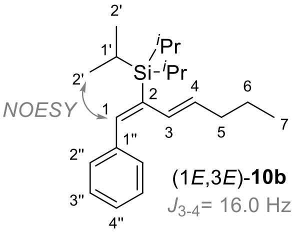

^{1}H NMR (500 MHz, $CDCl_3$): δ 7.39 (d, $^3J_{H\text{-}H}$ = 7.5 Hz, 2H, H-C(2”)), 7.30 (t, $^3J_{H\text{-}H}$ = 7.5 Hz, 2H, H-C(3”)), 7.20 (t, $^3J_{H\text{-}H}$ = 7.5 Hz, 1H, H-C(4”)), 6.68 (s, 1H, HC(1)), 6.33 (d, $^3J_{H\text{-}H}$ = 16.0 Hz, 1H, HC(3)), 5.57 (dt, $^3J_{H\text{-}H}$ = 16.0, 7.2 Hz, 1H, HC(4)), 2.04 (q, $^3J_{H\text{-}H}$ = 7.2 Hz, 2H, $H_2C(5)$), 1.39 (sext, $^3J_{H\text{-}H}$ = 7.2 Hz, 2H, $H_2C(6)$), 1.34 – 1.22 (m, 3H, HC(1’)), 1.12 (d, $^3J_{H\text{-}H}$ = 7.4 Hz, 18H, $H_3C(2’)$), 0.89 (t, $^3J_{H\text{-}H}$ = 7.2 Hz, 3H, $H_3C(7)$).

^{13}C NMR (126 MHz, $CDCl_3$): δ 140.0, 138.7, 138.2, 132.7, 131.3, 129.8, 128.0, 126.8, 35.8, 22.7, 19.0, 14.0, 11.8.

HRMS (ESI’): $C_{22}H_{37}Si$ $[M+H]^+$ calc. 329.2665, found 329.2576.

Triisopropyl((1*E*,3*Z*)-1-phenylhepta-1,3-dien-2-yl)silane ((1*E*,3*Z*)-**10b**):

Characterization data:

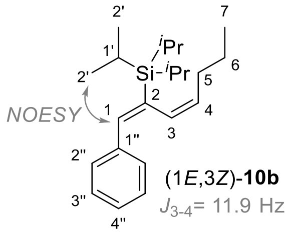

^{1}H NMR (500 MHz, $CDCl_3$): δ 7.42 (d, $^{3}J_{H-H}$ = 7.6 Hz, 2H, H-C(2”)), 7.26 – 7.23 (m, 2H, H-C(3”)), 7.15 (t, $^{3}J_{H-H}$ = 7.6 Hz, 1H, H-C(4”)), 6.66 (s, 1H, HC(1)), 6.08 (d, $^{3}J_{H-H}$ = 11.9 Hz, 1H, HC(3)), 5.14 (dt, $^{3}J_{H-H}$ = 11.9, 7.2 Hz, 1H, HC(4)), 1.60 (q, $^{3}J_{H-H}$ = 7.2 Hz, 2H, H_2C(5)), 1.34 – 1.22 (m, 3H, HC(1’)) 1.19 – 1.04 (m, 18H, H_3C(2’)), 1.02 – 0.92 (m, 2H, H_2C(6)), 0.66 (t, $^{3}J_{H-H}$ = 7.2 Hz, 3H, H_3C(7)).

^{13}C NMR (126 MHz, $CDCl_3$): δ 140.0, 139.1, 137.3, 129.5, 129.1, 129.0, 128.0, 126.7, 31.04, 29.9, 18.8, 14.2, 11.5.

HRMS (ESI’): $C_{22}H_{37}Si$ $[M+H]^+$ calc. 329.2665, found 329.2576.

Characteristic ^{1}H-NMR signals for (1*Z*,3*E*)-**10b** were observed at δ 6.26 (d, $^{3}J_{H-H}$ = 14.5 Hz, 1H, HC(3)) and 5.66 (dt, $^{3}J_{H-H}$ = 14.5, 6.4 Hz, 1H, HC(4)). The remaining signals could not be identified accurately due to signal overlap and insufficient intensity on the correlation spectra.

(1-Butyl-1*H*-inden-2-yl)triisopropylsilane (**11b**):

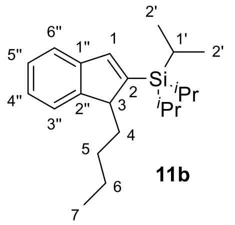

Spectral data matches those in the literature.[2]

Mixture of *tert*-butyldimethyl-1-(*p*-tolyl)hepta-1,3-dien-2-yl)silane (**10c**) and *tert*-butyl(1-butyl-6-methyl-1*H*-inden-2-yl)dimethylsilane (**11c**)

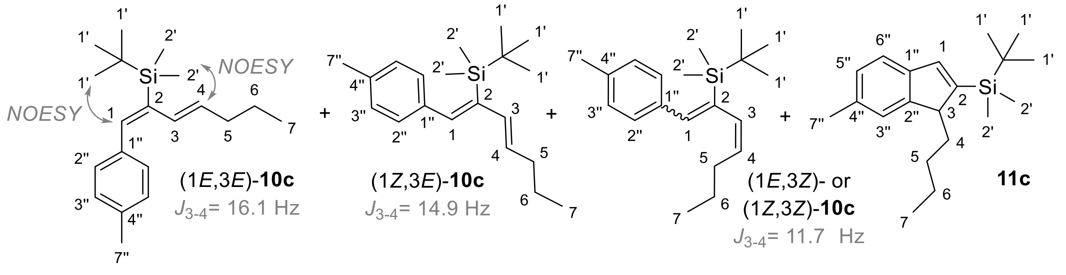

In the crude mixture: the exact ratio of the minor isomers could not be determined by ^{1}H-NMR due to signal overlap with other impurities;
After C18 silica column: (1*E*,3*E*)-**10c** : (1*Z*,3*E*)-**10c** : (1*E*,3*Z*)- or (1*Z*,3*Z*)-**10c** : **11c** = 74:6:5:15;
After prepHPLC: (1*E*,3*E*)-**10c** : (1*Z*,3*E*)-**10c** : (1*E*,3*Z*)- or (1*Z*,3*Z*)-**10c** = 83:11:6.

Synthesis procedure: The reaction was performed following **General procedure B** with *tert*-butyl(hept-1-yn-3-yl)dimethylsilane (**7a**, 100 mg, 0.48 mmol, 1.0 equiv), (*p*-Tol)MesIOTf (693 mg, 1.43 mmol, 3.0 equiv), CuCl (9 mg, 0.10 mmol, 20 mol %), 2,6-*t*-Bu_2Py (0.12 mL, 0.57 mmol, 1.2 equiv), abs. EtOAc (5 mL). Reversed-phase column chromatography (50–100%

MeCN/H_2O) afforded a mixture of dienes and indene ((1*E*,3*E*)-**10c**:(1*Z*,3*E*)-**10c**:(1*E*,3*Z*)- or (1*Z*,3*Z*)-**10c**:**11c** = 74:6:5:15) as a colorless oil (98 mg, $\eta_{\mathbf{10c+11c}}$ = 69%).

73 mg of the obtained diene/indene mixture was separated by preparative HPLC to afford the pure mixture of dienes ((1*E*,3*E*)-**10c** : (1*Z*,3*E*)-**10c** : (1*E*,3*Z*)- or (1*Z*,3*Z*)-**10c** = 83:11:6) as a colorless oil (3 mg) and indene **11c** as a colorless oil (2 mg).

---

tert-Butyldimethyl((1*E*,3*E*)-1-(*p*-tolyl)hepta-1,3-dien-2-yl)silane ((1*E*,3*E*)-**10c**):

Characterization data:

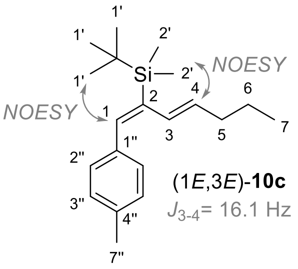

^{1}H NMR (500 MHz, $CDCl_3$) δ 7.27 (d, $^3J_{H\text{-}H}$ = 7.9 Hz, 2H, H-C(2'')), 7.12 (d, $^3J_{H\text{-}H}$ = 7.9 Hz, 2H, H-C(3'')), 6.63 (s, 1H, HC(1)), 6.42 (d, $^3J_{H\text{-}H}$ = 16.1 Hz, 1H, HC(3)), 5.67 (dt, $^3J_{H\text{-}H}$ = 16.1, 7.1 Hz, 1H, HC(4)), 2.34 (s, 3H, H_3C(7'')), 2.06 (q, $^3J_{H\text{-}H}$ = 7.1 Hz, 2H, H_2C(5)), 1.41 (sext, $^3J_{H\text{-}H}$ = 7.1 Hz, 2H, H_2C(6)), 0.94 – 0.89 (m, 3H, H_3C(7)), 0.92 (s, 9H, H_3C(1')), 0.20 (s, 6H, H_3C(2')).

^{13}C NMR (126 MHz, $CDCl_3$) δ 139.6, 139.1, 136.7, 135.7, 133.8, 131.3, 129.6, 128.8, 35.8, 27.4, 22.8, 21.4, 17.7, 14.0, -4.3.

HRMS (ESI'): $C_{20}H_{33}Si$ $[M+H]^+$ calc. 301.2352, found 301.2345.

Characteristic ^{1}H-NMR signal for (1*Z*,3*E*)-**10c** was observed at δ 6.24 (d, $^3J_{H\text{-}H}$ = 14.9 Hz, 1H, HC(3)); characteristic ^{1}H-NMR signals for (1*E*,3*Z*)- or (1*Z*,3*Z*)-**10c** were observed at 6.12 (d, $^3J_{H\text{-}H}$ = 11.7 Hz, 1H, HC(3)) and 5.26 (dt, $^3J_{H\text{-}H}$ = 11.7, 6.8 Hz, 1H, HC(4)). The remaining signals could not be identified accurately due to signal overlap and insufficient intensity on the correlation spectra.

---

tert-Butyl(1-butyl-6-methyl-1*H*-inden-2-yl)dimethylsilane (**11c**):

Characterization data:

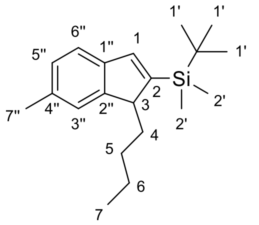

^{1}H NMR (500 MHz, $CDCl_3$) δ 7.25 – 7.19 (m, 2H, H-C(3'', 6'')), 7.08 – 7.02 (m, 2H, H-C(1, 5'')), 3.66 (t, $^3J_{H\text{-}H}$ = 4.5 Hz, 1H, HC(3)), 2.40 (s, 3H, H_3C(7'')), 2.08 (ddt, $^3J_{H\text{-}H}$ = 17.1 Hz, $^3J_{H\text{-}H}$ = 9.3, 4.5 Hz, 1H, H_aC(4)), 1.84 – 1.73 (m, 1H, H_bC(4)), 1.29 – 1.14 (m, 2H, H_2C(6)), 1.04 – 0.96 (m, 1H, H_aC(5)), 0.92 (s, 9H, H_3C(1')), 0.85 – 0.76 (m, 4H, H_bC(5), H_3C(7)), 0.22 (s, 3H, H_3C(2')), 0.18 (s, 3H, H_3C(2')).

^{13}C NMR (126 MHz, $CDCl_3$) δ 151.4, 149.6, 142.6, 142.5, 134.6, 127.2, 123.7, 120.4, 55.0, 30.9, 27.0, 26.6, 23.2, 21.7, 17.3, 14.1, -4.6, -5.1.

HRMS: $C_{20}H_{33}Si$ $[M+H]^+$ calc. 301.2352, found 301.2345.

1-(4-Bromophenyl)hepta-1,3-dien-2-yl)(*tert*-butyl)dimethylsilane (**10d**)

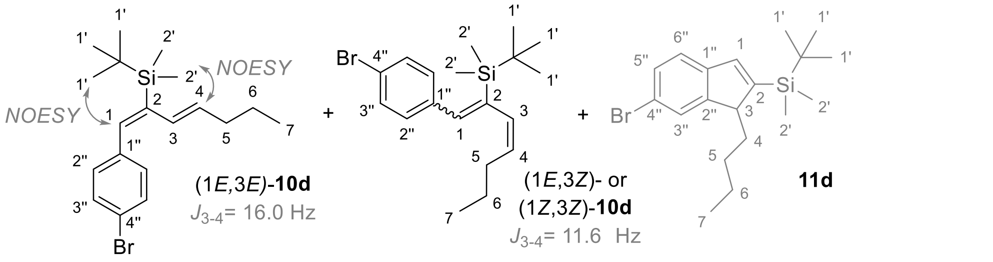

In the crude mixture: the exact ratio of the minor isomers could not be determined by ^{1}H-NMR due to signal overlap with other impurities.
After C18 silica column: (1*E*,3*E*)-**10d** : (1*E*,3*Z*)- or (1*Z*,3*Z*)-**10d** : **11d** = 93:5:2;
After prepHPLC: pure (1*E*,3*E*)-**10d**

Synthesis procedure: The reaction was performed following **General procedure B** with *tert*-butyl(hept-1-yn-3-yl)dimethylsilane (**7a**, 100 mg, 0.48 mmol, 1.0 equiv), (*p*-BrC_6H_4)MesIOTf (786 mg, 1.43 mmol, 3.0 equiv), CuCl (9 mg, 0.10 mmol, 20 mol %), 2,6-*t*-Bu_2Py (0.12 mL, 0.57 mmol, 1.2 equiv), abs. EtOAc (5 mL). Reversed-phase column chromatography (50–100% $MeCN/H_2O$) afforded the mixture of dienes and indene ((1*E*,3*E*)-**10d**:(1*E*,3*Z*)- or (1*Z*,3*Z*)-**10d**:**11d** = 93:5:2) as a yellow oil (65 mg, $\eta_{\mathbf{10d+11d}}$ = 37%).

59 mg of the obtained mixture was further purified by preparative HPLC to afford the *E*,*E*-diene (1*E*,3*E*)-**10d** as a colorless oil (10 mg).

((1*E*,3*E*)-1-(4-Bromophenyl)hepta-1,3-dien-2-yl)(tert-butyl)dimethylsilane ((1*E*,3*E*)-**10d**):

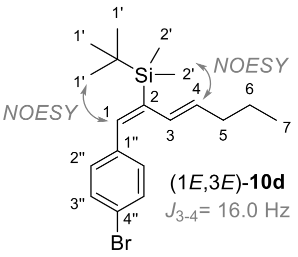

Characterization data:

^{1}H NMR (500 MHz, $CDCl_3$) δ 7.42 (d, $^3J_{H\text{-}H}$ = 8.4 Hz, 2H, H-C(3”)), 7.23 (d, $^3J_{H\text{-}H}$ = 8.4 Hz, 2H, H-C(2”)), 6.57 (s, 1H, HC(1)), 6.32 (d, $^3J_{H\text{-}H}$ = 16.0 Hz, 1H, HC(3)), 5.67 (dt, $^3J_{H\text{-}H}$ = 16.0, 7.1 Hz, 1H, HC(4)), 2.06 (q, $^3J_{H\text{-}H}$ = 7.1 Hz, 2H, H_2C(5)), 1.41 (sext, $^3J_{H\text{-}H}$ = 7.1 Hz, 2H, H_2C(6)), 0.95 – 0.87 (m, 3H, H_3C(7)), 0.92 (s, 9H, H_3C(1’)), 0.19 (s, 6H, H_3C(2’)).

^{13}C NMR (126 MHz, $CDCl_3$) δ 141.3, 138.1, 137.4, 134.7, 131.2, 131.2, 130.7, 120.7, 35.81, 27.4, 22.7, 17.6, 13.9, -4.4.

HRMS (ESI’): $C_{19}H_{31}Si$ $[M\text{-}Br+2H]^+$ calc. 287.2190, found 287.2217.

<u>Characteristic ^{1}H NMR signals for (1*E*,3*Z*)- or (1*Z*,3*Z*)-**10d**</u> were observed at δ 6.08 (d, $^3J_{H\text{-}H}$ = 11.6 Hz, 1H, HC(3)), 5.25 (dt, $^3J_{H\text{-}H}$ = 11.6, 7.1 Hz, 1H, HC(4)). The remaining signals could not be identified accurately due to signal overlap and insufficient intensity on the correlation spectra.

<u>Characteristic ^{1}H NMR signal for indene **11d**</u> was observed at 3.71 – 3.66 (m, 1H, HC(3)). It was assigned based on the chemical shifts and multiplicity of analogous compounds **11a-c**. The compound **11d** itself could not be isolated.

General Procedure C for the synthesis of tetrahydrofuran derivatives

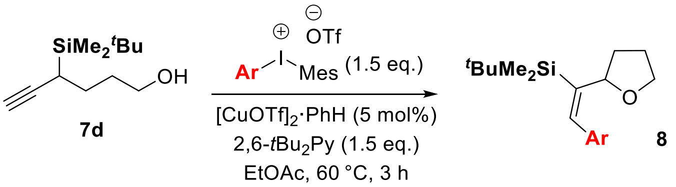

A 10 mL round-bottomed flask (dried at 120 °C, degassed with argon), equipped with a Teflon-coated magnetic stirrer, was charged with silane **7d** (100 mg, 0.47 mmol, 1.0 equiv), the iodane (0.71 mmol, 1.5 equiv) and $[CuOTf]_2$·PhH (12 mg, 0.024 mmol, 5 mol %) and closed with a rubber septum. The flask was evacuated and backfilled with argon using the Schlenk line (3×). Next, 2,6-*t*-Bu_2Py (135 mg, 152 µL, 0.71 mmol, 1.5 equiv) and dry EtOAc (5 mL) were added to the mixture at room temperature via syringe. The reaction mixture was stirred while heating in an oil bath (60 °C) for 3 h and subjected to GC–MS analysis.

For work-up, the resulting mixture was transferred to a separatory funnel using EtOAc[1] (10 mL), washed with a saturated $NaHCO_3$ aq. solution (10 mL × 2) and saturated aq. NaCl solution (10 mL). The organic layer was separated and dried over anhydrous Na_2SO_4; the resulting suspension was filtered and the filtrate concentrated in vacuo. The crude material was purified by column chromatography on silica.

(*E*)-*tert*-Butyldimethyl(2-phenyl-1-(tetrahydrofuran-2-yl)vinyl)silane (**8a**):

Synthesis procedure:

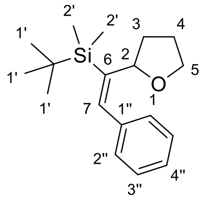

The reaction was performed following **General procedure C** with PhMesIOTf (290 mg, 0.56 mmol, 1.2 mmol), 2,6-*t*-Bu_2Py (117 µl, 0.56 mmol, 1.2 equiv). The crude mixture was purified by column chromatography on silica gel (10–20% DCM/Hex). Product **8a** was obtained as a white amorphous solid (108 mg, η = 80%).

<u>Modified conditions for gram-scale synthesis:</u>

The reaction was performed following a modified **General procedure C** with PhMesIOTf (2.1 g, 4.41 mmol, 1.2 equiv), CuCl (0.22 g, 0.21 mmol, 5 mol %), 4-(*tert*-butyldimethylsilyl)hex-5-yn-1-ol (**7d**, 0.89 g, 4.20 mmol, 1.0 equiv), 2,6-di-*tert*-butylpyridine (0.96 g, 5.03 mmol, 1.2 equiv) and EtOAc (50 mL). Purification by chromatography on silica gel (10–20% DCM/Hex) afforded the product **8a** with 76% yield (920 mg).

Spectral data matches those in the literature [3].

[1] DCM could also be used.

(E)-*tert*-Butyldimethyl(1-(tetrahydrofuran-2-yl)-2-(*p*-tolyl)vinyl)silane (**8b**):

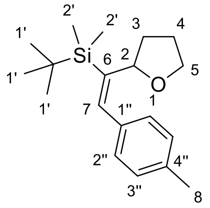

Synthesis procedure:

The reaction was performed following the **General procedure C** with mesityl(*p*-tolyl)-λ^3-iodaneyl trifluoromethanesulfonate (343 mg, 0.71 mmol, 1.5 equiv). The crude mixture was purified by column chromatography on silica (eluent: 50% DCM/Hex) to afford vinyl silane **8b** (98 mg, 83%) as a clear oil.

Characterization data:

^{1}H NMR (500 MHz, $CDCl_3$) δ 7.12 (d, $^3J_{H\text{-}H}$ = 7.9 Hz, 2H, H-C(2”)), 7.08 (d, $^3J_{H\text{-}H}$ = 7.9 Hz, 2H, H-C(3”)), 6.82 (s, 1H, HC(7)), 4.71 (ddd, $^3J_{H\text{-}H}$ = 9.8, 6.7 Hz, $^4J_{H\text{-}H}$ = 1.2 Hz, 1H, HC(2)), 3.92 (dt, $^3J_{H\text{-}H}$ = 8.1, 5.8, 1H, $H_aC(5)$), 3.62 (dt, $^3J_{H\text{-}H}$ = 8.3, 6.7, 1H, $H_bC(5)$), 2.24 (s, 3H, $H_3C(8)$) 2.03–1.98 (m, 1H, $H_aC(3)$), 1.97–1.82 (m, 2H, $H_2C(4)$), 1.67 (dtd, $^2J_{H\text{-}H}$ = 12.1 Hz, $^3J_{H\text{-}H}$ = 9.8, 7.8 Hz, 1H, $H_bC(3)$), 0.95 (s, 9H, $H_3C(1')$), 0.17 (s, 3H, $H_3C(2')$), 0.16 (s, 3H, $H_3C(2')$).

$^{13}C\{^1H\}$ NMR (126 MHz, $CDCl_3$) δ 143.9, 140.8, 136.5, 135.6, 128.7, 128.5, 79.3, 67.5, 33.2, 27.6, 26.0, 21.2, 17.5, -3.2, -3.8.

HRMS (APCI): $C_{19}H_{31}OSi^+$ $[M+H]^+$ calc. 303.2139, found 303.2130.

(E)-*tert*-Butyl(2-(3-methoxyphenyl)-1-(tetrahydrofuran-2-yl)vinyl)dimethylsilane (**8d**):

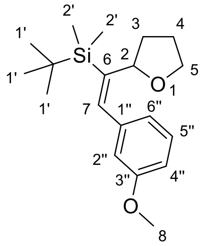

Synthesis procedure:

The reaction was performed following the **General procedure C** with mesityl(3-methoxyphenyl)-λ^3-iodaneyl trifluoromethanesulfonate (355 mg, 0.71 mmol, 1.5 equiv). The crude mixture was purified by column chromatography on silica (eluent: 50% DCM/Hex) to afford vinyl silane **8d** (104 mg, 70%) as a clear oil.

Characterization data:

^{1}H NMR (500 MHz, $CDCl_3$) δ 7.23 (t, $^3J_{H\text{-}H}$ = 7.8 Hz, 1H, H-C(5“)), 6.83 (s, 1H, HC(7)), 6.80–6.75 (m, 2H, H-C(4“, 6“)), 6.72 (t, $^4J_{H\text{-}H}$ = 2.2, 1H, H-C(2”)), 4.69 (ddd, $^3J_{H\text{-}H}$ = 9.7, 6.7, $^4J_{H\text{-}H}$ = 1.3 Hz, 1H, HC(2)), 3.93 (dt, $^3J_{H\text{-}H}$ = 8.1, 6.0 Hz, 1H, $H_aC(5)$), 3.80 (s, 3H, $H_3C(8)$), 3.62 (dt, $^3J_{H\text{-}H}$ = 8.4, 6.7 Hz, 1H, $H_bC(5)$), 2.07–1.98 (m, 1H, $H_aC(3)$), 1.96–1.83 (m, 2H, $H_2C(4)$), 1.67 (dtd, $^2J_{H\text{-}H}$ = 12.1 Hz, $^3J_{H\text{-}H}$ = 9.7, 7.8 Hz, 1H, $H_bC(3)$), 0.96 (s, 9H, $H_3C(1')$), 0.17 (s, 3H, $H_3C(2')$), 0.17 (s, 3H, $H_3C(2')$).

$^{13}C\{^1H\}$ NMR (126 MHz, $CDCl_3$) δ 159.4, 145.0, 140.7, 140.1, 129.1, 121.1, 114.1, 112.4, 79.5, 67.6, 55.3, 33.4, 27.7, 26.1, 17.6, -3.1, -3.7.

HRMS (APCI): $C_{19}H_{31}O_2Si^+$ $[M+H]^+$ calc. 319.2088, found 319.2079.

(*E*)-(2-(4-Bromophenyl)-1-(tetrahydrofuran-2-yl)vinyl)(*tert*-butyl)dimethylsilane (**8f**):

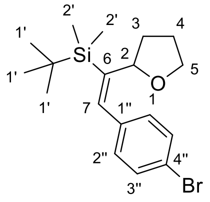

Synthesis procedure:

The reaction was performed following the **General procedure C** with (4-bromophenyl)(mesityl)-λ^3-iodaneyl trifluoromethanesulfonate (390 mg, 0.71 mmol, 1.5 equiv). The crude mixture was purified by column chromatography on silica (eluent: 60% DCM/Hex) to afford vinyl silane **8f** (130 mg, 80%) as a clear oil.

Characterization data:

^{1}H NMR (500 MHz, $CDCl_3$) δ 7.43 (d, $^3J_{H\text{-}H}$ = 8.3 Hz, 2H, H-C(3”)), 7.05 (d, $^3J_{H\text{-}H}$ = 8.3 Hz, 2H, H-C(2”)), 6.76 (s, 1H, HC(7)), 4.62 (ddd, $^3J_{H\text{-}H}$ = 9.7, 6.4 Hz, $^4J_{H\text{-}H}$ = 1.2 Hz, 1H, HC(2)), 3.91 (dt, $^3J_{H\text{-}H}$ = 8.1, 5.9 Hz, 1H, $H_aC(5)$), 3.62 (dt, $^3J_{H\text{-}H}$ = 8.1, 6.7, 1H, $H_bC(5)$), 2.01–1.82 (m, 3H, $H_aC(3)$, $H_2C(4)$), 1.65 (dtd, $^2J_{H\text{-}H}$ = 12.1 Hz, $^3J_{H\text{-}H}$ = 9.7, 7.8 Hz, 1H, $H_bC(3)$), 0.95 (s, 9H, $H_3C(1')$), 0.17 (s, 3H, $H_3C(2')$), 0.16 (s, 3H, $H_3C(2')$).

$^{13}C\{^1H\}$ NMR (126 MHz, $CDCl_3$) δ 145.9, 139.4, 137.4, 131.1, 130.1, 120.7, 79.2, 67.6, 33.2, 27.5, 25.9, 17.4, -3.3, -3.9.

HRMS (APCI): $C_{18}H_{26}BrOSi^-$ [M-H]$^-$ calc. 365.0931, found 365.0920.

(*E*)-*tert*-Butyl(2-(4-fluorophenyl)-1-(tetrahydrofuran-2-yl)vinyl)dimethylsilane (**8h**):

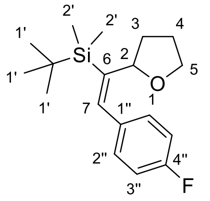

Synthesis procedure:

The reaction was performed following the **General Ppocedure C** with (4-fluorophenyl)(mesityl)-λ^3-iodaneyl trifluoromethanesulfonate (345 mg, 0.71 mmol, 1.5 equiv). The crude mixture was purified by column chromatography on silica (eluent: 60% DCM/Hex) to afford vinyl silane **8h** (112 mg, 78%) as a clear oil.

Characterization data:

^{1}H NMR (500 MHz, $CDCl_3$) δ 7.15 (dd, $^3J_{H\text{-}H}$ = 8.5 Hz, $^5J_{H\text{-}F}$ = 5.7 Hz, 2H, H-C(2”)), 7.00 (t, $^3J_{H\text{-}H,\ H\text{-}F}$ = 8.5 Hz, 2H, H-C(3”)), 6.80 (s, 1H, HC(7)), 4.64 (ddd, $^3J_{H\text{-}H}$ = 9.7, 6.4 Hz, $^4J_{H\text{-}H}$ = 1.3 Hz, 1H, HC(2)), 3.92 (dt, $^3J_{H\text{-}H}$ = 8.2, 5.9 Hz, 1H, $H_aC(5)$), 3.62 (dt, $^3J_{H\text{-}H}$ = 8.2, 6.6 Hz, 1H, $H_bC(5)$), 2.03–1.82 (m, 3H, $H_aC(3)$, $H_2C(4)$), 1.66 (dtd, $^2J_{H\text{-}H}$ = 11.9 Hz, $^3J_{H\text{-}H}$ = 9.7, 7.8 Hz, 1H, $H_bC(3)$), 0.95 (s, 9H, $H_3C(1')$), 0.17 (s, 3H, $H_3C(2')$), 0.16 (s, 3H, $H_3C(2')$).

$^{13}C\{^1H\}$ NMR (126 MHz, $CDCl_3$) δ 161.8 (d, $^1J_{C\text{-}F}$ = 246 Hz), 145.0, 139.8, 134.6 (d, $^4J_{C\text{-}F}$ = 3 Hz), 130.2 (d, $^3J_{C\text{-}F}$ = 8 Hz), 115.0 (d, $^2J_{C\text{-}F}$ = 21 Hz), 114.9, 79.3, 67.7, 33.3, 27.6, 26.1, -3.1, -3.8.

HRMS (APCI): $C_{18}H_{26}FOSi^-$ [M-H]$^-$ calc. 305.1731, found 305.1722.

(E)-4-(2-(*tert*-Butyldimethylsilyl)-2-(tetrahydrofuran-2-yl)vinyl)benzonitrile (**8j**):

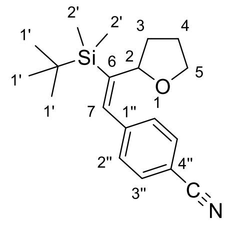

Synthesis procedure:

The reaction was performed following the **General procedure C** with ((4-cyanophenyl)(mesityl)-λ^3-iodaneyl trifluoromethanesulfonate (350 mg, 0.71 mmol, 1.5 equiv). The crude mixture was purified by column chromatography on silica (eluent: 60% DCM/Hex) to afford vinyl silane **8j** (18 mg, 12%) as a clear oil.

Characterization data:

^{1}H NMR (500 MHz, $CDCl_3$) δ 7.60 (d, $^3J_{H\text{-}H}$ = 8.1 Hz, 2H, H-C(3”)), 7.27 (d, $^3J_{H\text{-}H}$ = 8.1 Hz, 2H, H-C(2”)), 6.81 (s, 1H, HC(7)), 4.57 (ddd, $^3J_{H\text{-}H}$ = 9.6, 6.2 Hz, $^4J_{H\text{-}H}$ = 1.3 Hz, 1H, HC(2)), 3.90 (dt, $^3J_{H\text{-}H}$ = 8.0, 6.2 Hz, 1H, $H_aC(5)$), 3.62 (dt, $^3J_{H\text{-}H}$ = 8.2, 6.5 Hz, 1H, $H_bC(5)$), 2.01–1.81 (m, 3H, $H_aC(3)$, $H_2C(4)$), 1.66 (dtd, 1H, $^2J_{H\text{-}H}$ = 12.0 Hz, $^3J_{H\text{-}H}$ = 9.6, 7.8 Hz, $H_bC(3)$), 0.95 (s, 9H, $H_3C(1')$), 0.18 (s, 3H, $H_3C(2')$), 0.17 (s, 3H, $H_3C(2')$).

$^{13}C\{^1H\}$ (126 MHz, $CDCl_3$) δ 148.6, 143.4, 138.7, 132.0, 129.2, 119.1, 110.4, 79.3, 67.8, 33.4, 27.6, 26.0, 17.6, -3.3, -3.9.

HRMS (APCI): $C_{19}H_{28}NOSi^+$ $[M+H]^+$ calc. 314.1935, found 314.1925.

(E)-1-(4-(2-(*tert*-Butyldimethylsilyl)-2-(tetrahydrofuran-2-yl)vinyl)phenyl)ethan-1-one (**8k**):

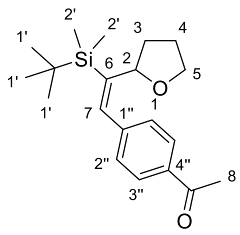

Synthesis procedure:

The reaction was performed following the **General procedure C** with (4-acetylphenyl)(mesityl)-λ^3-iodaneyl trifluoromethanesulfonate (365 mg, 0.71 mmol, 1.5 equiv). The crude mixture was purified by column chromatography on silica (eluent: 60–70% DCM/Hex) to afford vinyl silane **8k** (23 mg, 15%) as a clear oil.

Characterization data:

^{1}H NMR (500 MHz, $CDCl_3$) δ 7.91 (d, $^3J_{H\text{-}H}$ = 8.2 Hz, 2H, H-C(3”)), 7.27 (d, $^3J_{H\text{-}H}$ = 8.2 Hz, 2H, H-C(2”)), 6.85 (s, 1H, HC(7)), 4.63 (ddd, $^3J_{H\text{-}H}$ = 9.7, 6.6 Hz, $^4J_{H\text{-}H}$ = 1.4 Hz, 1H, HC(2)), 3.92 (dt, $^3J_{H\text{-}H}$ = 8.2, 6.0 Hz, 1H, $H_aC(5)$), 3.60 (dt, $^3J_{H\text{-}H}$ = 8.3, 6.7 Hz, 1H, $H_bC(5)$), 2.60 (s, 3H, $H_3C(8)$), 2.03–1.82 (m, 3H, $H_aC(3)$, $H_2C(4)$), 1.67 (dtd, 1H, $^2J_{H\text{-}H}$ = 11.8 Hz, $^3J_{H\text{-}H}$ = 9.7, 7.8 Hz, $H_bC(3)$), 0.96 (s, 9H, $H_3C(1')$), 0.18 (s, 3H, $H_3C(2')$), 0.18 (s, 3H, $H_3C(2')$).

$^{13}C\{^1H\}$ NMR (126 MHz, $CDCl_3$) δ 197.7, 147.4, 143.5, 139.5, 135.4, 128.6, 128.2, 79.3, 67.6, 33.3, 27.5, 26.6, 25.9, 17.5, -3.3, -3.9.

HRMS (APCI): $C_{20}H_{31}O_2Si^+$ $[M+H]^+$ calc. 331.2088, found 331.2081.

Methyl (*E*)-4-(2-(*tert*-butyldimethylsilyl)-2-(tetrahydrofuran-2-yl)vinyl)benzoate (**8l**):

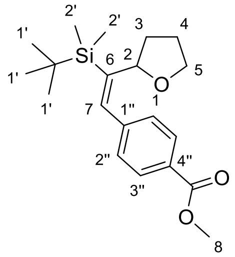

Synthesis procedure:

The reaction was performed following the **General procedure C** with methyl 4-(mesityl(((trifluoromethyl)sulfonyl)oxy)-λ^3-iodaneyl)benzoate (375 mg, 0.71 mmol, 1.5 equiv). The crude mixture was purified by column chromatography on silica (eluent: 50-60% DCM/Hex) to afford vinyl silane **8l** (112 mg, 68%) as a clear oil.

Characterization data:

^{1}H NMR (500 MHz, $CDCl_3$) δ 7.98 (d, $^3J_{H\text{-}H}$ = 8.4 Hz, 2H, H-C(3”)), 7.24 (d, $^3J_{H\text{-}H}$ = 8.4 Hz, 2H, H-C(2”)), 6.85 (s, 1H, HC(7)), 4.62 (ddd, $^3J_{H\text{-}H}$ = 9.7, 6.5 Hz, $^4J_{H\text{-}H}$ = 1.2 Hz, 1H, HC(2)), 3.93–3.87 (m, 1H, $H_aC(5)$), 3.91 (s, 3H, $H_3C(8)$), 3.82 (dt, $^3J_{H\text{-}H}$ = 8.2, 6.5 Hz, 1H, $H_bC(5)$), 2.04–1.82 (m, 3H, $H_aC(3)$, $H_2C(4)$), 1.66 (dtd, $^2J_{H\text{-}H}$ = 12.0 Hz, $^3J_{H\text{-}H}$ = 9.7, 7.9 Hz, 1H, $H_bC(3)$), 0.96 (s, 9H, $H_3C(1')$), 0.18 (s, 3H, $H_3C(2')$), 0.17 (s, 3H, $H_3C(2')$).

^{13}C{^{1}H} NMR (126 MHz, $CDCl_3$) δ 167.1, 147.2, 143.5, 139.7, 129.5, 128.6, 128.4, 79.4, 67.7, 52.2, 33.4, 27.6, 26.0, 17.6, -3.2, -3.8.

HRMS (APCI): $C_{16}H_{21}O_3Si^+$ [M-C(CH_3)$_3$]$^+$ calc. 289.1254, found 289.1259.

(*E*)-*tert*-Butyldimethyl(1-(tetrahydrofuran-2-yl)-2-(4-(trifluoromethyl)phenyl)vinyl)silane (**8m**):

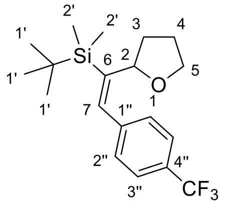

Synthesis procedure:

The reaction was performed following the **General procedure C** with mesityl(4-(trifluoromethyl)phenyl)-λ^3-iodaneyl trifluoromethanesulfonate (383 mg, 0.71 mmol, 1.5 equiv). The crude mixture was purified by column chromatography on silica (eluent: 20–30% DCM/Hex) to afford vinyl silane **8m** (104 mg, 62%) as a clear oil.

Characterization data:

^{1}H NMR (500 MHz, $CDCl_3$) δ 7.56 (d, $^3J_{H\text{-}H}$ = 8.1 Hz, 2H, H-C(2”)), 7.28 (d, $^3J_{H\text{-}H}$ = 8.1 Hz, 2H, H-C(3”)), 6.84 (s, 1H, HC(7)), 4.60 (ddd, $^3J_{H\text{-}H}$ = 9.7, 6.3 Hz, $^3J_{H\text{-}H}$ = 1.3 Hz, 1H, HC(2)), 3.91 (dt, $^3J_{H\text{-}H}$ = 8.3, 6.1 Hz, 1H, $H_aC(5)$), 3.62 (dt, $^3J_{H\text{-}H}$ = 8.3, 6.5 Hz, 1H, $H_bC(5)$), 2.01–1.81 (m, 3H, $H_aC(3)$, $H_2C(4)$), 1.67 (dtd, $^2J_{H\text{-}H}$ = 12.1 Hz, $^3J_{H\text{-}H}$ = 9.7, 7.7 Hz, 1H, $H_bC(3)$), 0.96 (s, 9H, $H_3C(1')$), 0.18 (s, 3H, $H_3C(2')$), 0.18 (s, 3H, $H_3C(2')$).

^{13}C{^{1}H} (126 MHz, $CDCl_3$) δ 147.3, 142.3, 139.3, 128.9 (q, J = 32), 128.8, 125.1 (q, J = 3), 124.4 (q, J = 272), 79.3, 67.7, 33.4, 27.6, 26.0, 17.6, -3.2, -3.8.

HRMS (APCI): $C_{19}H_{28}F_3OSi^+$ [M+H]$^+$ calc. 357.1856, 357.1857.

(E)-*tert*-Butyldimethyl(1-(tetrahydrofuran-2-yl)-2-(thiophen-2-yl)vinyl)silane (**8o**):

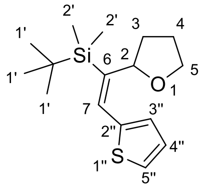

Synthesis procedure:

The reaction was performed following the **General Procedure C** with di(thiophen-2-yl)-λ^3-iodaneyl 4-methylbenzenesulfonate (330 mg, 0.71 mmol, 1.5 equiv). The crude mixture was purified by column chromatography on silica (eluent: 40% DCM/Hex) to afford vinyl silane **8o** (47 mg, 34%) as a clear oil.

Characterization data:

^{1}H NMR (500 MHz, $CDCl_3$) δ 7.29 (d, $^3J_{H-H}$ = 5.0 Hz, 1H, H-C(5”)), 7.00 (dd, $^3J_{H-H}$ = 5.0, 3.5 Hz, 1H, H-C(4”)), 6.98 (d, $^3J_{H-H}$ = 3.5 Hz, 1H, H-C(3”)), 6.82 (s, 1H, HC(7)), 5.07 (ddd, $^3J_{H-H}$ = 9.7, 6.5 Hz, $^4J_{H-H}$ = 1.6 Hz, 1H, HC(2)), 4.0 (td, $^3J_{H-H}$ = 7.7, 7.1 Hz, 1H, $H_aC(5)$), 3.74 (q, $^3J_{H-H}$ = 7.7 Hz, 1H, $H_bC(5)$), 2.36 (dq, $^2J_{H-H}$ = 12.5 Hz, $^3J_{H-H}$ = 6.5 Hz, 1H, $H_aC(3)$), 1.98 (dtd, $^3J_{H-H}$ = 7.7, 6.5, 5.9 Hz, 1H, $H_2C(4)$), 1.56 (ddt, $^2J_{H-H}$ = 12.5 Hz, $^3J_{H-H}$ = 9.7, 5.9 Hz, 1H, $H_bC(3)$), 0.94 (s, 9H, $H_3C(1')$), 0.16 (s, 3H, $H_3C(2')$), 0.15 (s, 3H, $H_3C(2')$).

$^{13}C\{^1H\}$ NMR (126 MHz, $CDCl_3$) δ 143.5, 140.8, 131.1, 128.7, 126.9, 126.5, 80.2, 67.8, 32.0, 27.7, 26.0, 17.7, -3.0, -3.9.

HRMS (APCI): $C_{16}H_{27}OSSi^+$ $[M+H]^+$ calc. 295.1546, found 295.1537.

Synthesis of (E)-5-(1-(*tert*-butyldimethylsilyl)-2-phenylvinyl)dihydrofuran-2(3H)-one (8t)

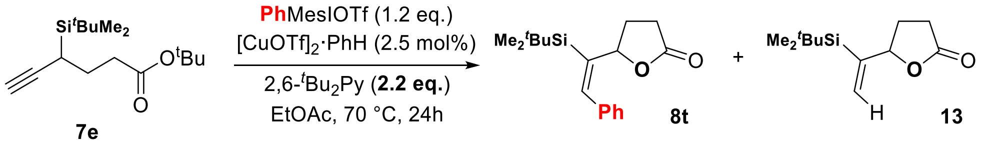

(E)-5-(1-(*tert*-butyldimethylsilyl)-2-phenylvinyl)dihydrofuran-2(3H)-one (**8t**):

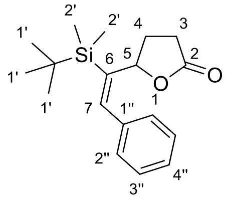

Synthesis procedure:

The reaction was performed following a modified **General procedure C** with *tert*-butyl 4-(tert-butyldimethylsilyl)hex-5-ynoate (**7e**) (134 mg, 0.48 mmol, 1.0 equiv), PhMesIOTf (269 mg, 0.57 mmol, 1.2 equiv), $[CuOTf]_2$·PhH (6 mg, 0.01 mmol, 2.5 mol %), 2,6-t-Bu_2Py (0.23 mL, 1.05 mmol, 2.2 equiv), abs. EtOAc (5 mL). Reaction temperature – 70 °C; reaction time – 24 h.

Column chromatography (50–70% DCM/Hex) afforded a mixture of (E)-5-(1-(*tert*-butyldimethylsilyl)-2-phenylvinyl)dihydrofuran-2(3H)-one (**8t**) and 5-(1-(*tert*-butyldimethylsilyl)vinyl)dihydrofuran-2(3H)-one (**13**) [3] as a yellowish oil (45 mg, n_{P1}/n_{P2} = 84:16 by NMR, $\eta_{8t\ (NMR)}$ = 27%). The arylated product **8t** could be separated from the mixture by using preparative HPLC and isolated as a colorless oil (32 mg, η = 22%).

Characterization data:

^{1}H NMR (500 MHz, DCM-d_2) δ 7.37 (t, $^3J_{H\text{-}H}$ = 7.4 Hz, 2H, H-C(3”)), 7.29 (t, $^3J_{H\text{-}H}$ = 7.4 Hz, 1H, H-C(4”)), 7.17 (d, $^3J_{H\text{-}H}$ = 7.4 Hz, 2H, H-C(2”)), 7.00 (s, 1H, HC(7)), 5.42 (dd, $^3J_{H\text{-}H}$ = 9.8, 7.4 Hz, 1H, HC(5)), 2.54 – 2.44 (m, 2H, H_2C(3)), 2.35 (dtd, $^2J_{H\text{-}H}$ = 14.9 Hz, $^3J_{H\text{-}H}$ = 7.4, 3.9 Hz, 1H, H_aC(4)), 2.14 – 1.99 (m, 1H, H_bC(4)), 0.98 (s, 9H, H_3C(1’)), 0.21 (s, 3H, H_3C(2’)), 0.19 (s, 3H, H_3C(2’)).

^{13}C NMR (126 MHz, $CDCl_3$) δ 177.2, 143.1, 141.6, 137.6, 128.6, 128.2, 127.7, 81.0, 30.0, 29.3, 27.4, 17.6, -3.3, -3.9.

HRMS: $C_{18}H_{27}O_2Si^+$ $[M+H]^+$ calc. 303.1780, found 303.1778.

5-(1-(*tert*-Butyldimethylsilyl)vinyl)dihydrofuran-2(3*H*)-one (**13**):

Spectral data matches those in the literature [3].

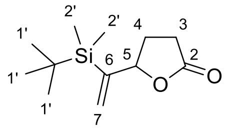

Synthesis of (2-(1-(*tert*-butyldimethylsilyl)-2-phenylvinyl)pyrrolidin-1-yl)(phenyl)methanone (8u)

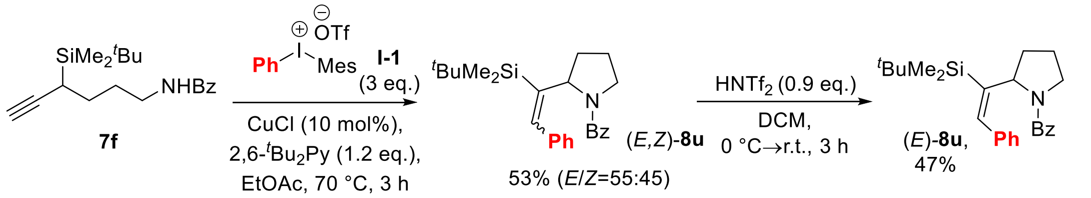

The reaction was performed following a modified **General procedure B** with *N*-(4-(*tert*-butyldimethylsilyl)hex-5-yn-1-yl)benzamide (**7f**) [3] (50 mg, 0.16 mmol, 1.0 equiv), PhMesIOTf (225 mg, 0.48 mmol, 3.0 equiv), CuCl (2 mg, 0.02 mmol, 10 mol %), 2,6-*t*-Bu_2Py (36 µl, 0.19 mmol, 1.2 equiv), abs. EtOAc (1.7 mL). Reaction time – 3 h.

Purification & work-up: the resulting mixture was transferred to a separatory funnel using EtOAc (5 mL), washed with a saturated $NaHCO_3$ aq. solution (7 mL × 2) and saturated aq. NaCl solution (7 mL). The organic layer was separated and dried over anhydrous Na_2SO_4; the resulting suspension was filtered and the filtrate concentrated in vacuo. The crude mixture was filtered through a silica plug (rinsed with DCM to remove most of the excess reagents and impurities, then with EtOAc to elute the product); the filtrate containing the product was collected separately and concentrated in vacuo. Reversed-phase column chromatography (50–70% MeCN/H_2O) of the filtrated product gave a mixture of (*E*)- and (*Z*)-(2-(1-(*tert*-butyldimethylsilyl)-2-phenylvinyl)pyrrolidin-1-yl)(phenyl)methanone (**8u**) as a yellow oil (33 mg, 53% yield, *E*:*Z*=55:45[2]).

[2] n/n determined by ^{1}H NMR in DMSO-d_6 at 80 °C.

The obtained (*E*/*Z*)-mixture of product **8u** (29 mg, 0.07 mmol, 1.0 equiv) was transferred to a 10 mL round-bottomed flask, equipped with a Teflon-coated magnetic stirrer, and dissolved in DCM (2.5 mL; non-dry). The solution was cooled to 0 °C in an ice bath. $HNTf_2$ (18 mg, 0.06 mmol, 0.9 equiv) was added and the resulting solution was stirred for 3 h.[3] The reaction was quenched by the addition of saturated aq. $NaHCO_3$ solution (5 mL), transferred to a separatory funnel, where the organic layer was separated and washed with a saturated aq. NaCl solution (5 mL), then dried over anhydrous Na_2SO_4, filtered and concentrated in vacuo. Normal-phase column chromatography (0–1% EtOAc/DCM) afforded the product (*E*)-**8u** as a colorless oil (14 mg, 47%).

(*E*)-(2-(1-(*tert*-Butyldimethylsilyl)-2-phenylvinyl)pyrrolidin-1-yl)(phenyl)methanone (***E*-8u**):

The product is observed on NMR as a mixture of rotamers (α and β. Only one rotamer is observed in DMSO-d_6 at 80 °C due to coalescence.

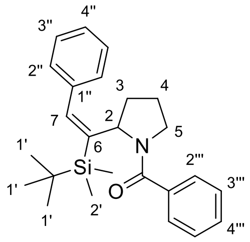

Characterization data:

^{1}H NMR (500 MHz, DMSO-d_6, 80 °C) δ 7.53 – 7.07 (m, 10H, 1''-4'', 1'''-4''')), 6.80 (s, 1H, HC(7)), 4.96 (t, $^3J_{H\text{-}H}$= 8.3 Hz, 1H, HC(2)), 3.42 – 3.22 (m, 2H, $H_2C(5)$), 2.25 – 2.09 (m, 1H, $H_aC(4)$), 1.93 – 1.78 (m, 2H, $H_aC(3)$, $H_bC(4)$), 1.75 – 1.60 (m, 1H, $H_bC(3)$), 0.98 (s, 9H, $H_3C(1')$), 0.23 (s, 3H, $H_3C(2')$), 0.20 (s, 3H, $H_3C(2')$).

^{13}C NMR (126 MHz, DMSO-d_6, 25 °C) δ 169.5, 143.0, 140.0, 138.9, 136.6, 130.0, 128.2, 128.1, 127.9, 127.5, 126.5, 59.8, 51.2, 33.4, 27.2, 25.3, 17.5, -3.4, -4.0.

HRMS: $C_{25}H_{34}NOSi^+$ $[M+H]^+$ calc. 392.2410, found 392.2397.

Cyclization reaction of *N*-(4-(*tert*-butyldimethylsilyl)hex-5-yn-1-yl)-4-nitrobenzenesulfonamide (7f)

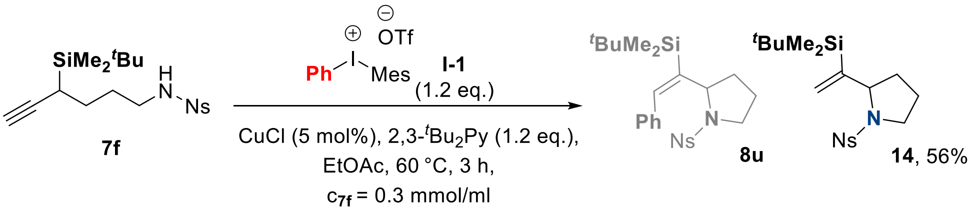

[3] Both (*E*)- and (*Z*)- isomers of **8u** gave a complex NMR spectra due to conformational isomerism. Reaction progress was easiest to monitor by GC-MS, where the two C=C bond isomers could be distinguished by a mass-to-charge ratio (m/z) of 376 (M - CH_3) and 334 (M - ^{t}Bu).

2-(1-(*tert*-Butyldimethylsilyl)vinyl)-1-((4-nitrophenyl)sulfonyl)pyrrolidine (**14**):

Synthesis procedure:

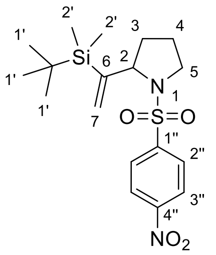

An oven-dried round-bottom flask equipped with a magnetic stirrer was charged with Ar. MesPhIOTf (90 mg, 0.15 mmol, 1.2 equiv), CuCl (1 mg, 6.3 μmol, 5 mol %), *N*-(4-(*tert*-butyldimethylsilyl)hex-5-yn-1-yl)-4-nitrobenzene sulfonamide **7f** [3] (50 mg, 0.13 mmol, 1.0 equiv) was added. The flask was then evacuated and charged with argon using a Schlenk line. Ethyl acetate (5 mL) and 2,6-di-*tert*-butylpyridine (29 mg, 1.5 mmol, 1.2 equiv) were added, and the reaction mixture was heated at 60 °C for 3 h.
The reaction mixture was cooled to room temperature and saturated $NaHCO_3$ aqueous solution (10 mL) and DCM (5 mL) was added. The aqueous layer was extracted with DCM (2 × 5 mL). The organic layers were combined and washed with saturated Na_2CO_3 aqueous solution (2 × 10 mL) and saturated NaCl aqueous solution (10 mL), dried over Na_2SO_4, filtered, and concentrated in vacuo. The crude material was purified by column chromatography on silica gel (DCM/Hex 40%→50%). Product **14** was obtained as a white amorphous solid (51 mg, η = 56%).

Characterization data:

Spectral data matches those in the literature [3].

Synthesis of 6-phenyl-1,2,3,6-tetrahydropyridine 9a–c derivatives

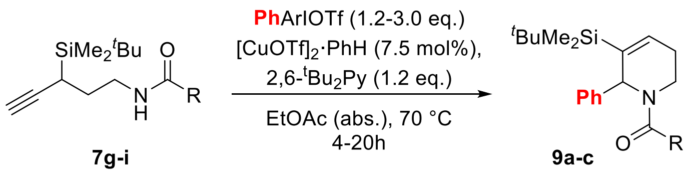

1-(5-(*tert*-Butyldimethylsilyl)-6-phenyl-3,6-dihydropyridin-1(2*H*)-yl)ethan-1-one (**9a**):

Synthesis procedure:

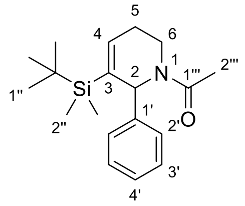

The reaction was performed following a modified **General procedure C** with *N*-(3-(*tert*-butyldimethylsilyl)pent-4-yn-1-yl)acetamide (**7g**,100 mg, 0.42 mmol, 1.0 equiv), PhMesIOTf (237 mg, 0.50 mmol, 1.2 equiv), $[CuOTf]_2$·PhH (16 mg, 0.03 mmol, 7.5 mol %), 2,6-*t*-Bu_2Py (0.11 mL, 0.50 mmol, 1.2 equiv), abs. EtOAc (5 mL). Reaction temperature – 70 °C; reaction time – 4 h.

Column chromatography (0–10% EtOAc/DCM), followed by preparative TLC (50% EtOAc/DCM) afforded the product **9a** (46 mg, 35% yield) as a yellow oil.

The product is observed on NMR as a mixture of rotamers (α and β). Ratio in $CDCl_3$ (20 °C): α:β = 79:21. Only one rotamer is observed in DMSO-d_6 at 80 °C due to coalescence.

Characterization data:

^{1}H NMR (500 MHz, $CDCl_3$) *major rotamer (α):* δ 7.41 (d, $^{3}J_{H\text{-}H}$ = 7.1 Hz, 2H, H-C(2')), 7.37 – 7.22 (m, 3H, H-C(3', 4')), **6.40** (s, 1H, HC(2)), 6.37 – 6.31 (m, 1H, HC(4)), 3.52 (dd, $^{2}J_{H\text{-}H}$ = 14.0 Hz, $^{3}J_{H\text{-}H}$ = 6.6 Hz, 1H, H_aC(6)), 3.22 (td, $^{2}J_{H\text{-}H}$ = 14.0 Hz, $^{3}J_{H\text{-}H}$ = 13.2, 4.7 Hz, 1H, H_bC(6)), 2.52 – 2.39 (m, 1H, H_aC(5)), 2.22 (dt, $^{2}J_{H\text{-}H}$ = 17.9 Hz, $^{3}J_{H\text{-}H}$ = 4.7 Hz, 1H, H_bC(5)), 2.06 (s, 3H, H_3C(2''')), 0.86 (s, 9H, H_3C(1'')), 0.02 (s, 3H, H_3C(2'')), -0.43 (s, 3H, H_3C(2'')); *minor rotamer (β):* δ 7.37 – 7.22 (m, 5H, H-C(2', 3')), 6.40 (br s, 1H, HC(4)), 5.42 (s, 1H, HC(2)), 4.44 (dd, $^{2}J_{H\text{-}H}$ =13.4 Hz, $^{3}J_{H\text{-}H}$ = 6.9 Hz, 1H, H_aC(6)), 2.71 (td, $^{2,3}J_{H\text{-}H}$ = 12.6 Hz, $^{3}J_{H\text{-}H}$ = 5.0 Hz, 1H, H_bC(6)), 2.36 (s, 3H, H_3C(2''')), 2.16 (dt, $^{2}J_{H\text{-}H}$ =18.2 Hz, $^{3}J_{H\text{-}H}$ = 5.0 Hz, 1H, H_aC(5)), 2.12 – 2.04 (m, 1H, H_bC(5)), 0.86 (s, 9H, H_3C(1'')), 0.05 (s, 3H, H_3C(2'')), -0.41 (s, 3H, H_3C(2'')).

^{1}H NMR (500 MHz, DMSO-d_6, 80 °C) δ 7.45 – 7.21 (m, 5H, H-C(2', 3')), 6.40 (br s, 1H, HC(4)), 6.28 (br s, 1H, HC(2)), 3.61 (br s, 1H, H_aC(6)), 3.10 (br s, 1H, H_bC(6)), 2.43 (br s, 1H, H_aC(5)), 2.23 (dt, $^{2}J_{H\text{-}H}$ = 18.1 Hz, $^{3}J_{H\text{-}H}$ = 3.7 Hz, 1H, H_bC(5)), 1.99 (m, 3H, H_3C(2''')), 0.85 (s, 9H, H_3C(1'')), 0.02 (s, 3H, H_3C(2'')), -0.40 (s, 3H, H_3C(2'')).

^{13}C NMR (126 MHz, $CDCl_3$) *major rotamer (α):* δ 167.9, 141.0, 137.6, 136.6, 129.5, 128.1, 127.5, 54.3, 38.2, 27.4, 26.9, 21.8, 17.5, -5.7, -6.1; *minor rotamer (β):* δ 167.8, 140.1, 139.0, 135.9, 128.8, 128.4, 128.0, 60.2, 33.0, 26.8, 26.5, 22.1, 17.6, -5.7, -6.2.

HRMS: $C_{19}H_{30}NOSi^+$ $[M+H]^+$ 316.2097, found 316.2090.

(5-(*tert*-Butyldimethylsilyl)-6-phenyl-3,6-dihydropyridin-1(2*H*)-yl)(phenyl)methanone (**9b**):

Synthesis procedure:

The reaction was performed following a modified **General procedure C** with *N*-(3-(*tert*-butyldimethylsilyl)pent-4-yn-1-yl)benzamide (**7h**, 100 mg, 0.33 mmol, 1.0 equiv), PhMesIOTf (188 mg, 0.40 mmol, 1.2 equiv), $[CuOTf]_2$·PhH (13 mg, 0.02 mmol, 7.5 mol %), 2,6-*t*-Bu_2Py (86 µl, 0.40 mmol, 1.2 equiv), abs. EtOAc (5 mL). Reaction temperature – 70 °C; reaction time – 4 h. Column chromatography (90–100% DCM/Hex) afforded the product **9b** (77 mg, 76% purity by qNMR, 47% yield) as a yellowish oil. Further purification by preparative HPLC afforded the pure product **9b** as a colorless oil (39 mg of impure sample afforded 5 mg of clean product).

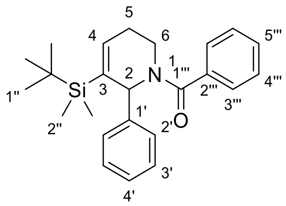

The product is observed on NMR as a mixture of rotamers (α and β). Ratio in $CDCl_3$ (25 °C): α:β = 74:26. Only one rotamer is observed in DMSO-d_6 at 80 °C due to coalescence.

Characterization data:

^{1}H NMR (500 MHz, $CDCl_3$) *major rotamer (α):* δ 7.52 (d, $^{3}J_{H\text{-}H}$ = 7.4 Hz, 2H, H-C(2')), 7.40 – 7.27 (m, 8H, H-C(3', 4', 3''', 4''', 5''')), 6.49 (s, 1H, HC(2)), 6.39 (br s, 1H, HC(4)), 3.44 (dd, $^{2}J_{H\text{-}H}$ = 13.5 Hz, $^{3}J_{H\text{-}H}$ = 6.9 Hz, 1H, H_aC(6)), 3.16 (td, $^{2,3}J_{H\text{-}H}$ = 13.5 Hz, $^{3}J_{H\text{-}H}$ = 4.3 Hz, 1H, H_bC(6)), 2.50 – 2.38 (m, 1H, H_aC(5)), 2.15 (dt, $^{2}J_{H\text{-}H}$ = 18.1 Hz, $^{3}J_{H\text{-}H}$ = 4.3 Hz, 1H, H_bC(5)), 0.91 (s, 9H, H_3C(1'')), 0.05 (s, 3H, H_3C(2'')), -0.37 (s, 3H, H_3C(2'')); *minor rotamer (β):* δ 7.47 (br s, 4H, H-C(3''', 4''')), 7.40 – 7.27 (m, 4H, H-C(2', 4', 5''')), 7.06 (br s, 2H, H-

C(3'), 6.44 (br s, 1H, HC(4)), 5.41 (s, 1H, HC(2)), 4.56 (dd, $^{2}J_{H\text{-}H}$ = 12.9 Hz, $^{3}J_{H\text{-}H}$ = 7.2 Hz, 1H, $H_aC(6)$), 2.99 (td, $^{2,3}J_{H\text{-}H}$ = 12.9 Hz, $^{3}J_{H\text{-}H}$ = 4.6 Hz, 1H, $H_bC(6)$), 2.71 – 2.59 (m, 1H, $H_aC(5)$), 2.37 – 2.25 (m, 1H, $H_bC(5)$), 0.76 (s, 9H, $H_3C(1'')$), -0.09 (s, 3H, $H_3C(2'')$), -0.62 (s, 3H, $H_3C(2'')$).

^{1}H NMR (500 MHz, DMSO-d_6, 80 °C) δ 7.77 – 6.99 (m, 10H, H-C(1'-4', 3'''-5''')), 6.45 (dd, $^{3}J_{H\text{-}H}$ = 4.8, 2.3 Hz, 1H, HC(4)), 6.29 (br s, 1H, HC(2)), 3.47 (br s, 2H, $H_2C(6)$), 2.48 – 2.37 (m, 1H, $H_aC(5)$), 2.32 – 2.15 (m, 1H, $H_bC(5)$), 0.86 (s, 9H, $H_3C(1'')$), 0.02 (s, 3H, $H_3C(2'')$), -0.40 (s, 3H, $H_3C(2'')$).

^{13}C NMR (126 MHz, DMSO-d_6, 25 °C) δ 169.1, 168.3, 140.6, 139.5, 138.7, 137.8, 136.7, 136.3, 135.5, 135.1, 129.7, 129.4, 129.1, 128.7, 128.5, 128.4, 128.4, 128.1, 127.8, 126.8, 126.3, 126.3, 60.2, 54.2, 38.6, 33.0, 27.0, 26.7, 26.7, 26.1, 17.1, 16.8, -5.7, -5.9, -6.0, -6.0.

HRMS: $C_{24}H_{32}NOSi^{+}$ $[M+H]^{+}$ calc. 378.2253, found 378.2248.

(5-(*tert*-Butyldimethylsilyl)-6-phenyl-3,6-dihydropyridin-1(2*H*)-yl)(3,5-dinitrophenyl)methanone (**9c**)

Synthesis procedure:

The reaction was performed following a modified **General procedure C** with *N*-(3-(*tert*-butyldimethylsilyl)pent-4-yn-1-yl)-3,5-dinitrobenzamide (**7i**, 138 mg, 0.35 mmol, 1.0 equiv), Ph_2IOTf (456 mg, 1.06 mmol, 3.0 equiv), $[CuOTf]_2{\cdot}PhH$ (13 mg, 0.03 mmol, 7.5 mol %), 2,6-*t*-Bu_2Py (90 µl, 0.42 mmol, 1.2 equiv), abs. EtOAc (5 mL). Reaction temperature – 70 °C; reaction time – 20 h. Column chromatography (80–100% DCM/Hex) afforded the product **9c** (31 mg, 19% yield) as a yellow solid.

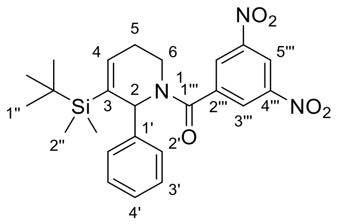

The product is observed on NMR as a mixture of rotamers (α and β). Ratio in MeCN-d_3 (25 °C): α:β = 84:16. Only one rotamer is observed in MeCN-d_3 at 60 °C due to coalescence.

Characterization data:

^{1}H NMR (500 MHz, CD_3CN, 25 °C) _major rotamer (α):_ δ 8.91 (s, 1H, H-C(5''')), 8.42 (s, 2H, H-C(3''')), 7.51 (d, $^{3}J_{H\text{-}H}$ = 7.2 Hz, 2H, H-C(2')), 7.40 (t, $^{3}J_{H\text{-}H}$ = 7.2 Hz, 2H, H-C(3')), 7.35 (t, $^{3}J_{H\text{-}H}$ = 7.2 Hz, 1H, H-C(4')), 6.47 (br s, 1H, HC(4)), 6.41 (s, 1H, HC(2)), 3.28 (dd, $^{2}J_{H\text{-}H}$ = 14.2 Hz, $^{3}J_{H\text{-}H}$ = 7.1 Hz, 1H, $H_aC(6)$), 3.21 (td, $^{2}J_{H\text{-}H}$ = 14.2 Hz, $^{3}J_{H\text{-}H}$ = 13.0, 4.7 Hz, 1H, $H_bC(6)$), 2.55 – 2.41 (m, 1H, $H_aC(5)$), 2.26 – 2.10 (m, 1H, $H_bC(5)$)[4], 0.93 (s, 9H, $H_3C(1'')$), 0.09 (s, 3H, $H_3C(2'')$), -0.40 (s, 3H, $H_3C(2'')$); _minor rotamer (β):_ δ 9.02 (s, 1H, H-C(5''')), 8.60 (s, 2H, H-C(3''')), 7.55 – 7.10 (m, 5H, H-C(2'-4')), 6.52 (br s, 1H, HC(4)), 5.14 (s, 1H, HC(2)), 4.50 – 4.32 (m, 1H, $H_aC(6)$), 3.13 – 2.96 (m, 1H, $H_bC(6)$), 2.72 – 2.54 (m, 1H, $H_aC(5)$), 2.41 – 2.28 (m, 1H, $H_bC(5)$), 0.72 (s, 9H, $H_3C(1'')$), -0.09 (s, 3H, $H_3C(2'')$), -0.64 (s, 3H, $H_3C(2'')$).

^{1}H NMR (500 MHz, CD_3CN, 60 °C) δ 8.93 (s, 1H, H-C(5''')), 8.43 (s, 2H, H-C(3''')), 7.57 – 7.17 (m, 5H, H-C(2'-4')), 6.50 (s, 1H, HC(2)), 6.43 (br s, 1H, HC(4)), 3.53 – 3.06 (m, 2H, $H_2C(6)$), 2.71 – 2.37 (m, 1H, $H_aC(5)$), 2.36 – 2.12 (m, 1H, $H_bC(5)$), 0.93 (s, 9H, $H_3C(1'')$), 0.08 (s, 3H, $H_3C(2'')$), -0.36 (s, 3H, $H_3C(2'')$).

[4] Determined by HSQC

^{13}C NMR (126 MHz, CD_3CN, 25 °C) δ 165.6, 149.7, 141.3, 140.7, 138.7, 136.5, 130.5, 129.4, 129.0, 128.0, 120.1, 56.3, 40.1, 28.1, 27.1, 18.1, -5.6, -5.8.

HRMS: $C_{24}H_{30}N_3O_5Si^+$ $[M+H]^+$ calc. 468.1955, found 468.1946.

Melting temperature: T_m = 179 °C

^{1}H and ^{13}C NMR spectra

(5-Bromopent-1-yn-3-yl)(*tert*-butyl)dimethylsilane (**S2**)

^{1}H NMR (500 MHz, CDCl$_3$):

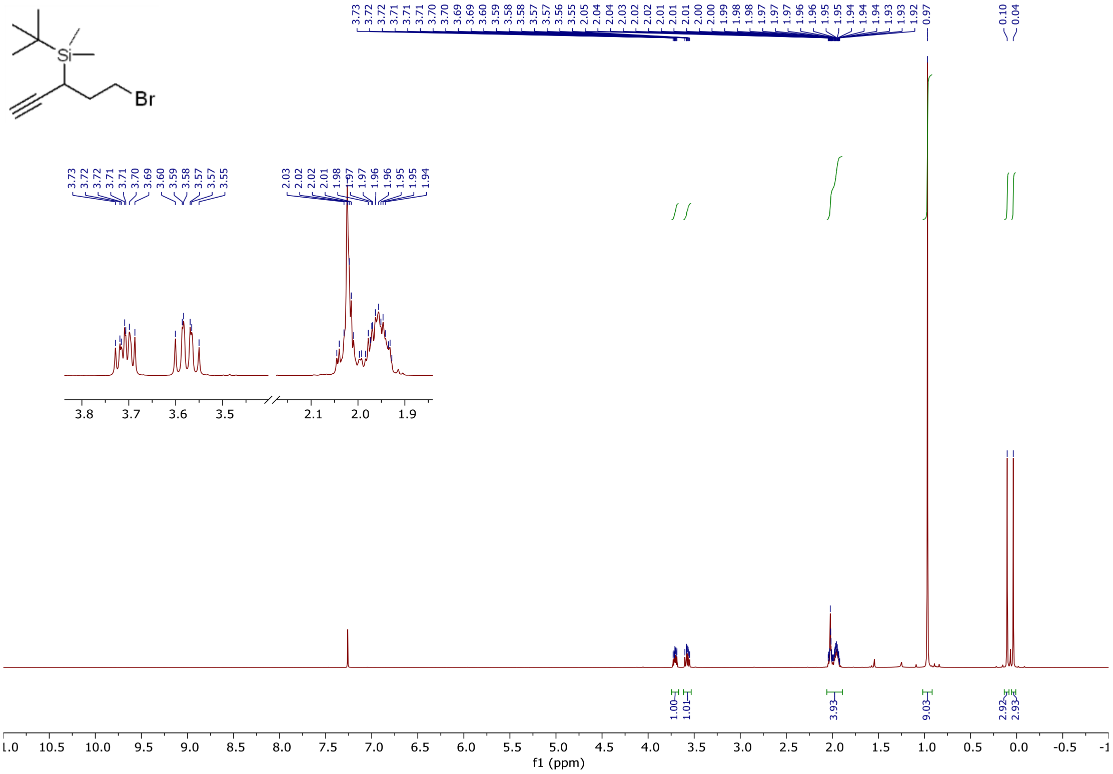

^{13}C {^{1}H} NMR (126 MHz, CDCl$_3$):

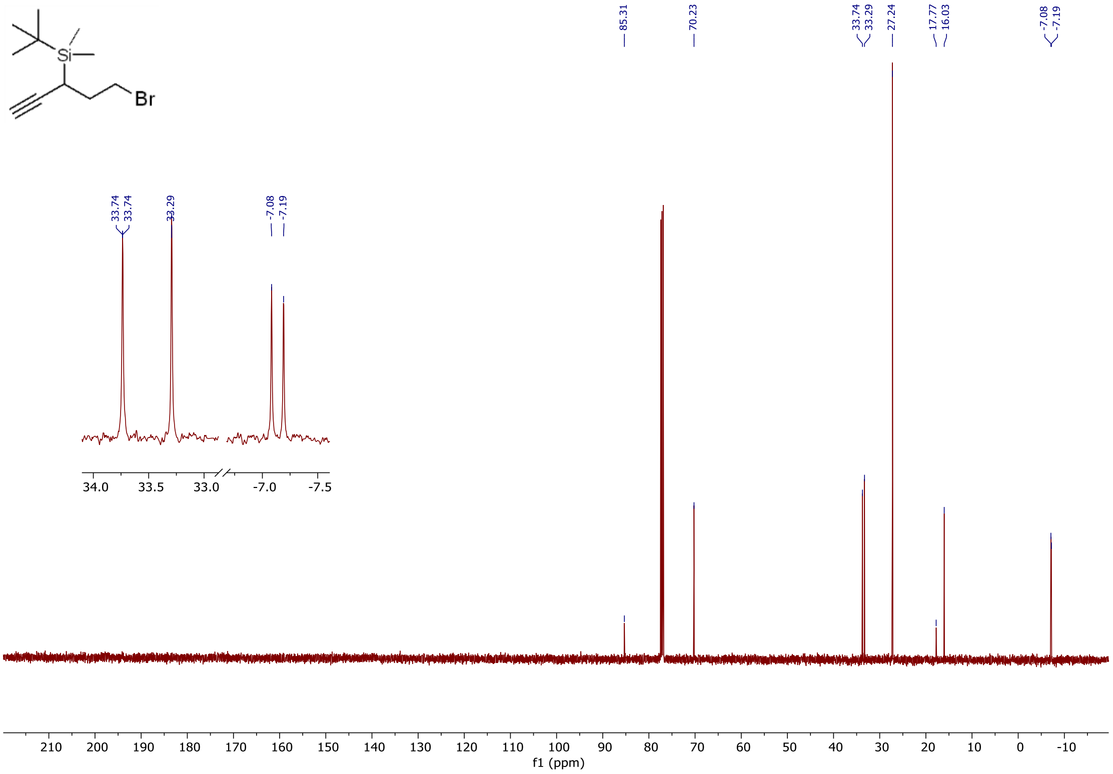

(5-Azidopent-1-yn-3-yl)(*tert*-butyl)dimethylsilane (**S3**)

^{1}H NMR (500 MHz, $CDCl_3$):

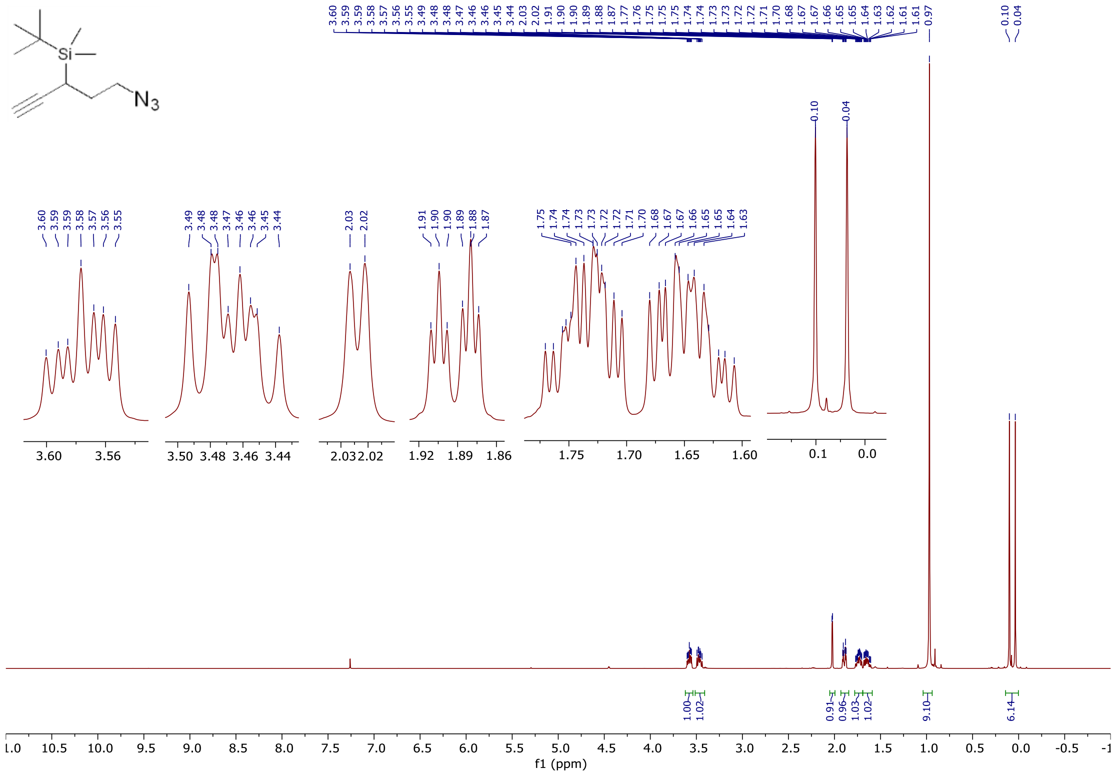

^{13}C {^{1}H} NMR (126 MHz, $CDCl_3$):

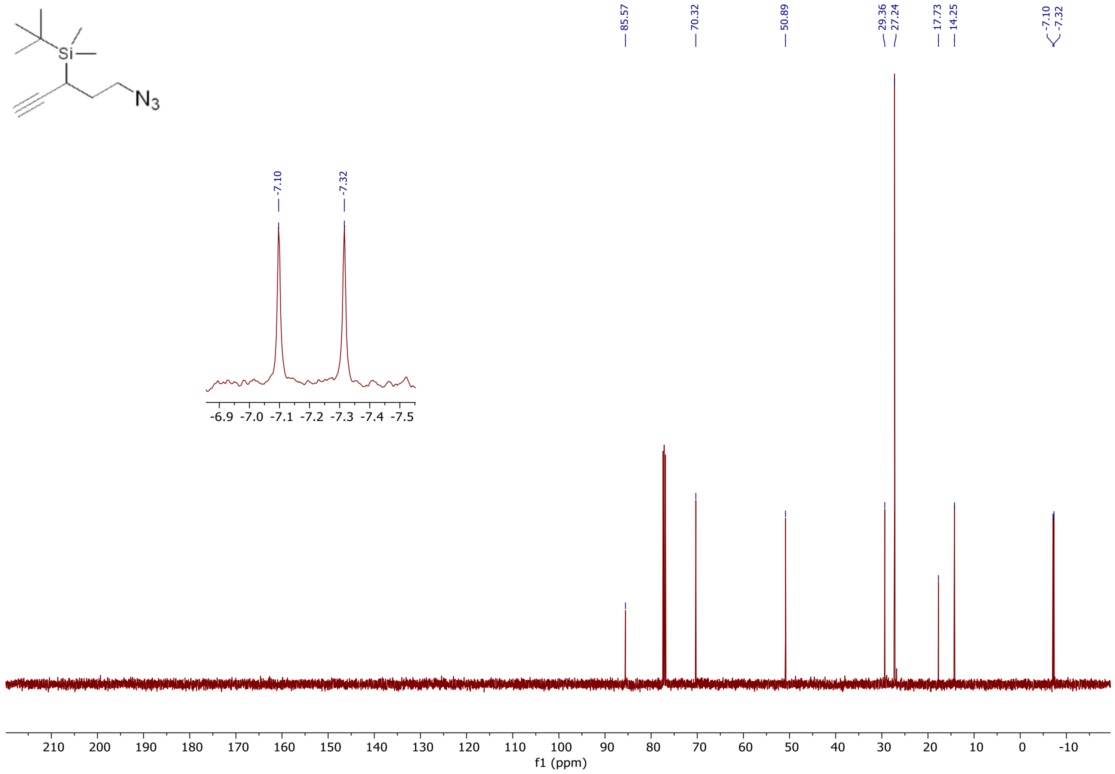

N-(3-(*tert*-Butyldimethylsilyl)pent-4-yn-1-yl)acetamide (**16a**)

^{1}H NMR (500 MHz, $CDCl_3$):

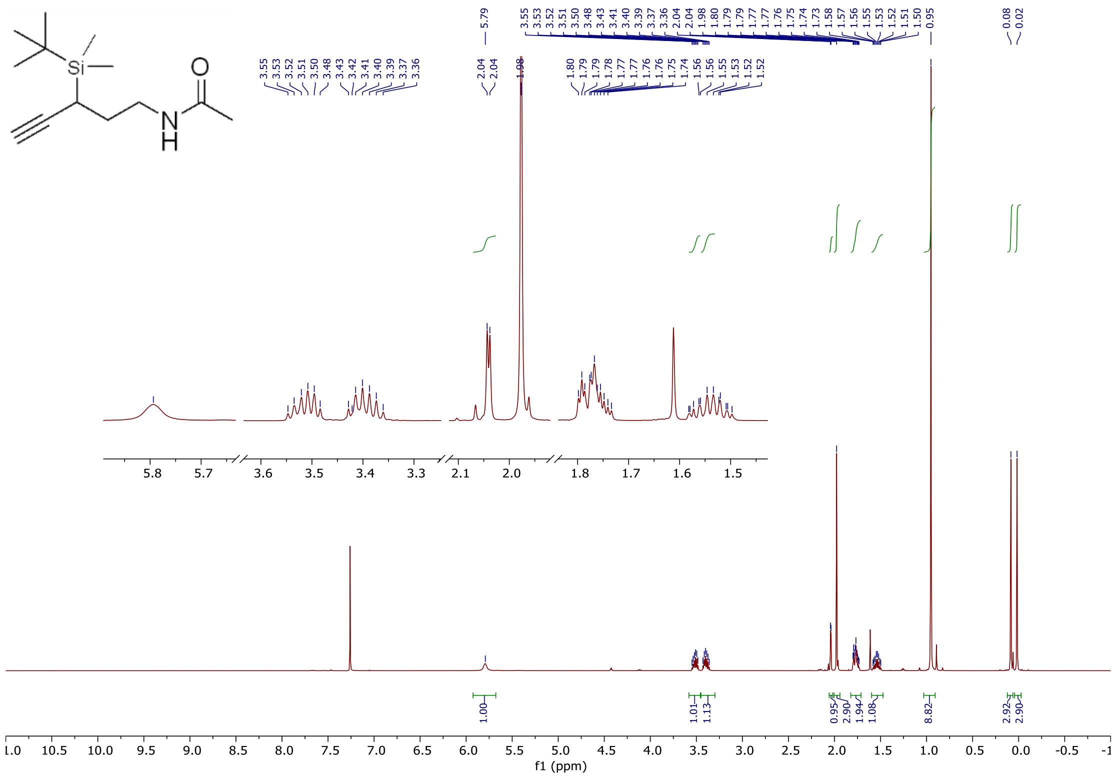

^{13}C {^{1}H} NMR (126 MHz, $CDCl_3$):

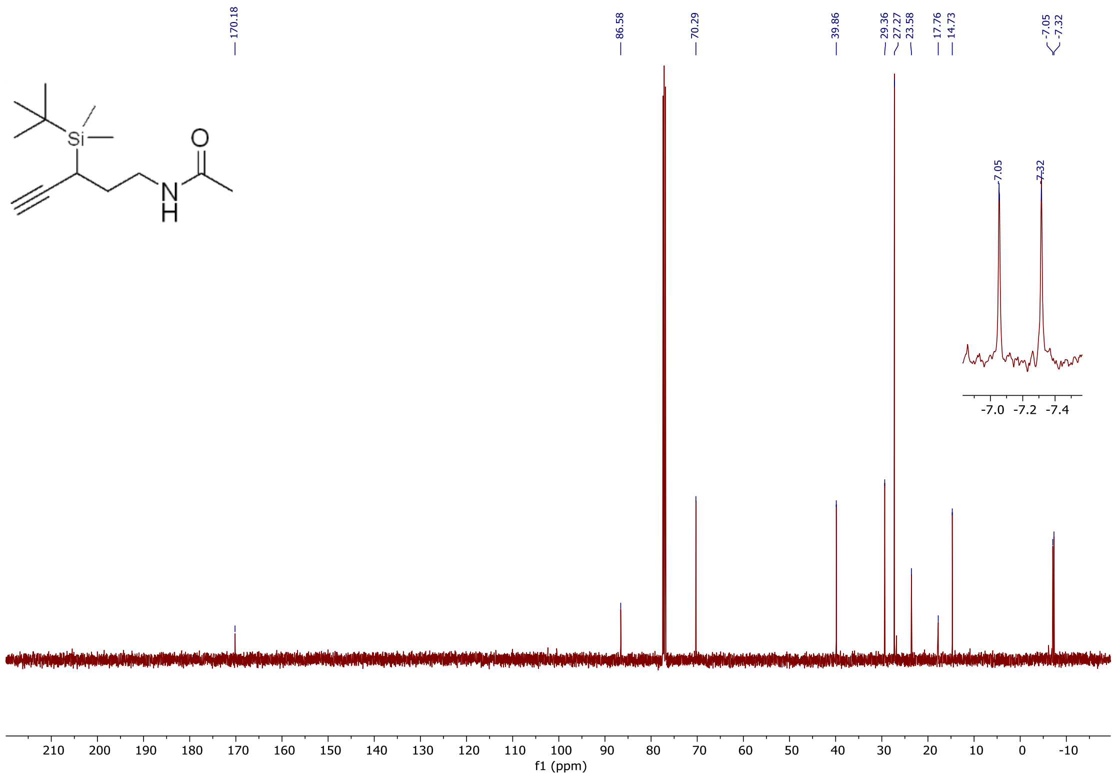

N-(3-(*tert*-Butyldimethylsilyl)pent-4-yn-1-yl)benzamide (**16b**)

^{1}H NMR (500 MHz, $CDCl_3$):

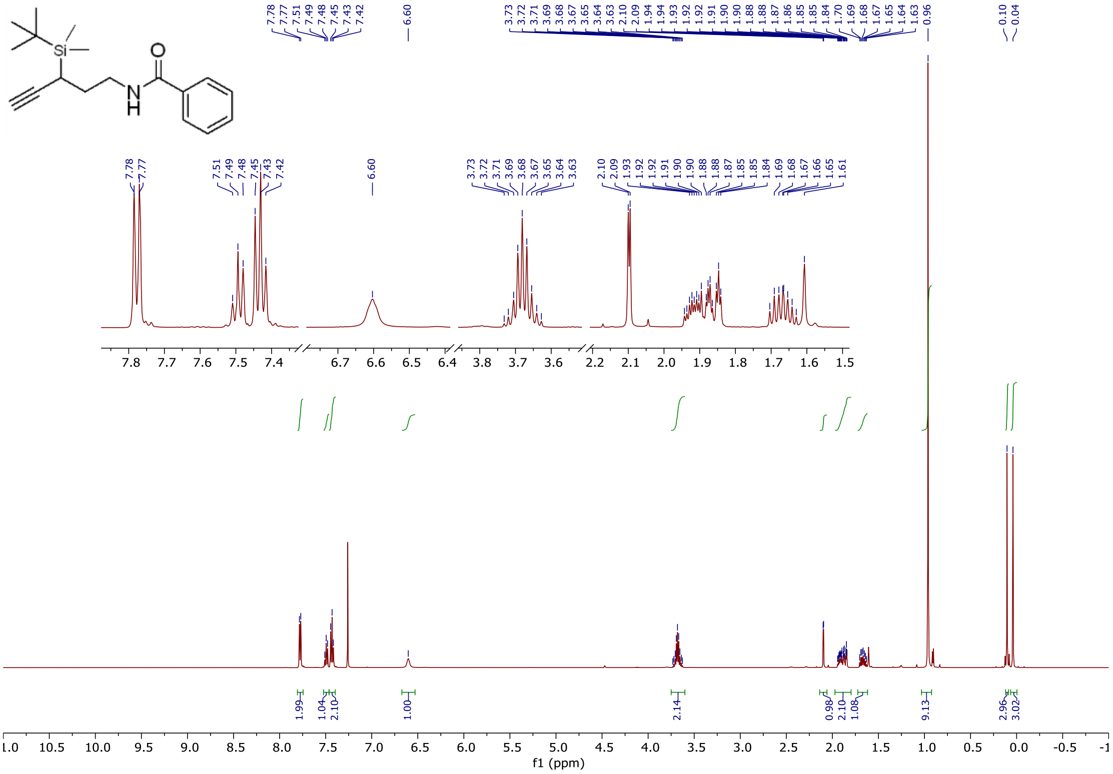

^{13}C {^{1}H} NMR (126 MHz, $CDCl_3$):

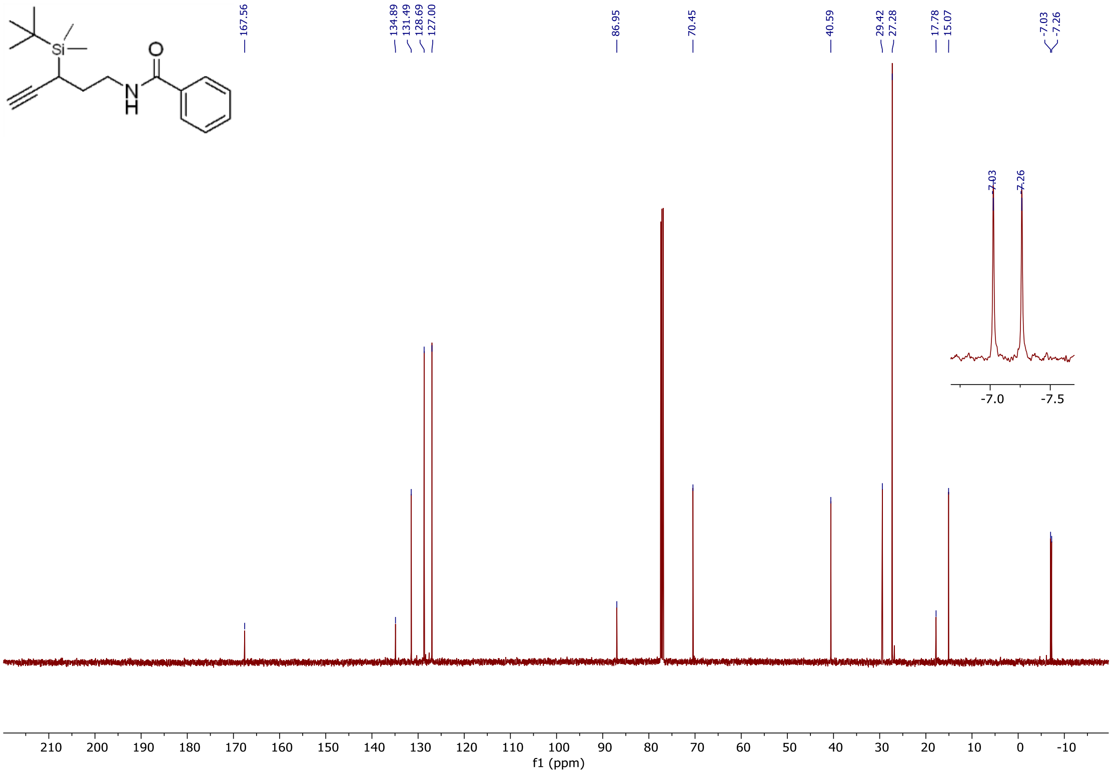

N-(3-(*tert*-butyldimethylsilyl)pent-4-yn-1-yl)-3,5-dinitrobenzamide (**16c**)

^{1}H NMR (500 MHz, $CDCl_3$):

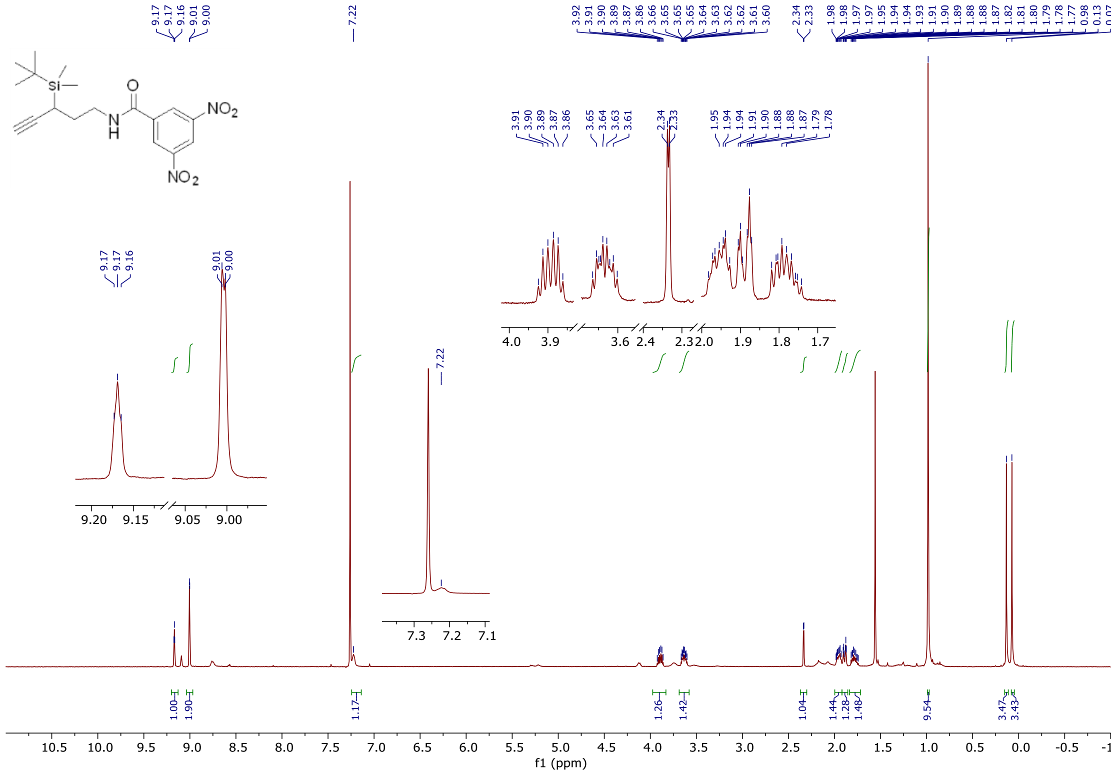

^{13}C {^{1}H} NMR (126 MHz, $CDCl_3$):

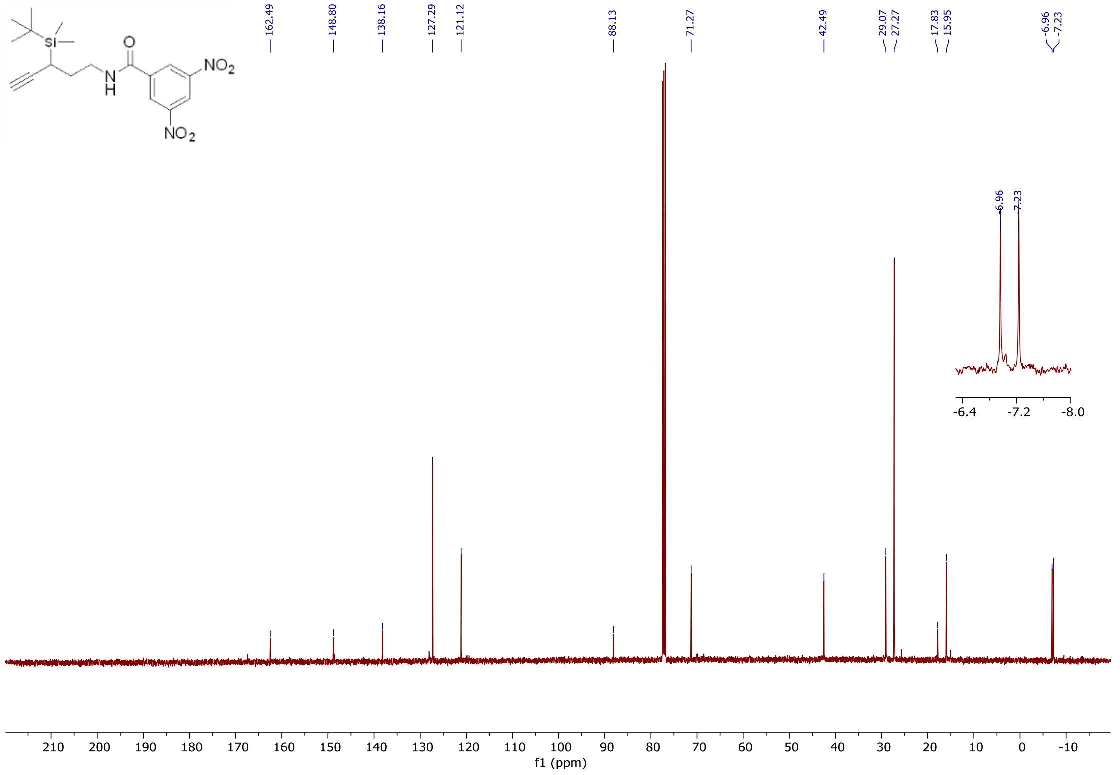

tert-Butyl 4-(*tert*-butyldimethylsilyl)hex-5-ynoate (**7e**)

^{1}H NMR (500 MHz, CDCl$_3$):

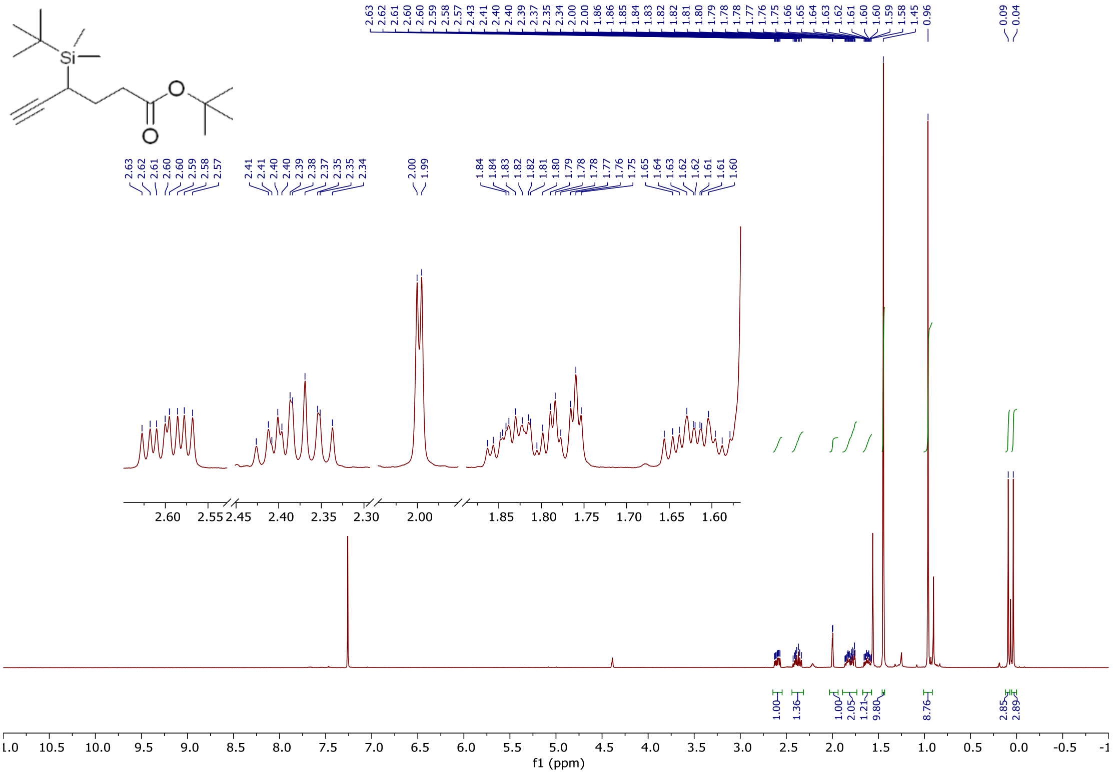

^{13}C {^{1}H} NMR (126 MHz, CDCl$_3$)

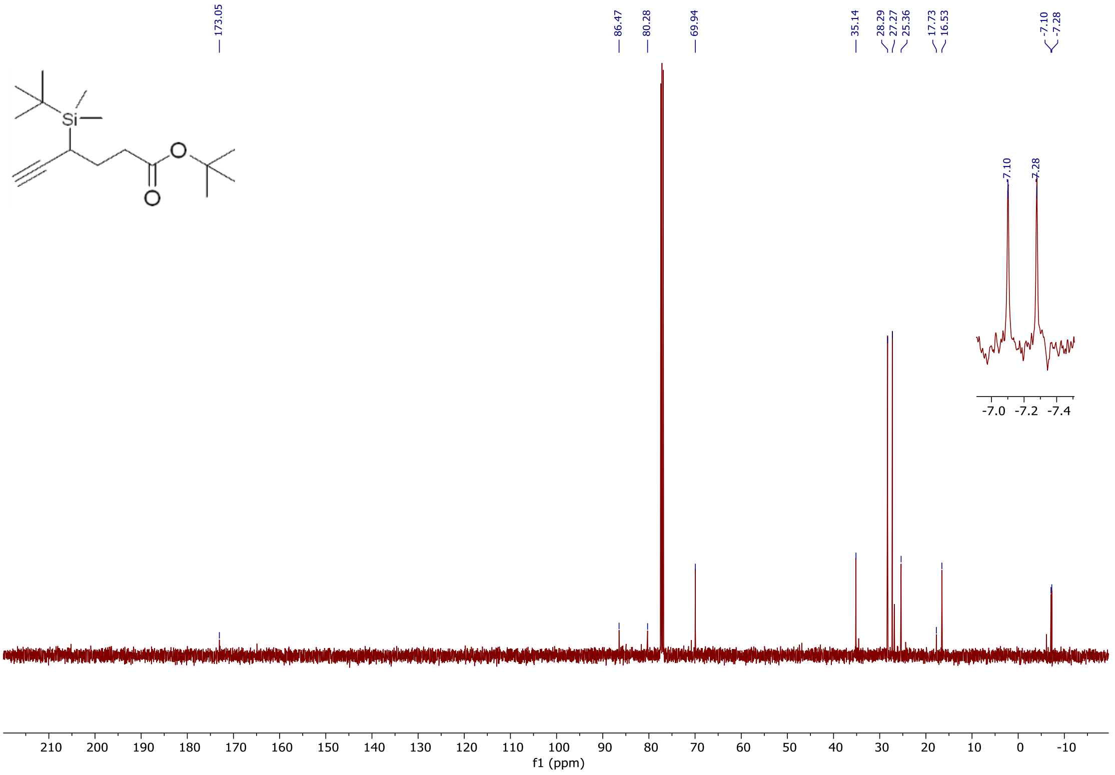

N-(4-(*tert*-Butyldimethylsilyl)hex-5-yn-1-yl)-4-nitro-*N*-phenylbenzenesulfonamide (**S7**)

^{1}H NMR (500 MHz, $CDCl_3$):

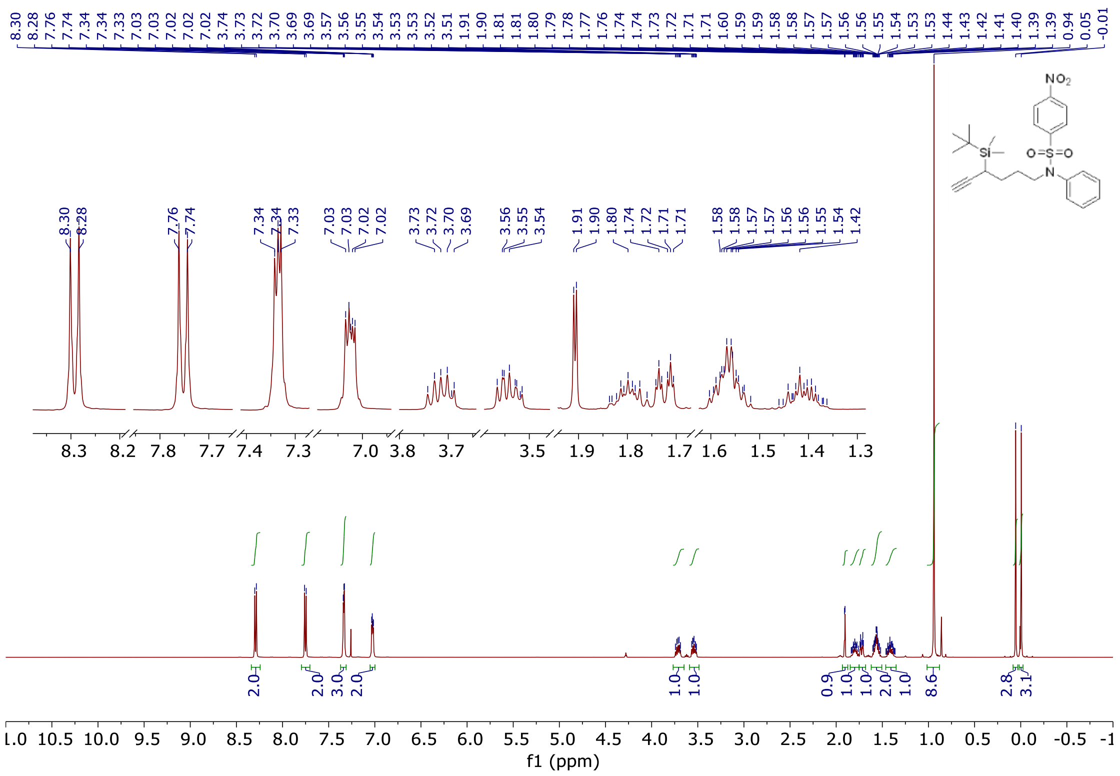

^{13}C {^{1}H} NMR (126 MHz, $CDCl_3$):

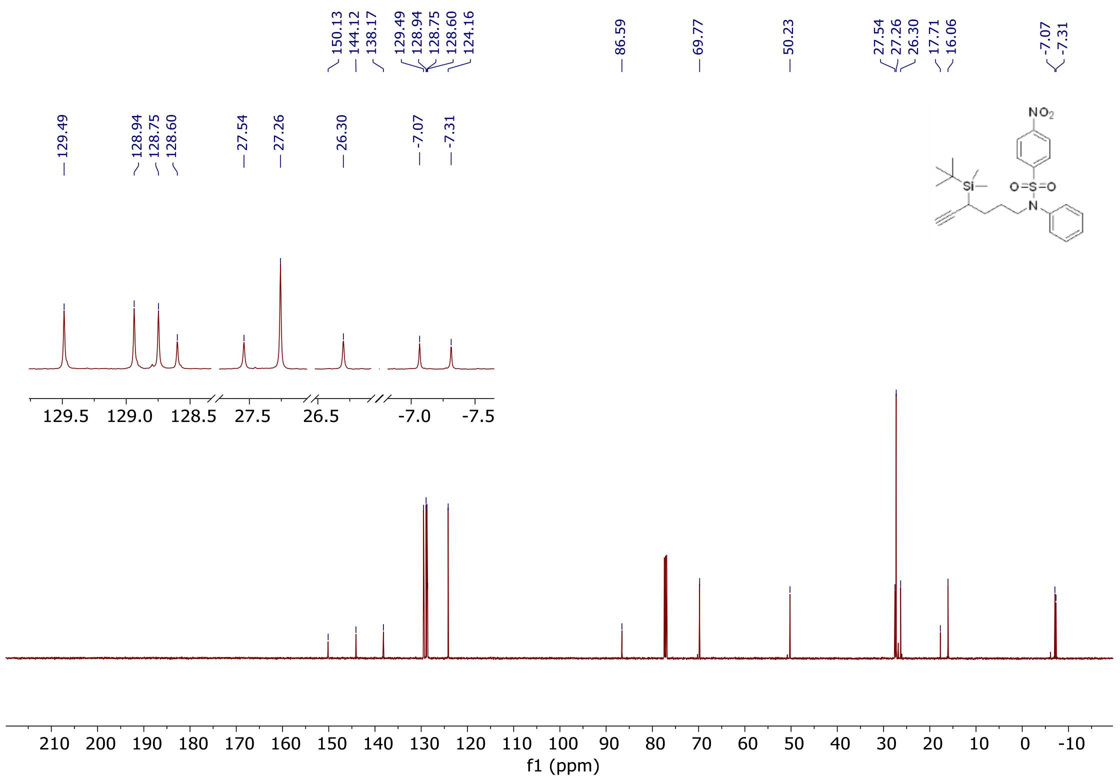

N-(4-(*tert*-Butyldimethylsilyl)hex-5-yn-1-yl)aniline (**7h**)

^{1}H NMR (500 MHz, $CDCl_3$):

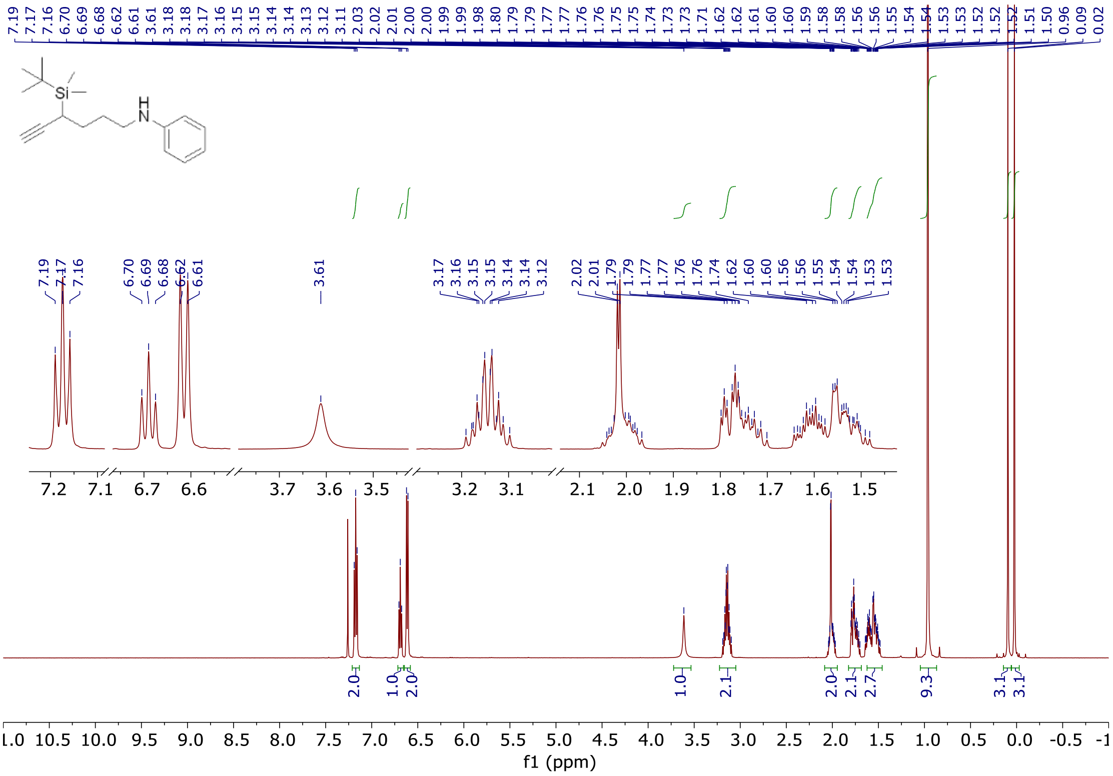

^{13}C {^{1}H} NMR (126 MHz, $CDCl_3$):

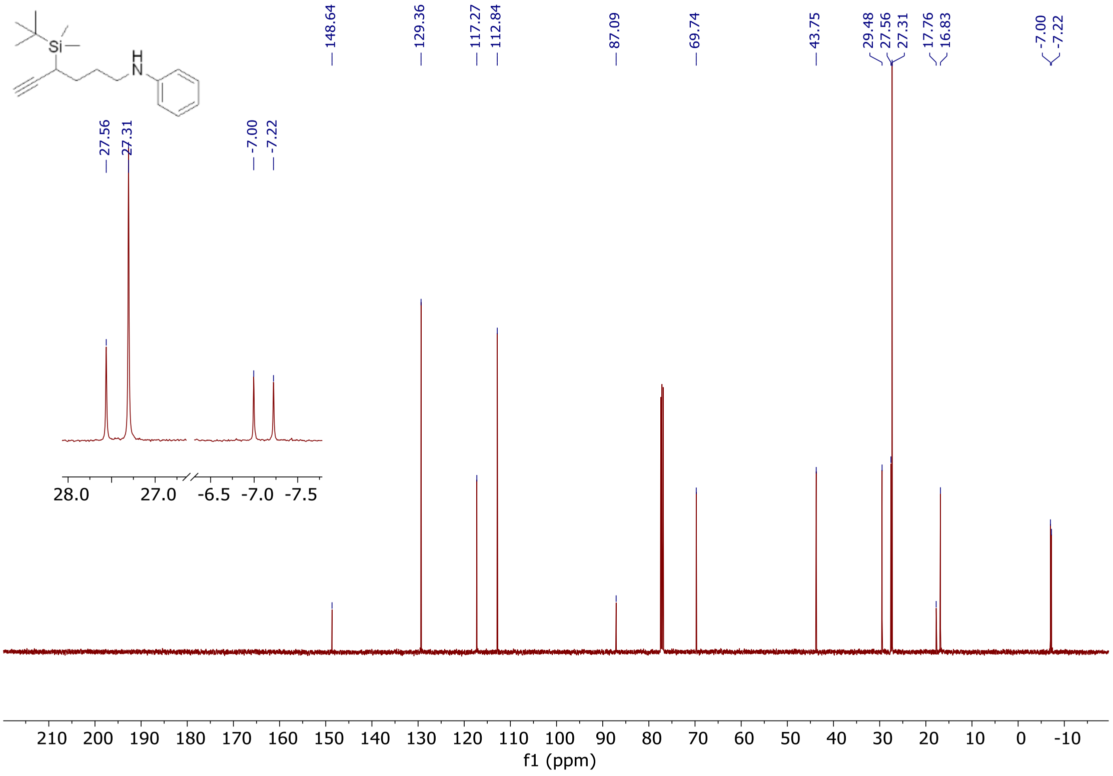

tert-Butyldimethyl((1*E*,3*E*)-1-phenylhepta-1,3-dien-2-yl)silane (**10a**)

^{1}H NMR (500 MHz, $CDCl_3$):

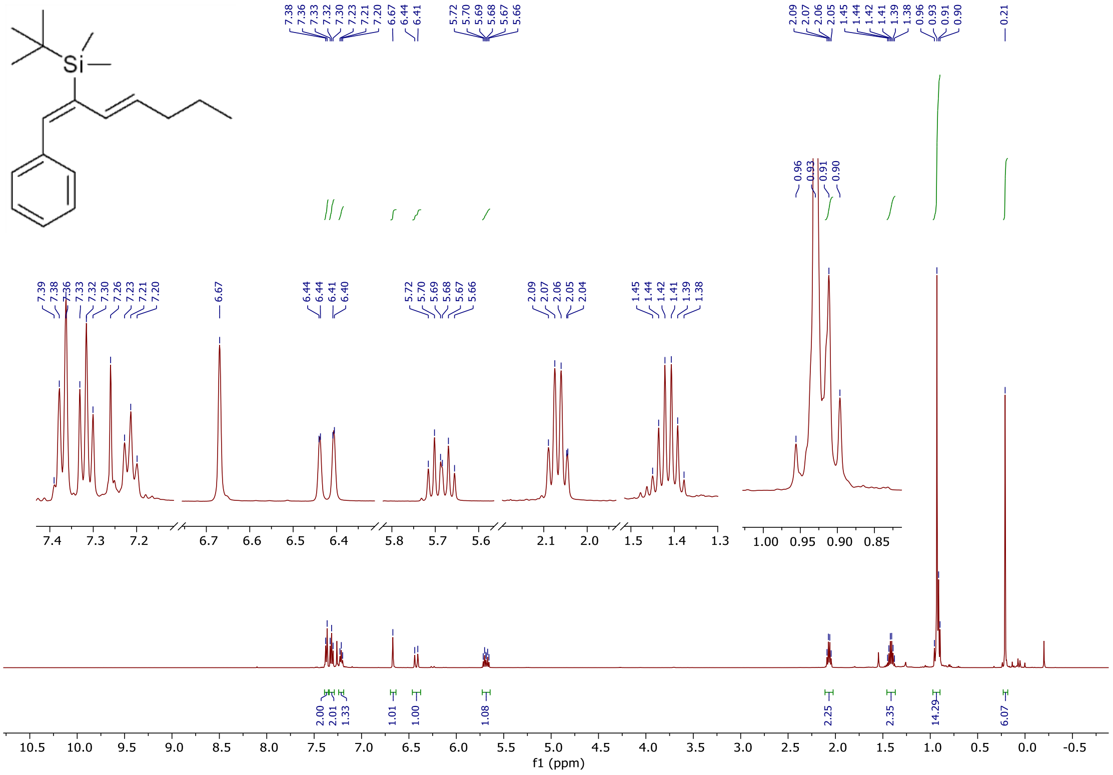

^{13}C {^{1}H} NMR (126 MHz, $CDCl_3$):

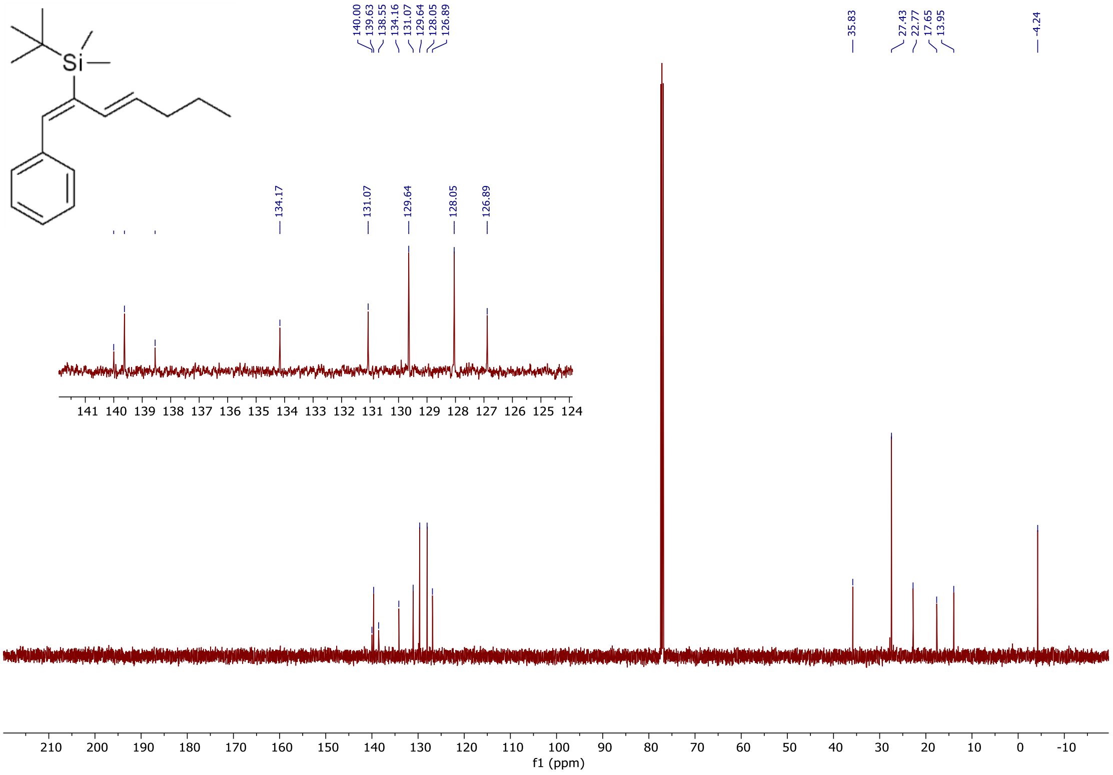

^1H-^1H-NOESY NMR ($CDCl_3$):

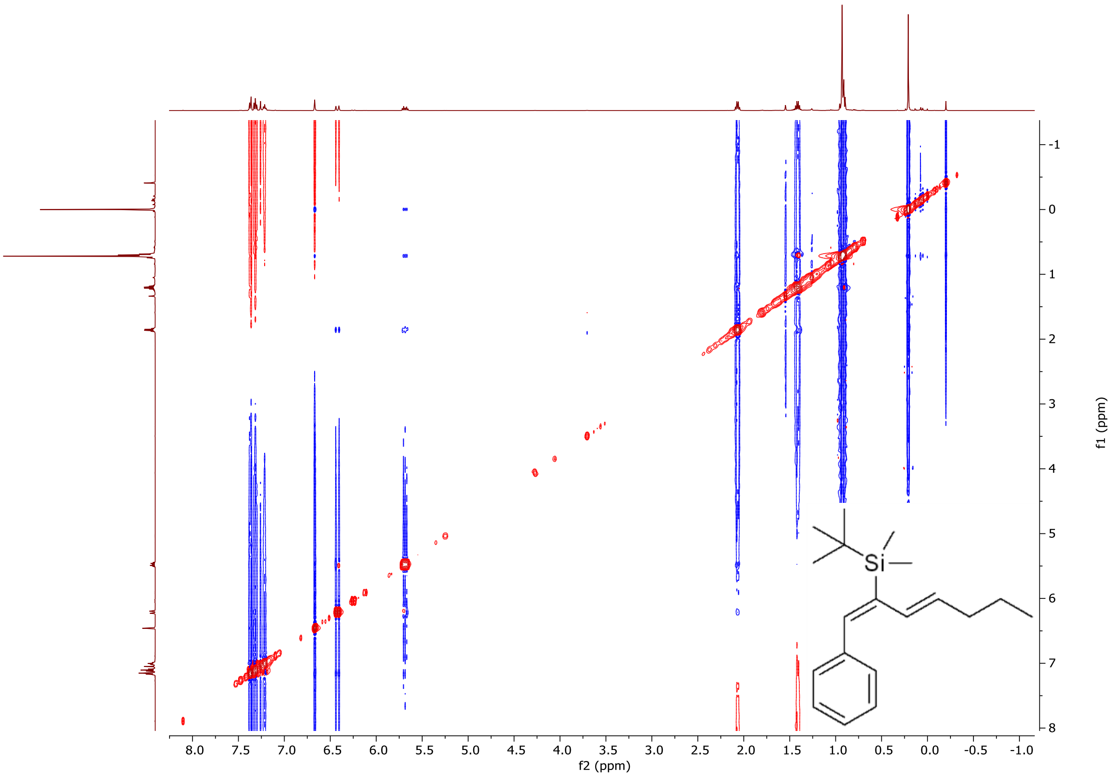

Triisopropyl(1-phenylhepta-1,3-dien-2-yl)silane (**10b**)

^{1}H NMR (500 MHz, $CDCl_3$):

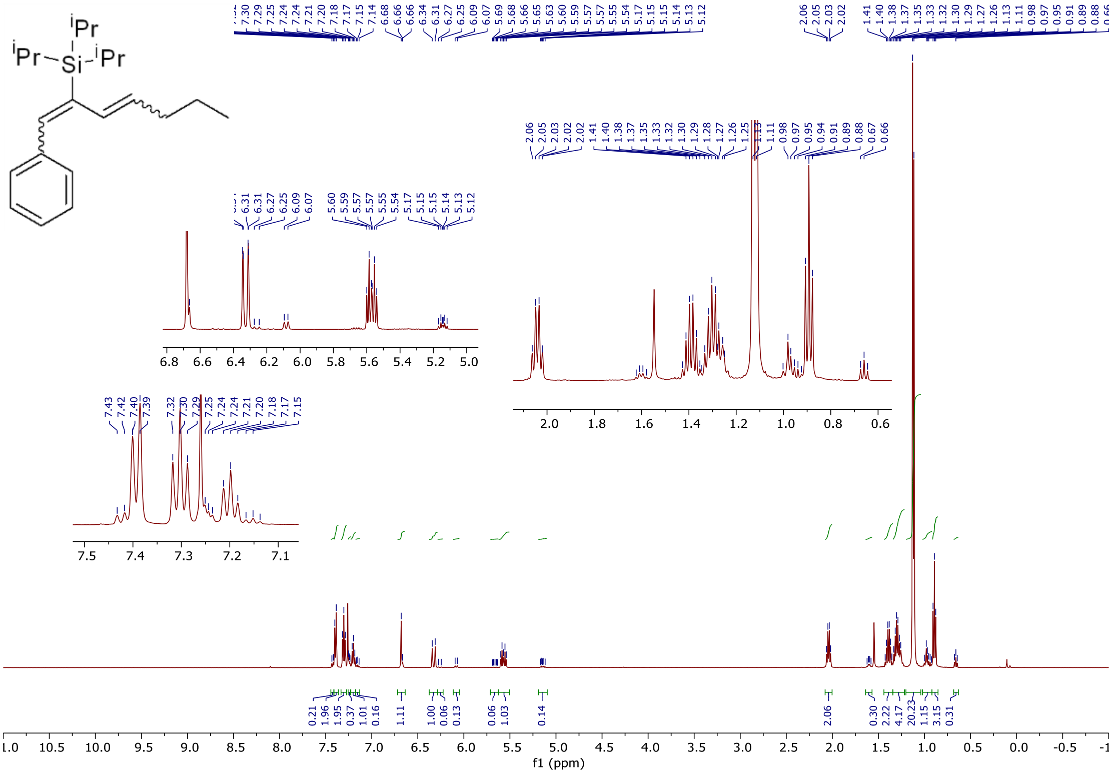

^{13}C {^{1}H} NMR (126 MHz, $CDCl_3$):

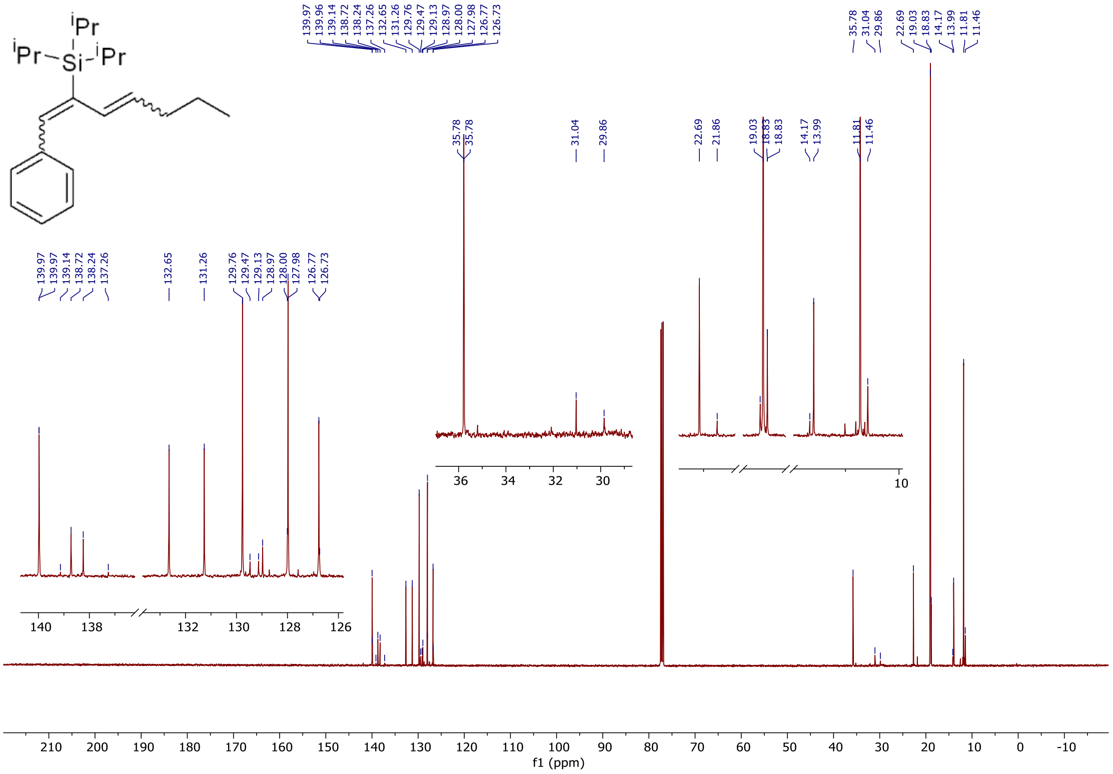

^{1}H-^{13}C-HSQC NMR (CDCl$_3$):

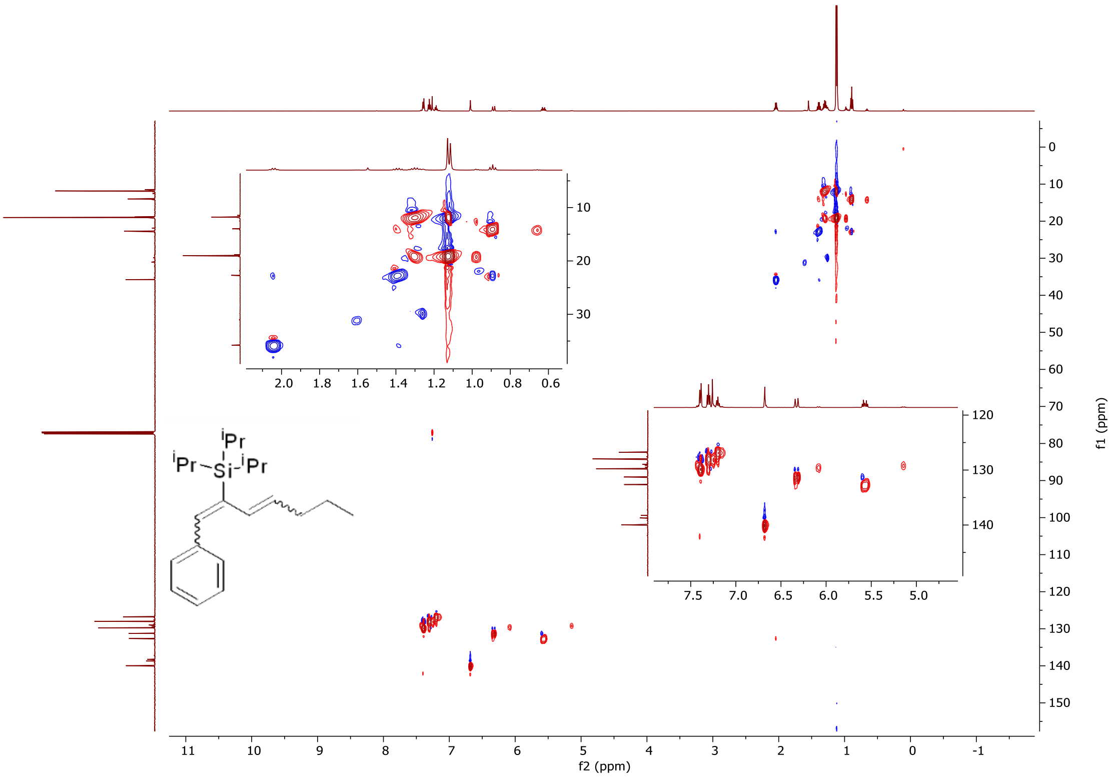

^{1}H-^{13}C-HMBC NMR (CDCl$_3$, 25 °C)

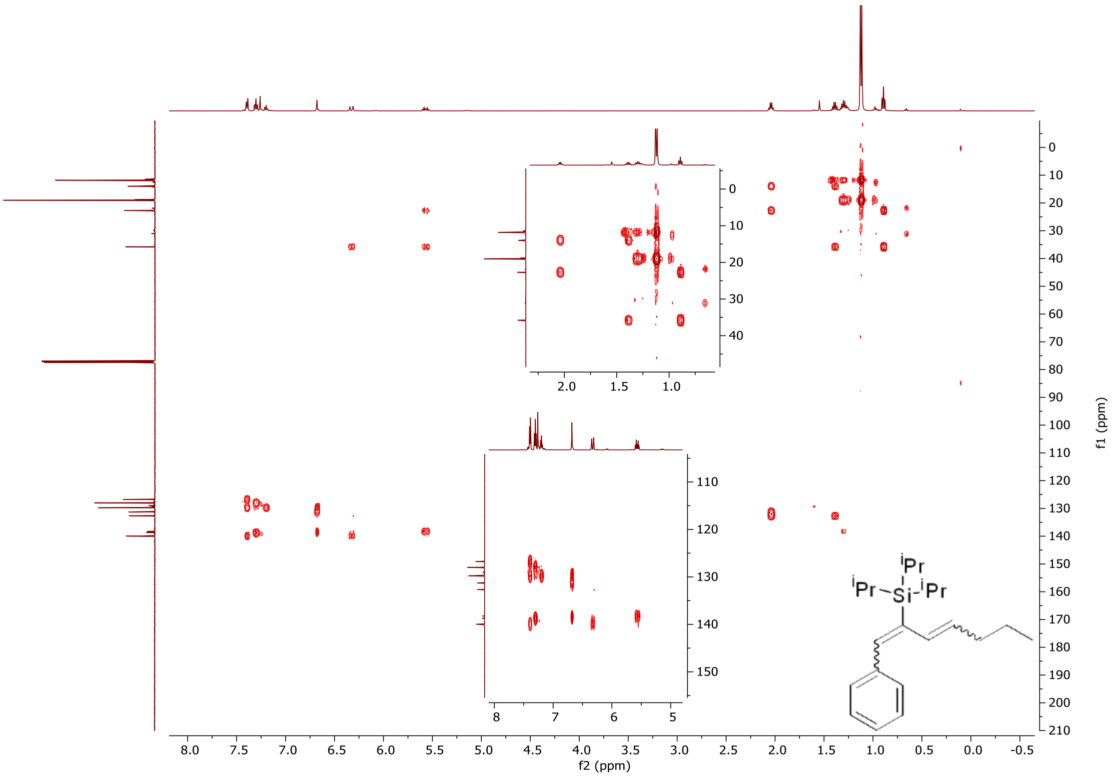

^{1}H-^{1}H-NOESY NMR ($CDCl_3$, 25 °C)

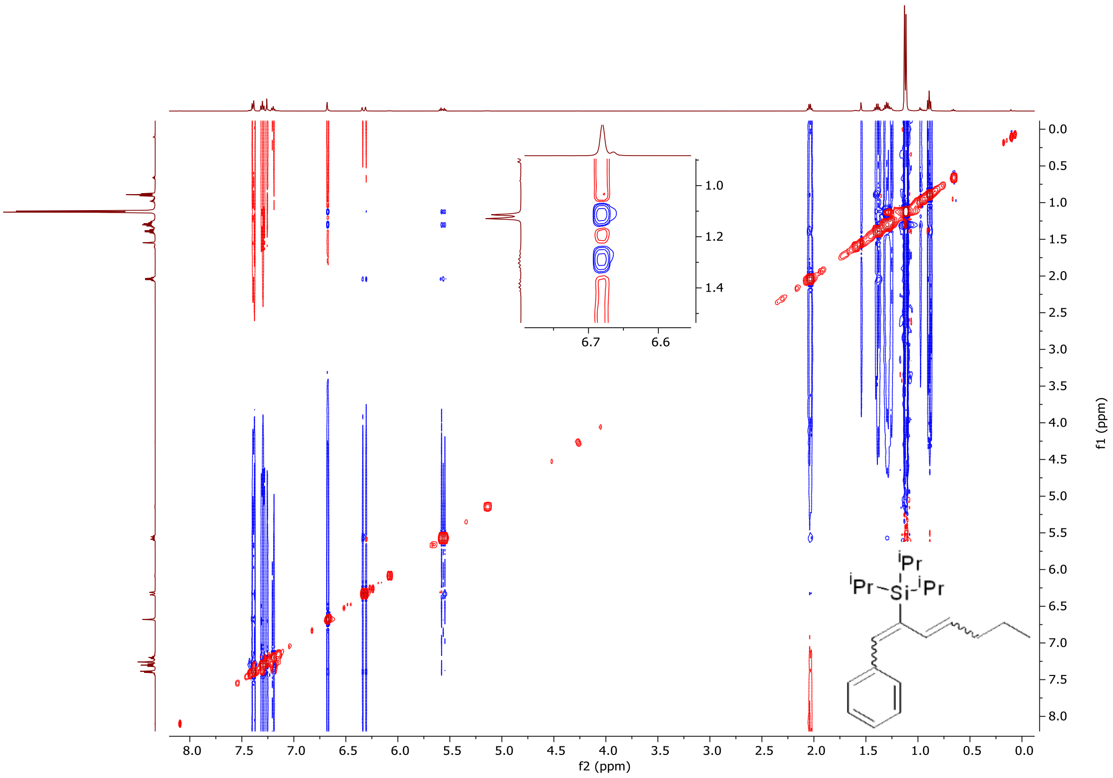

tert-Butyldimethyl((1*E*,3*E*)-1-(*p*-tolyl)hepta-1,3-dien-2-yl)silane (**10c**)

^{1}H NMR (500 MHz, $CDCl_3$):

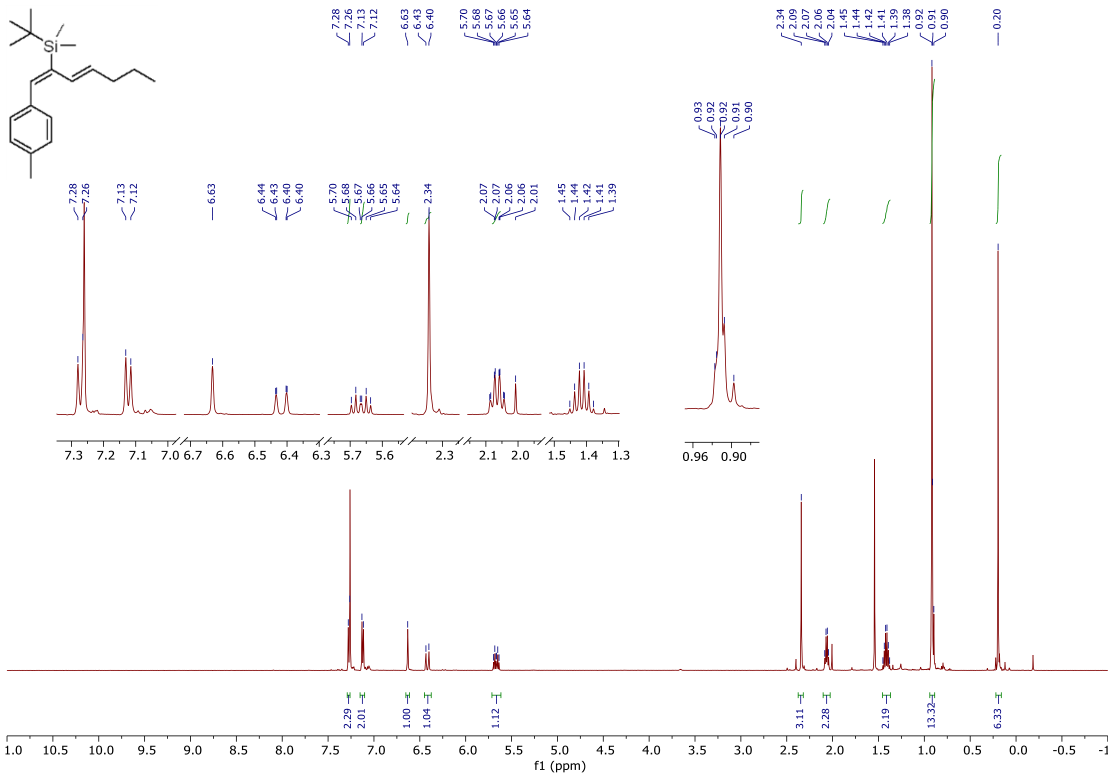

^{13}C {^{1}H} NMR (126 MHz, $CDCl_3$):

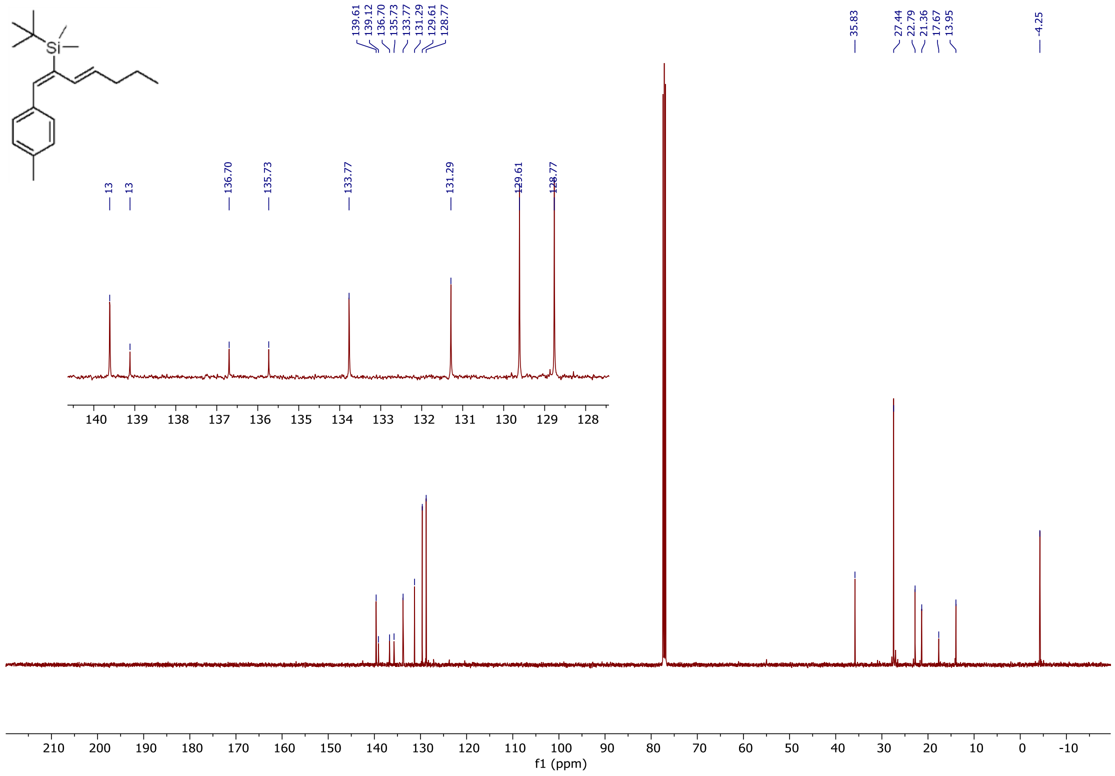

^{1}H-^{1}H-NOESY NMR ($CDCl_3$):

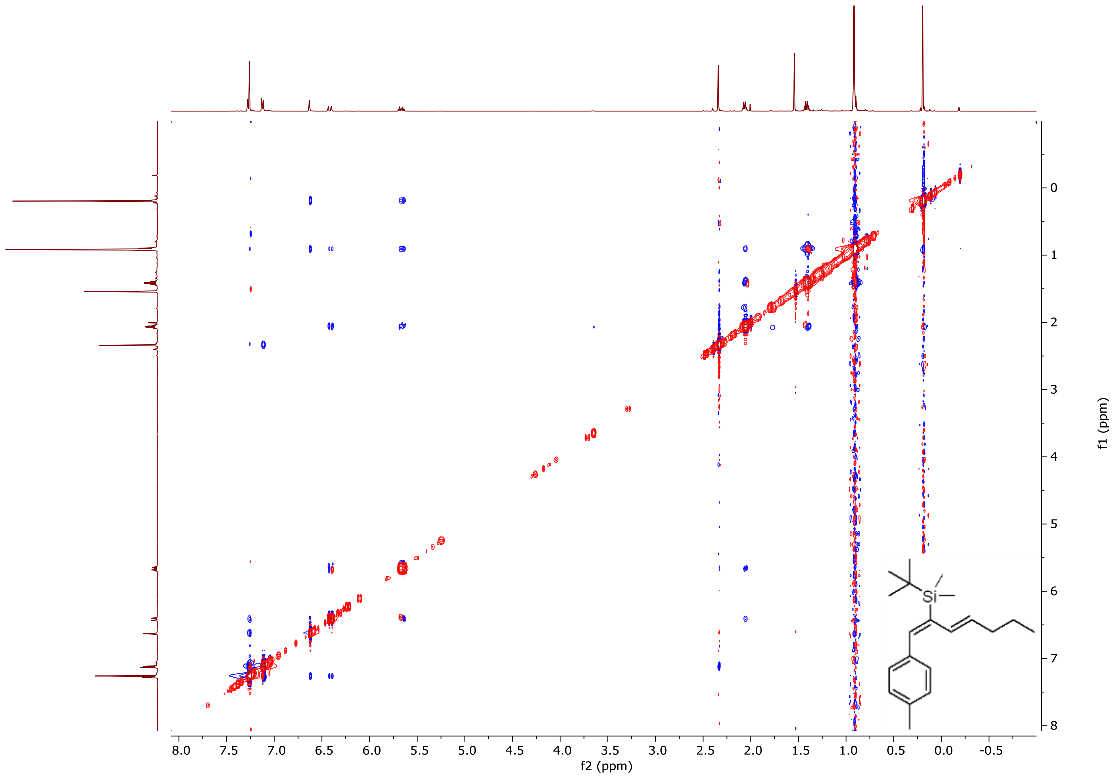

tert-Butyl(1-butyl-6-methyl-1*H*-inden-2-yl)dimethylsilane (**11c**)

^{1}H NMR (500 MHz, $CDCl_3$):

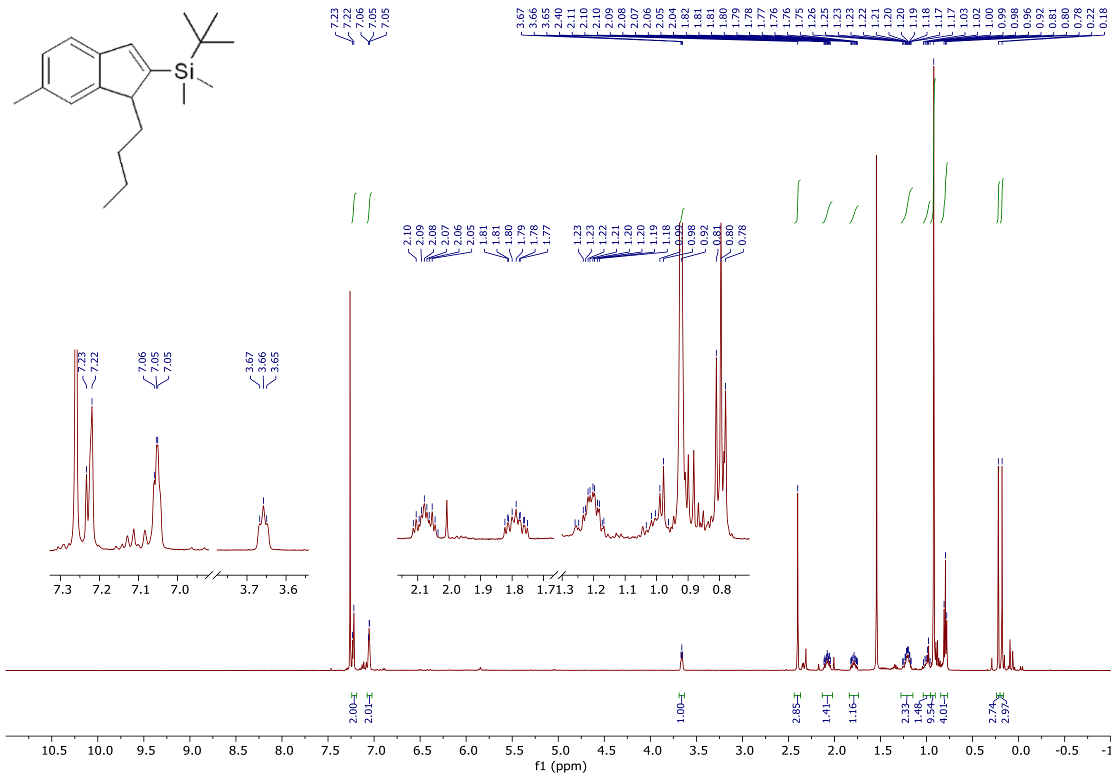

^{13}C {^{1}H} NMR (126 MHz, $CDCl_3$):

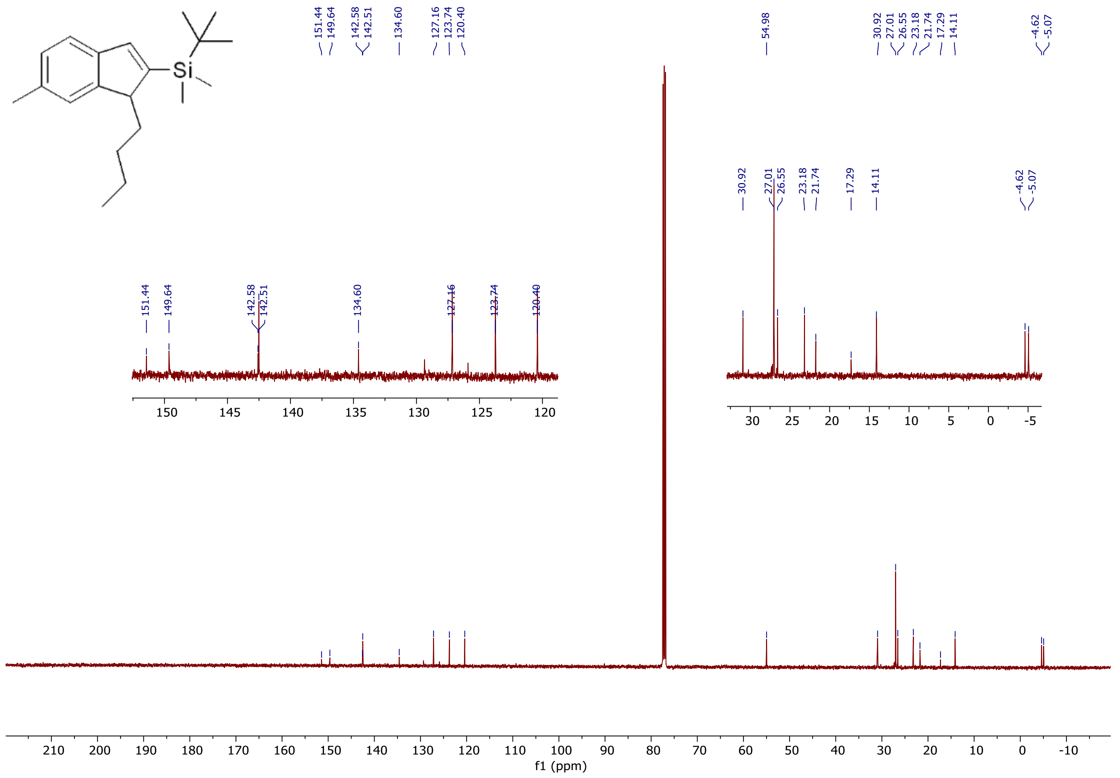

((1*E*,3*E*)-1-(4-Bromophenyl)hepta-1,3-dien-2-yl)(*tert*-butyl)dimethylsilane (**10d**)

^{1}H NMR (500 MHz, $CDCl_3$):

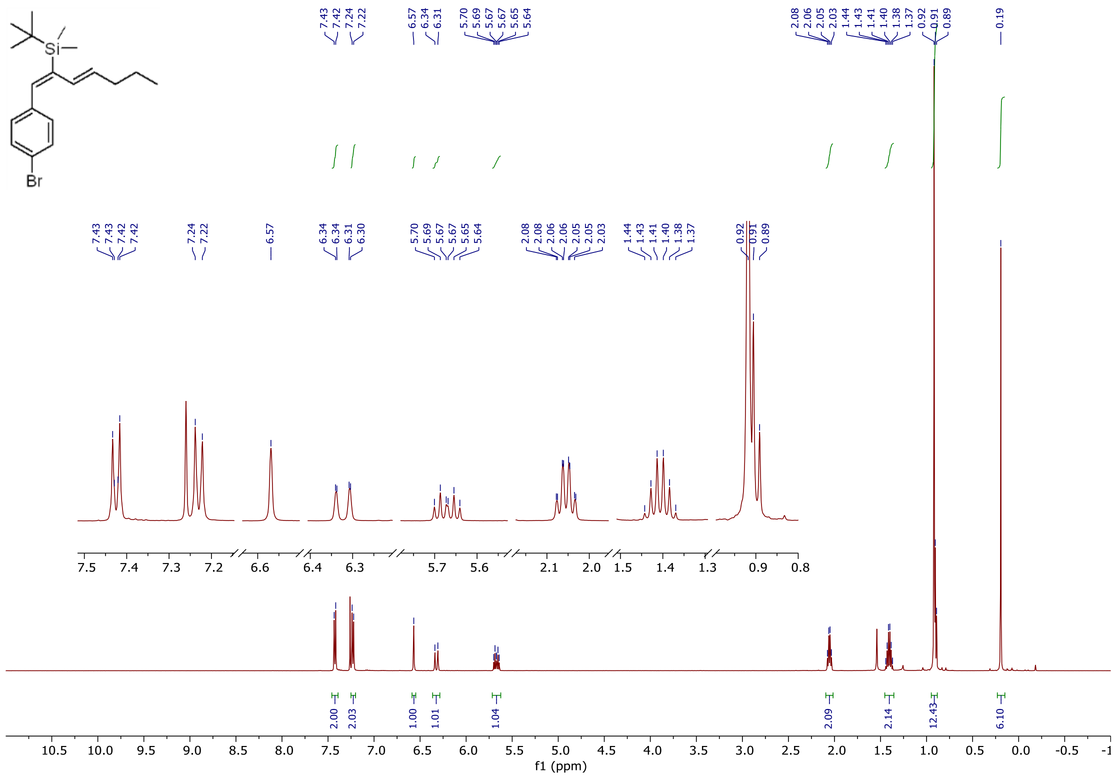

^{13}C {^{1}H} NMR (126 MHz, $CDCl_3$):

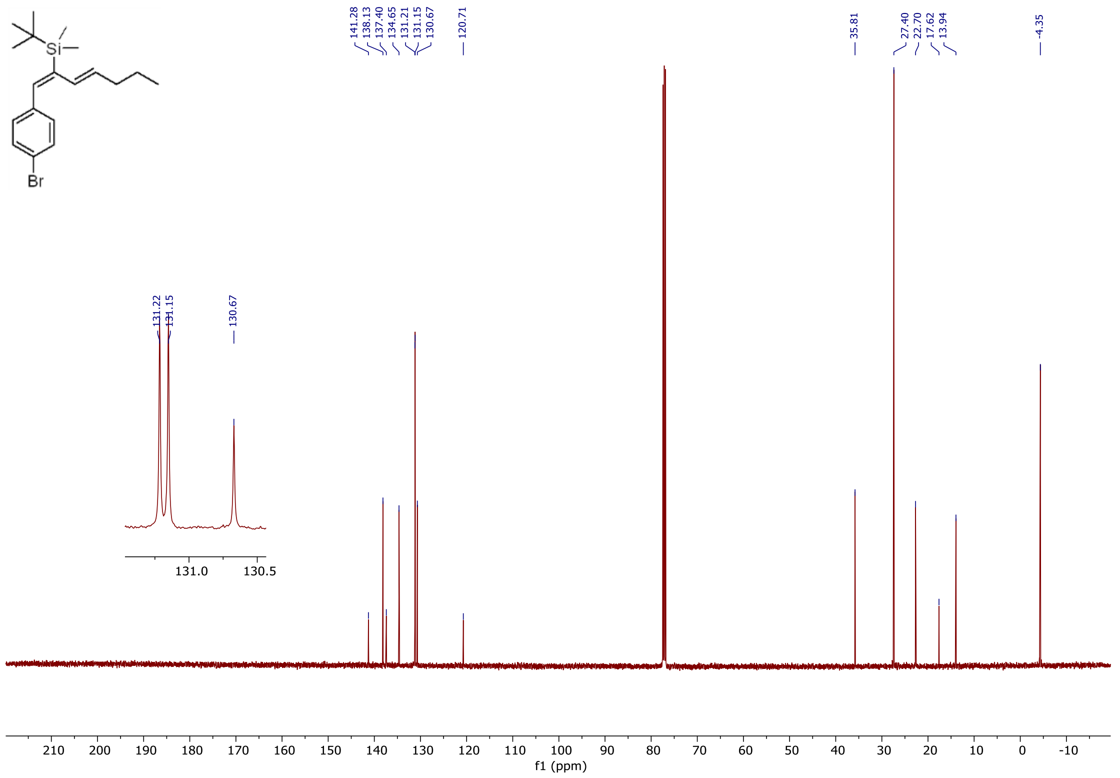

^1H-^1H-NOESY NMR ($CDCl_3$):

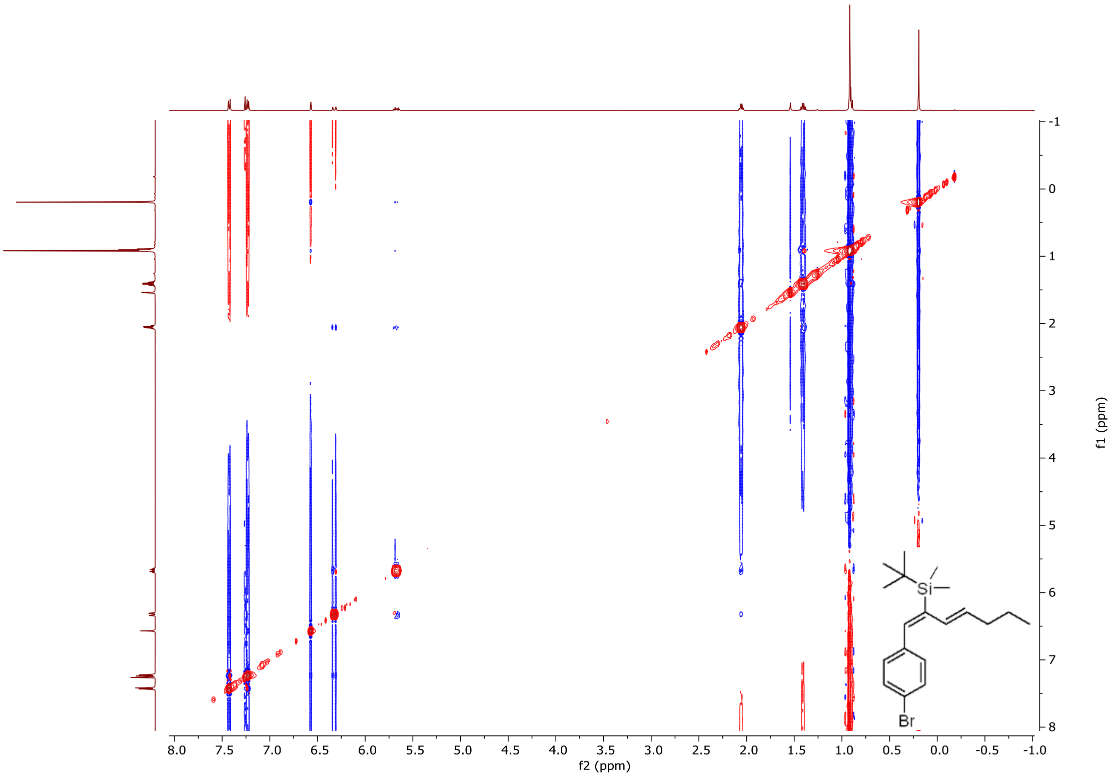

(*E*)-*tert*-Butyldimethyl(1-(tetrahydrofuran-2-yl)-2-(*p*-tolyl)vinyl)silane (**8b**)

^{1}H NMR (500 MHz, $CDCl_3$):

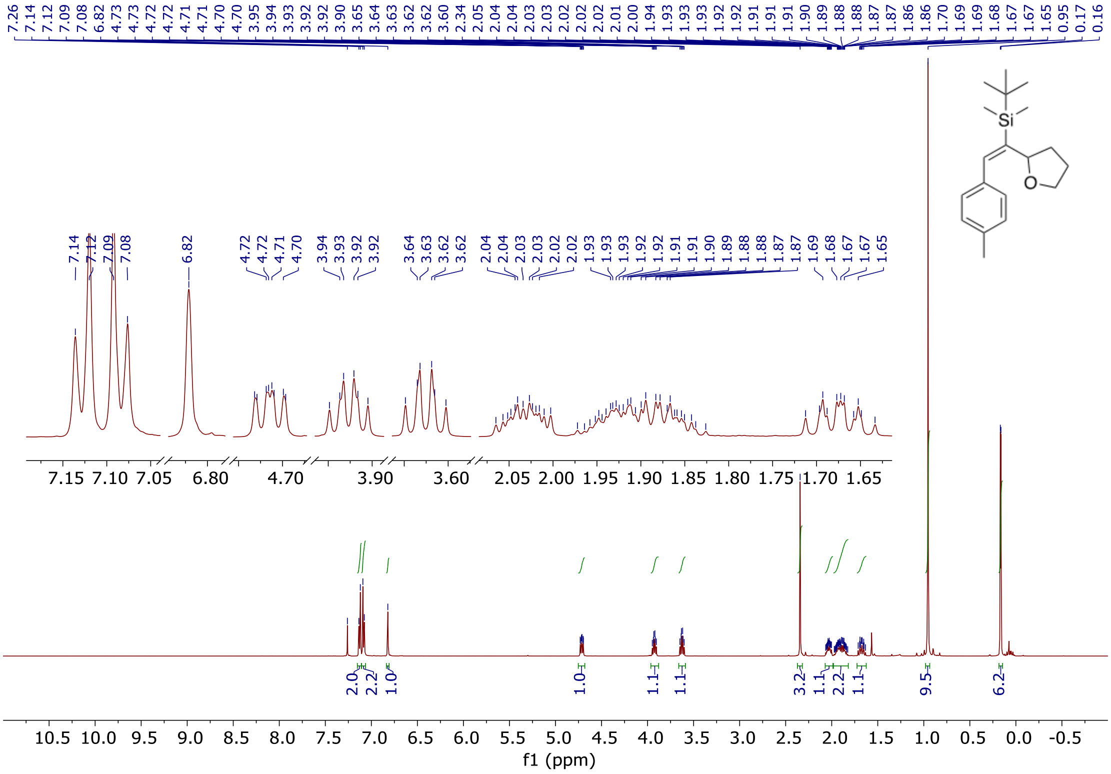

^{13}C{^{1}H} NMR (126 MHz, $CDCl_3$)

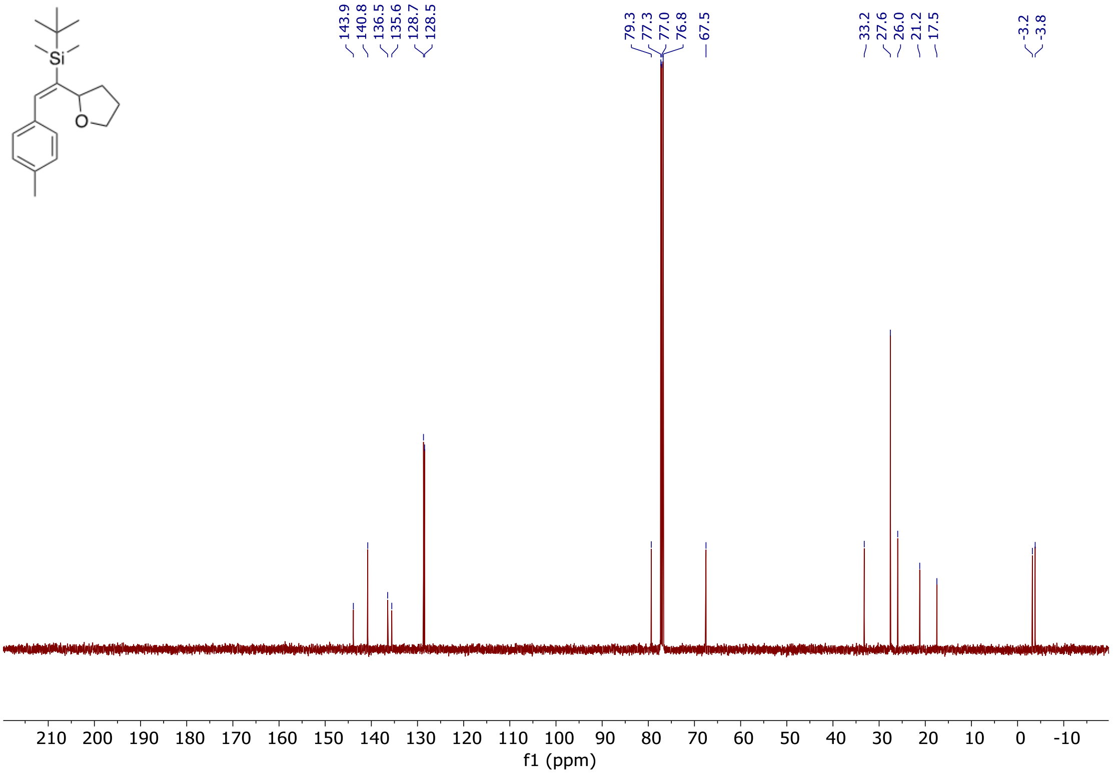

^{1}H-^{1}H-NOESY NMR ($CDCl_3$):

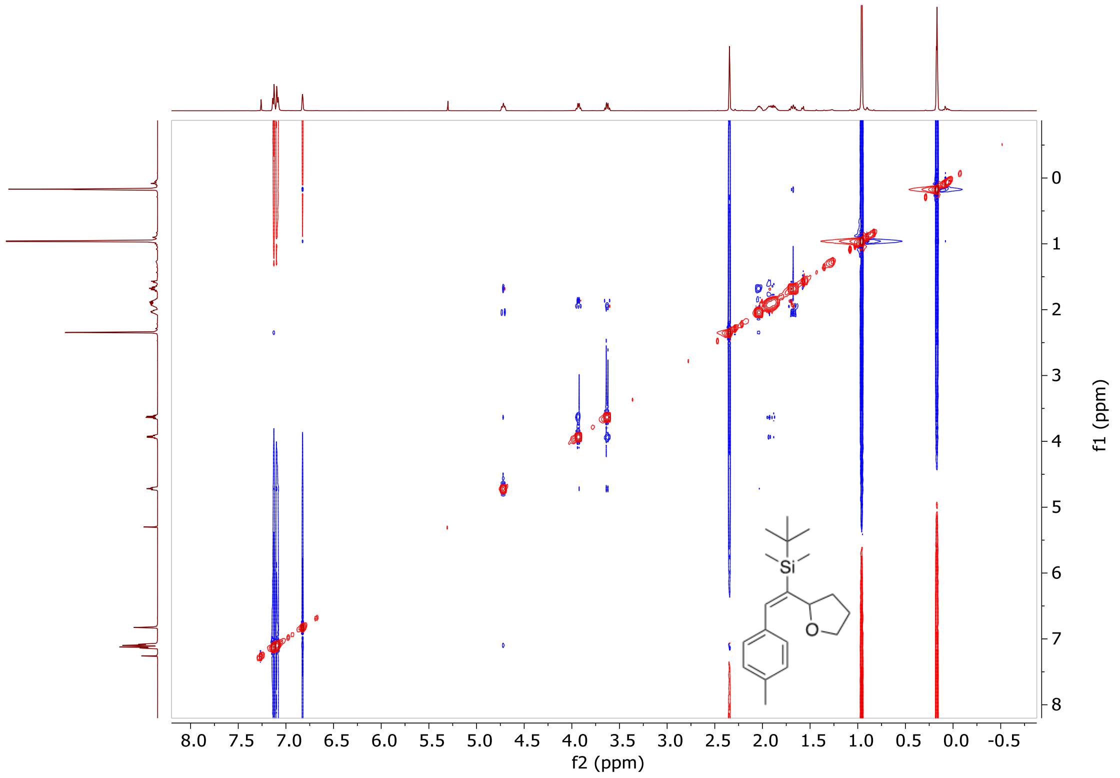

(*E*)-*tert*-Butyl(2-(3-methoxyphenyl)-1-(tetrahydrofuran-2-yl)vinyl)dimethylsilane (**8d**)

^{1}H NMR (500 MHz, $CDCl_3$):

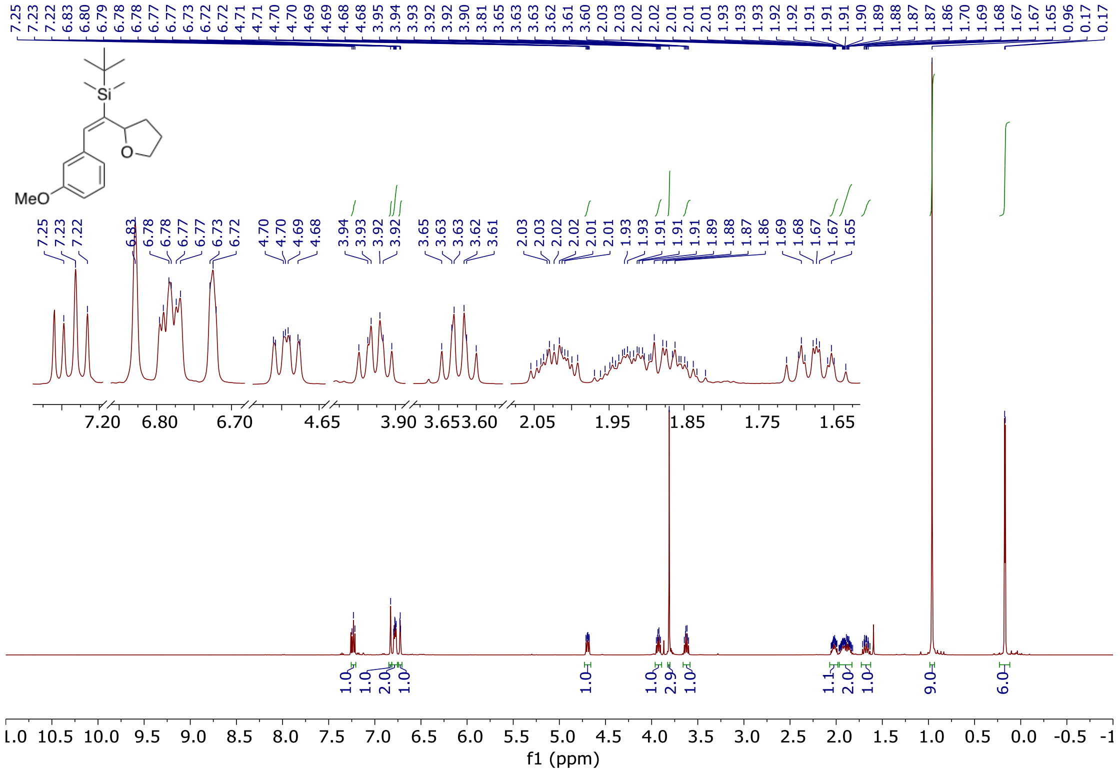

^{13}C{^{1}H} NMR (126 MHz, $CDCl_3$):

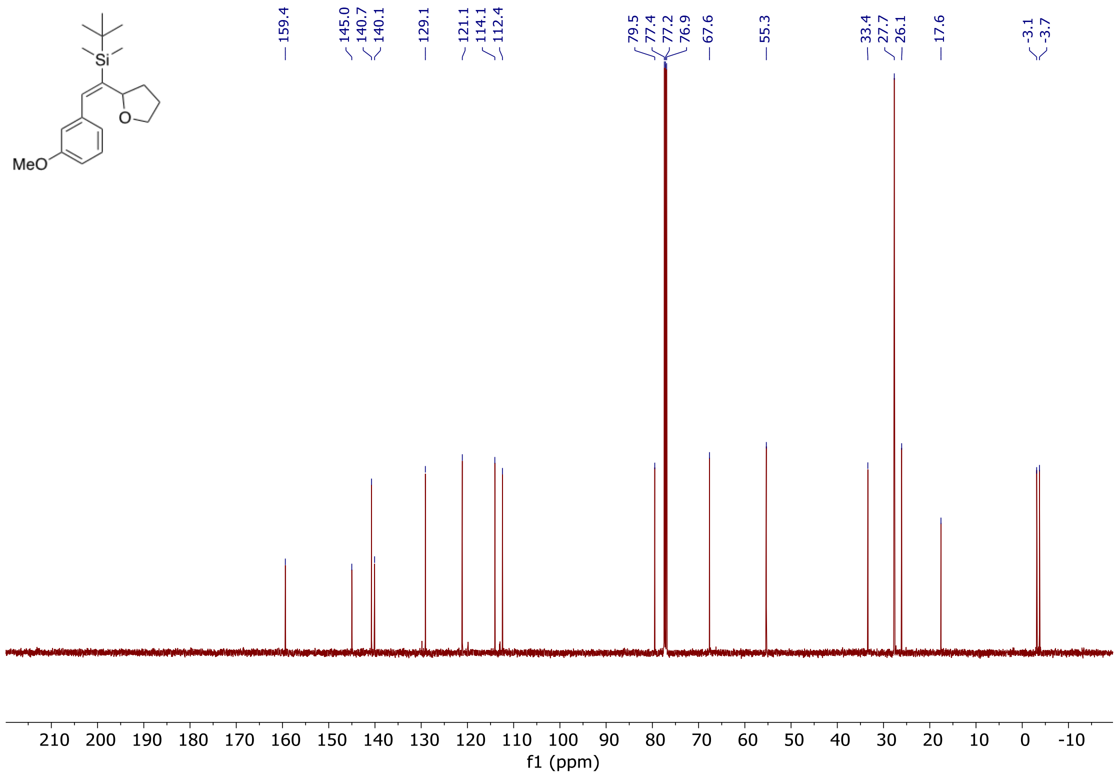

(*E*)-(2-(4-Bromophenyl)-1-(tetrahydrofuran-2-yl)vinyl)(*tert*-butyl)dimethylsilane (**8f**):

^{1}H NMR (500 MHz, $CDCl_3$):

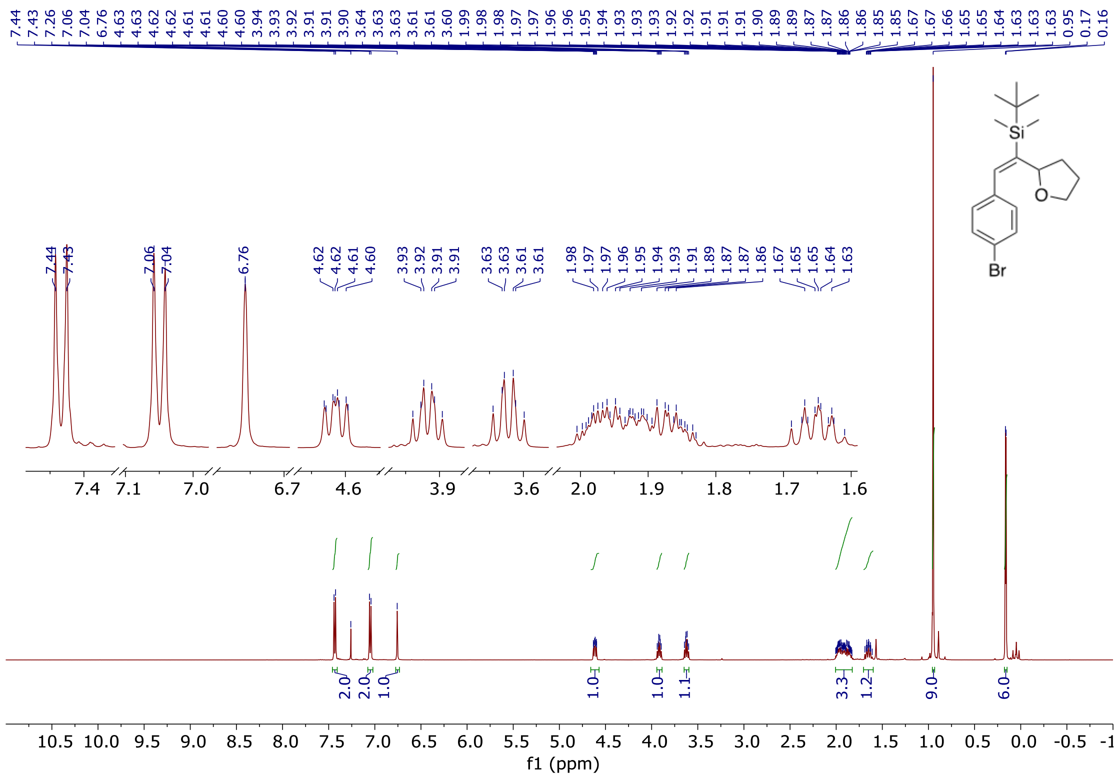

$^{13}C\{^1H\}$ NMR (126 MHz, $CDCl_3$)

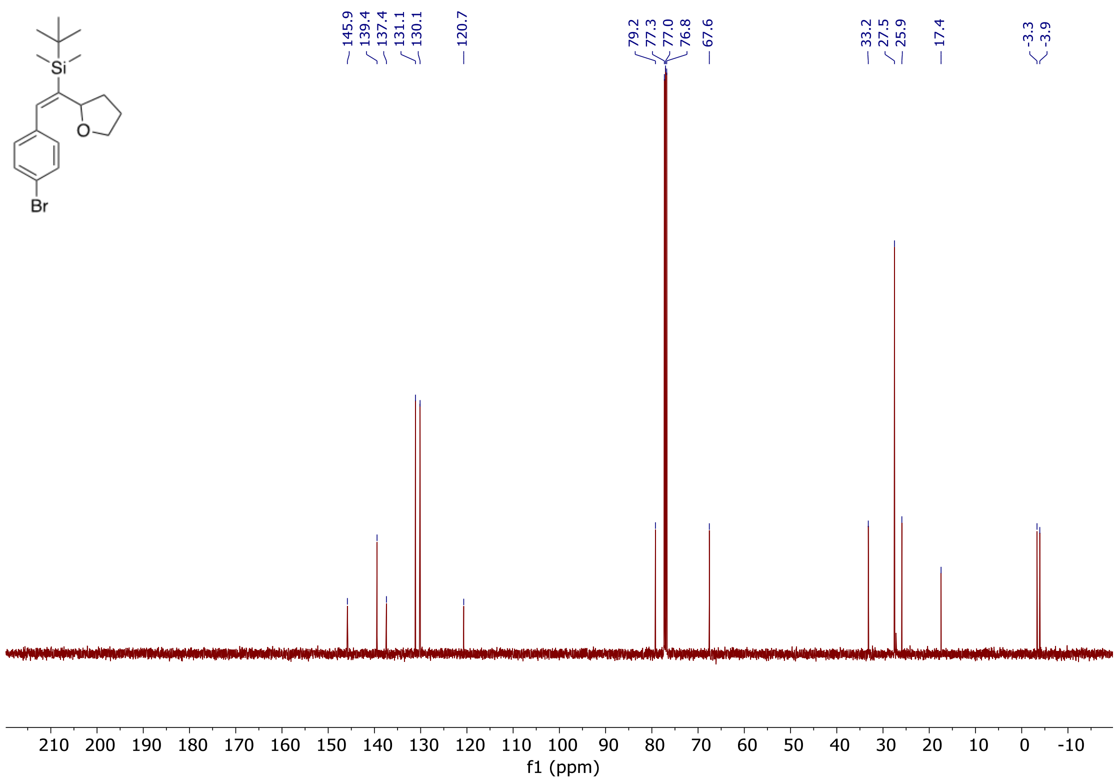

(*E*)-(2-(4-Fluorophenyl)-1-(tetrahydrofuran-2-yl)vinyl)(*tert*-butyl)dimethylsilane (**8h**)

^{1}H NMR (500 MHz, $CDCl_3$):

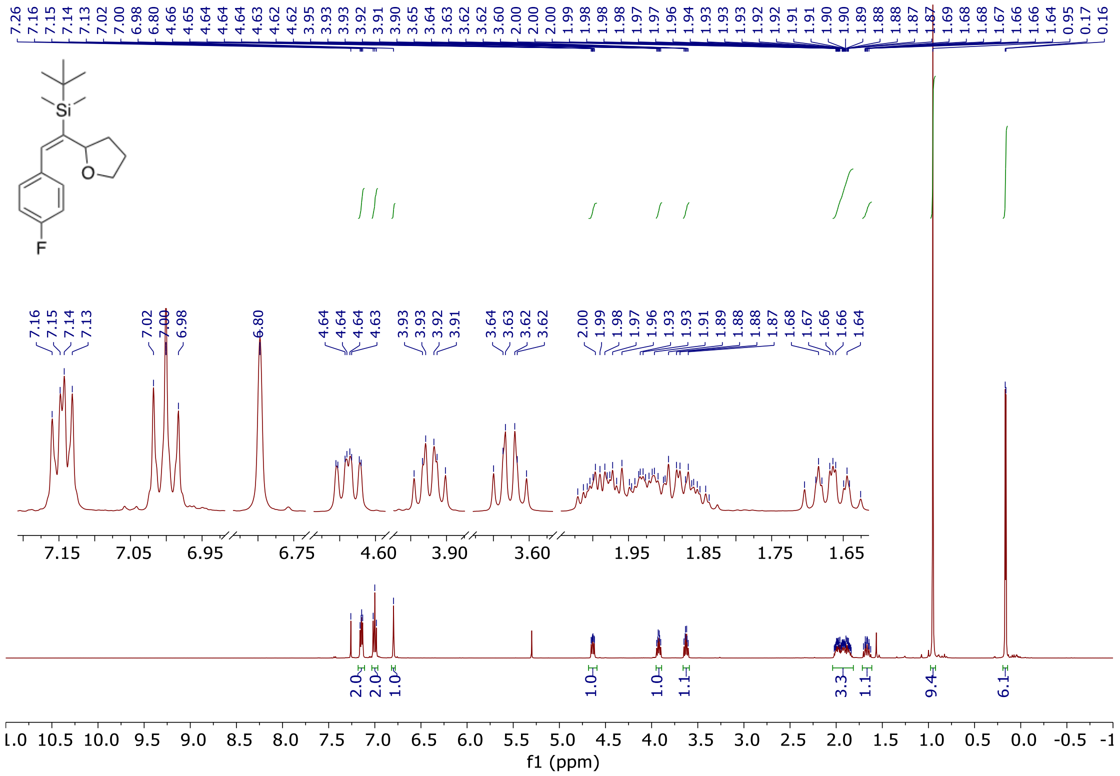

^{13}C{^{1}H} NMR (126 MHz, $CDCl_3$)

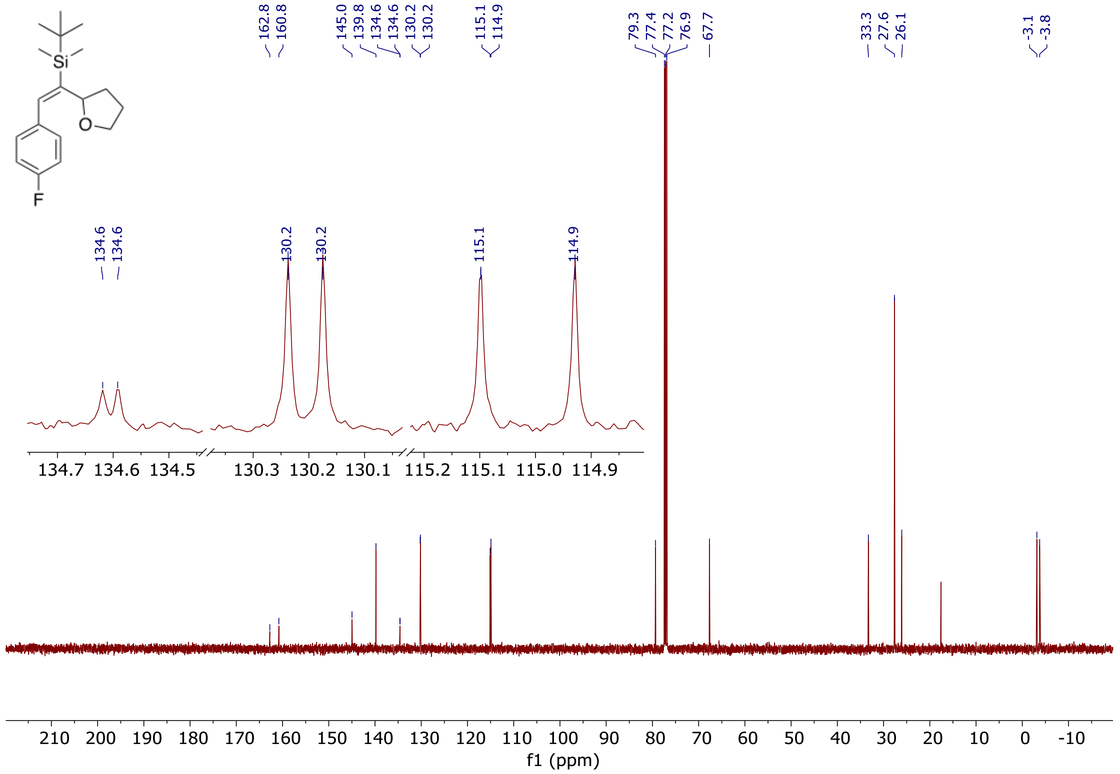

(*E*)-4-(2-(*tert*-Butyldimethylsilyl)-2-(tetrahydrofuran-2-yl)vinyl)benzonitrile (**8j**)

^{1}H NMR (500 MHz, $CDCl_3$):

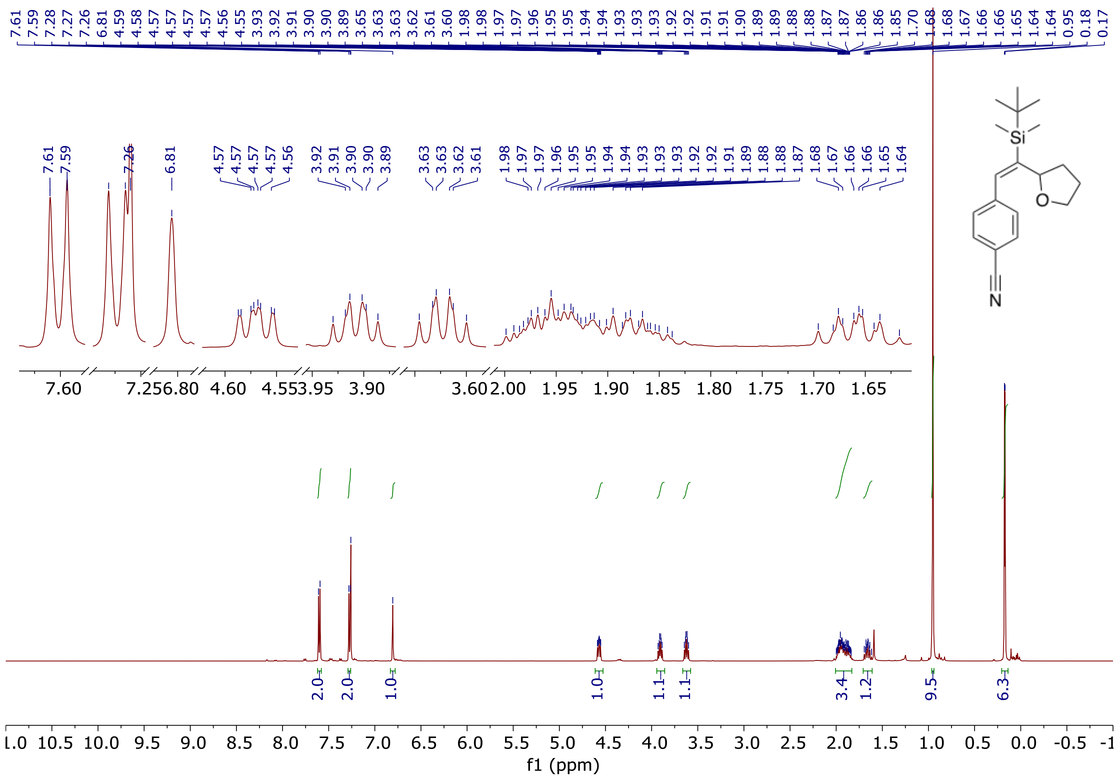

$^{13}C\{^{1}H\}$ NMR (126 MHz, $CDCl_3$):

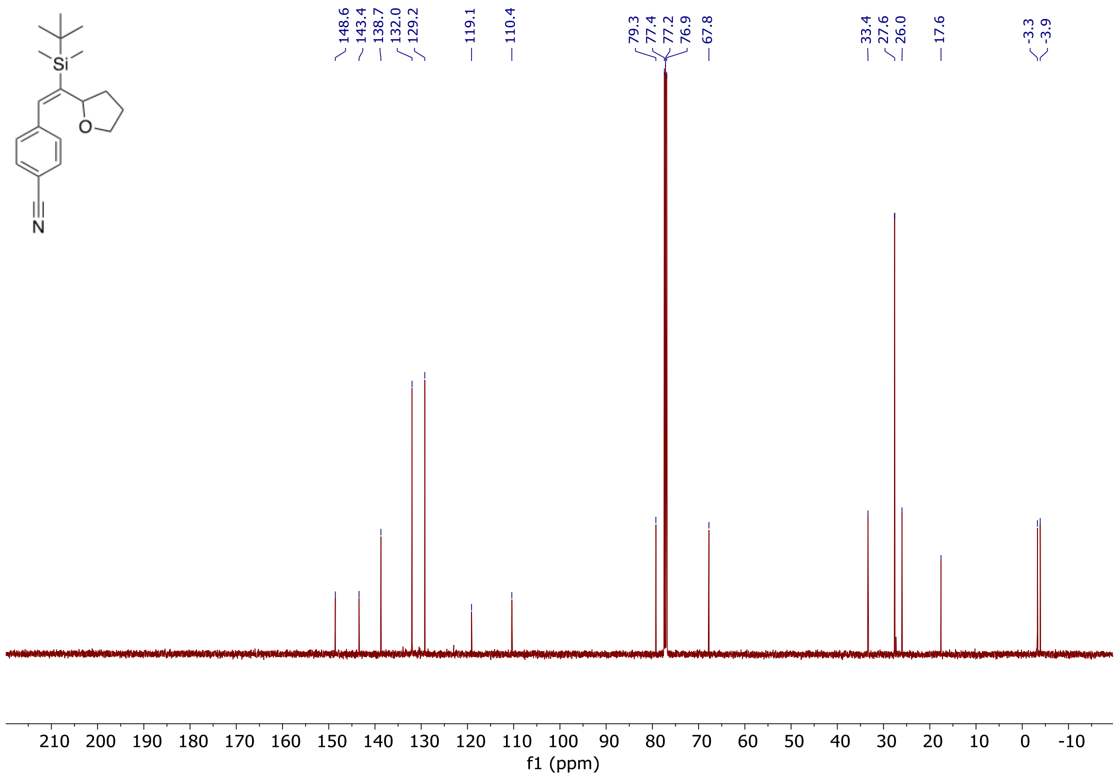

(*E*)-1-(4-(2-(*tert*-Butyldimethylsilyl)-2-(tetrahydrofuran-2-yl)vinyl)phenyl)ethan-1-one (**8k**)

^{1}H NMR (500 MHz, $CDCl_3$):

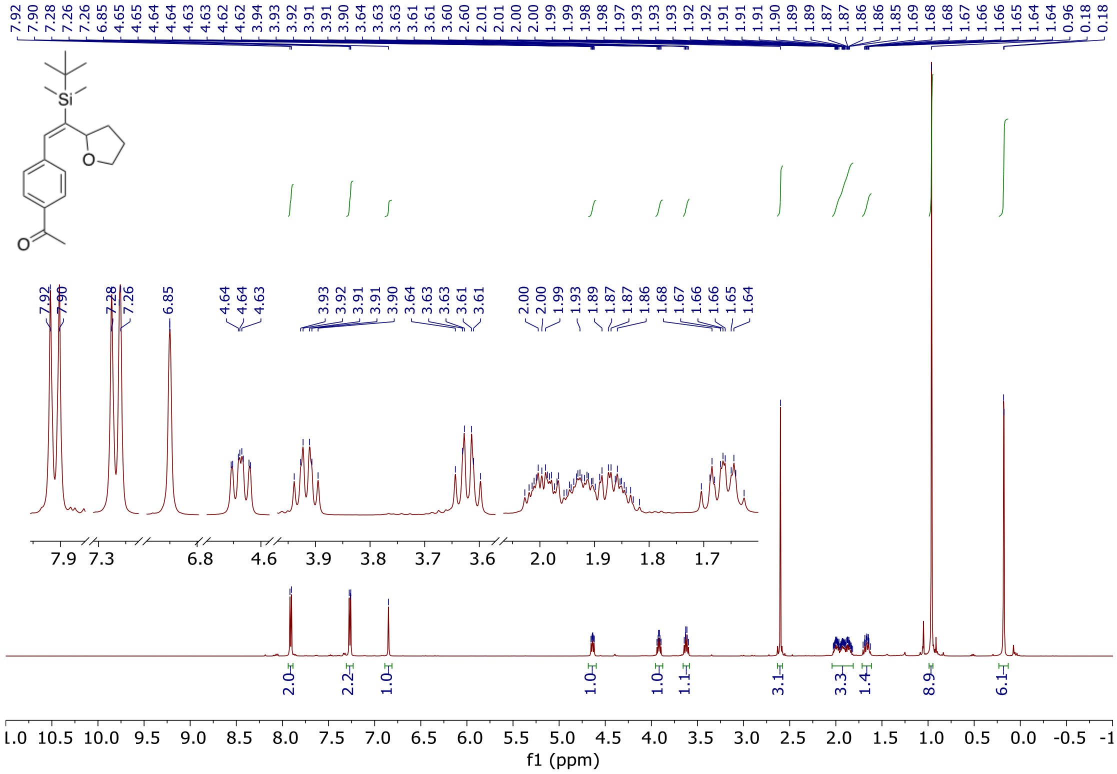

^{13}C{^{1}H} NMR (126 MHz, $CDCl_3$):

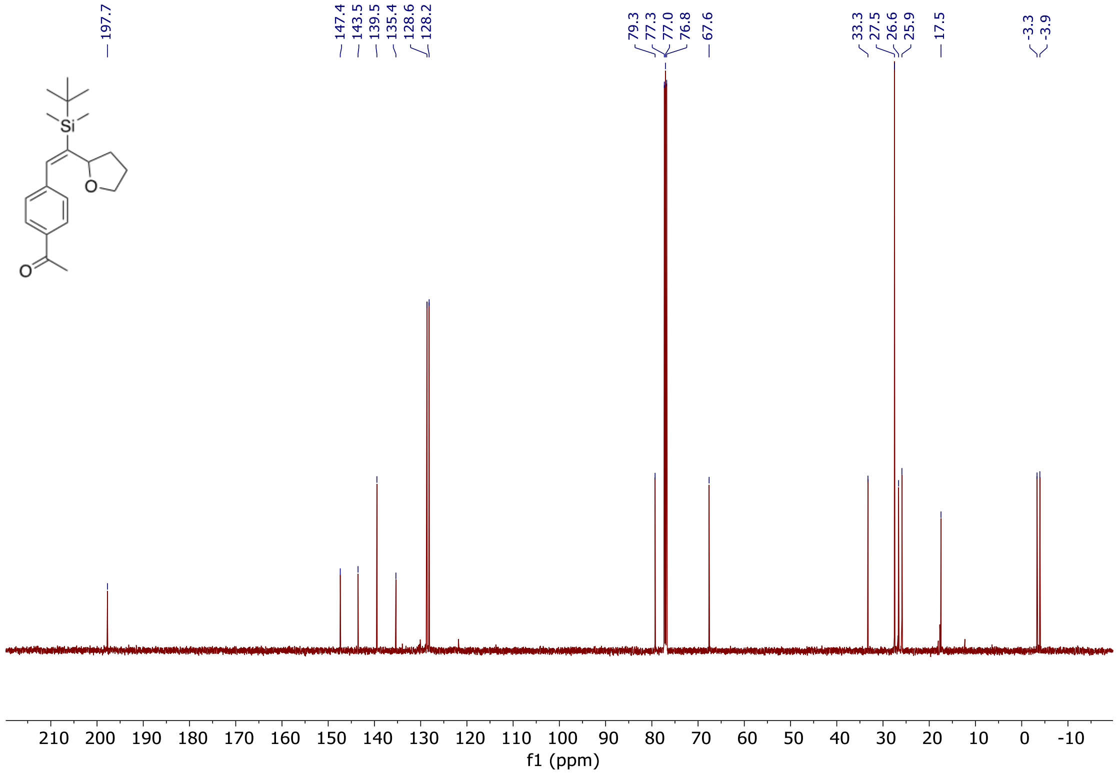

Methyl (*E*)-4-(2-(*tert*-butyldimethylsilyl)-2-(tetrahydrofuran-2-yl)vinyl)benzoate (**8l**)

^{1}H NMR (500 MHz, $CDCl_3$):

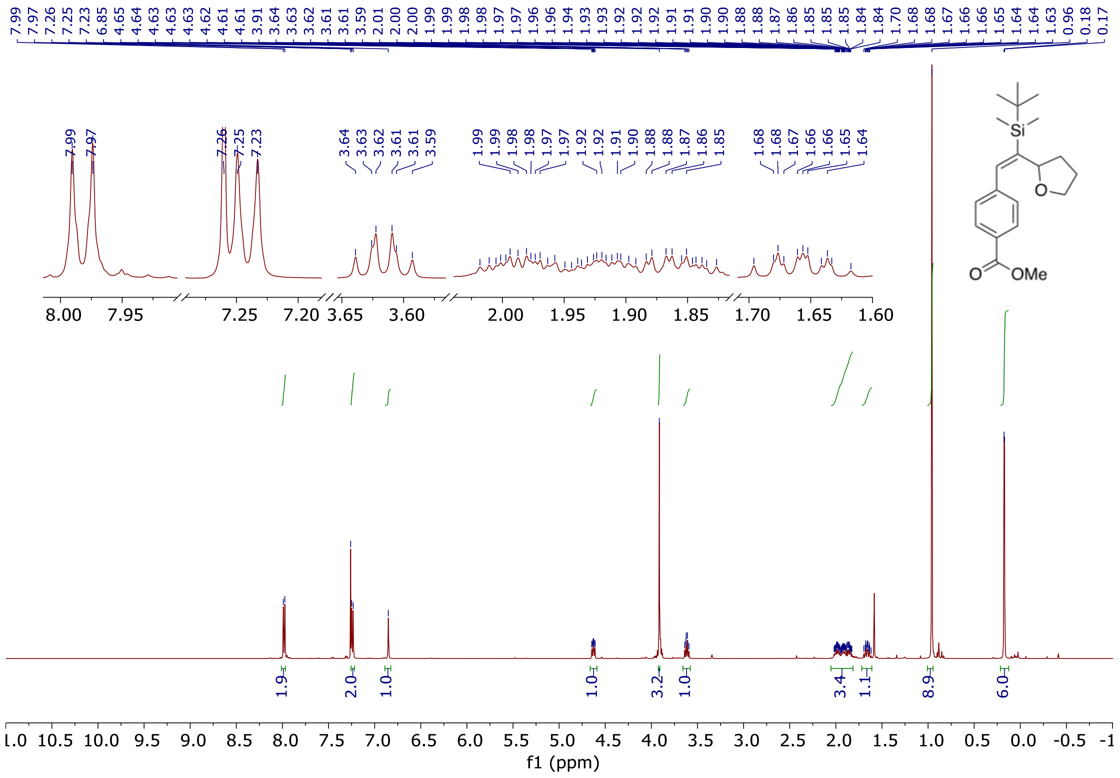

$^{13}C\{^1H\}$ NMR (126 MHz, $CDCl_3$):

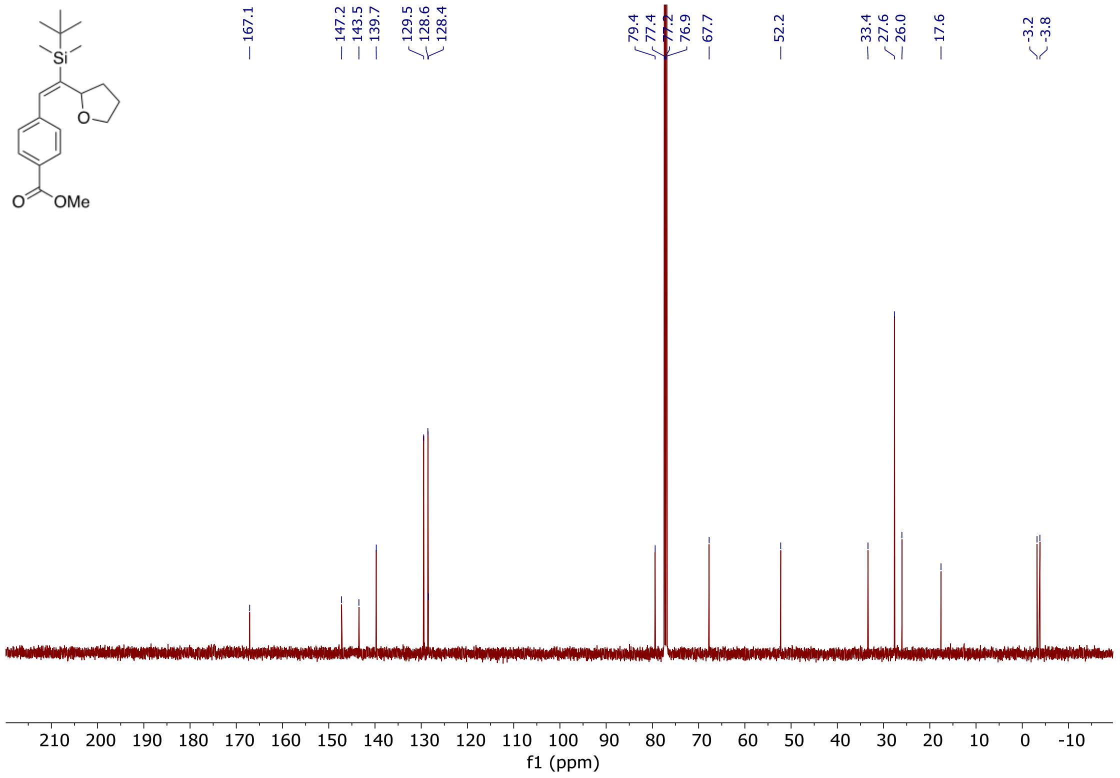

(*E*)-*tert*-Butyldimethyl(1-(tetrahydrofuran-2-yl)-2-(4-(trifluoromethyl)phenyl)vinyl)silane (**8m**)

^{1}H NMR (500 MHz, $CDCl_3$):

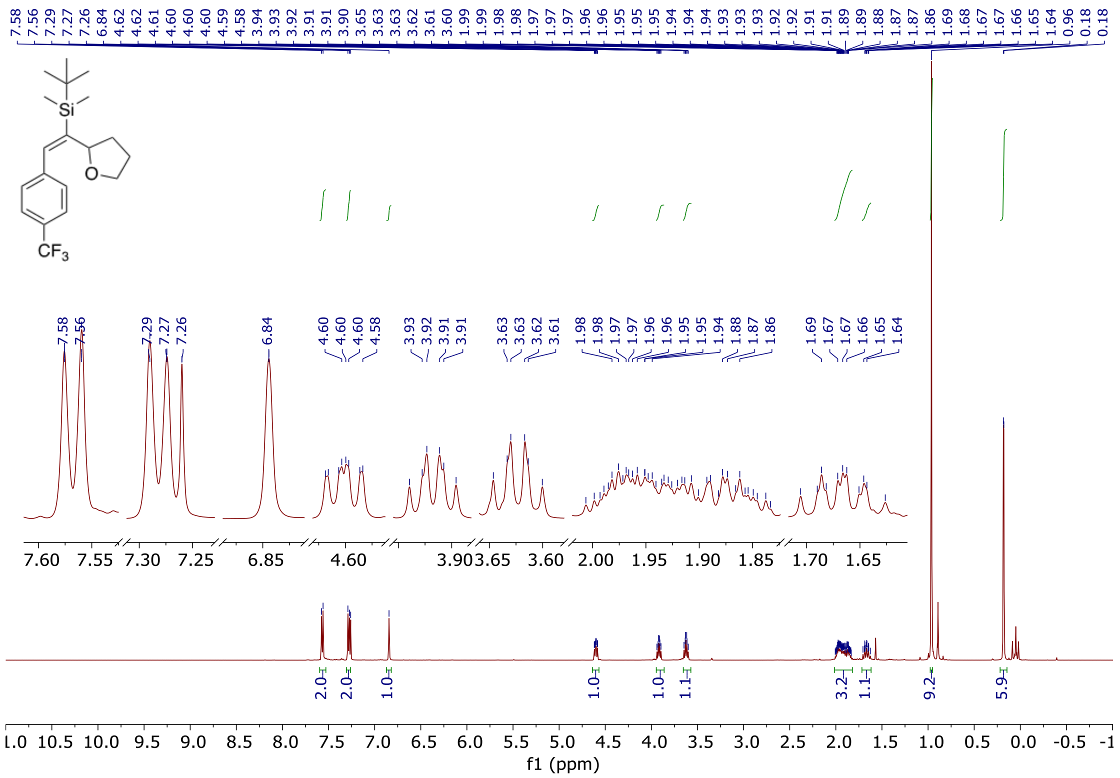

^{13}C{^{1}H} NMR (126 MHz, $CDCl_3$)

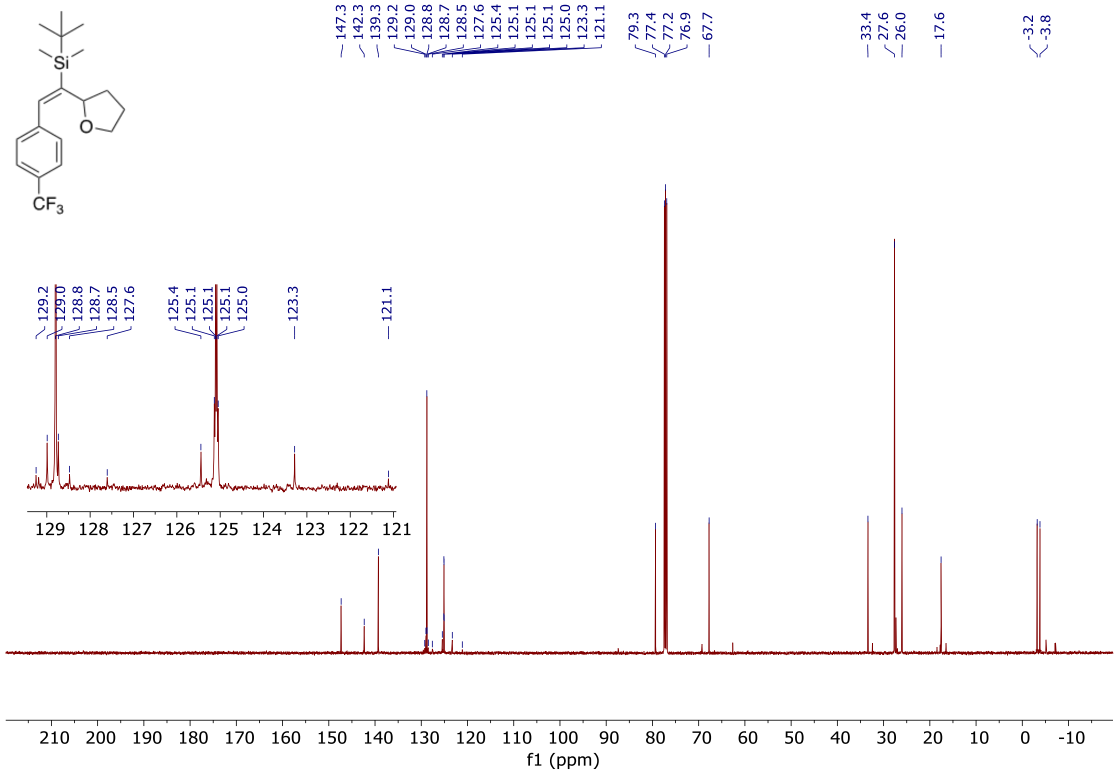

(*E*)-*tert*-Butyldimethyl(1-(tetrahydrofuran-2-yl)-2-(thiophen-2-yl)vinyl)silane (**8o**)

^{1}H NMR (500 MHz, $CDCl_3$):

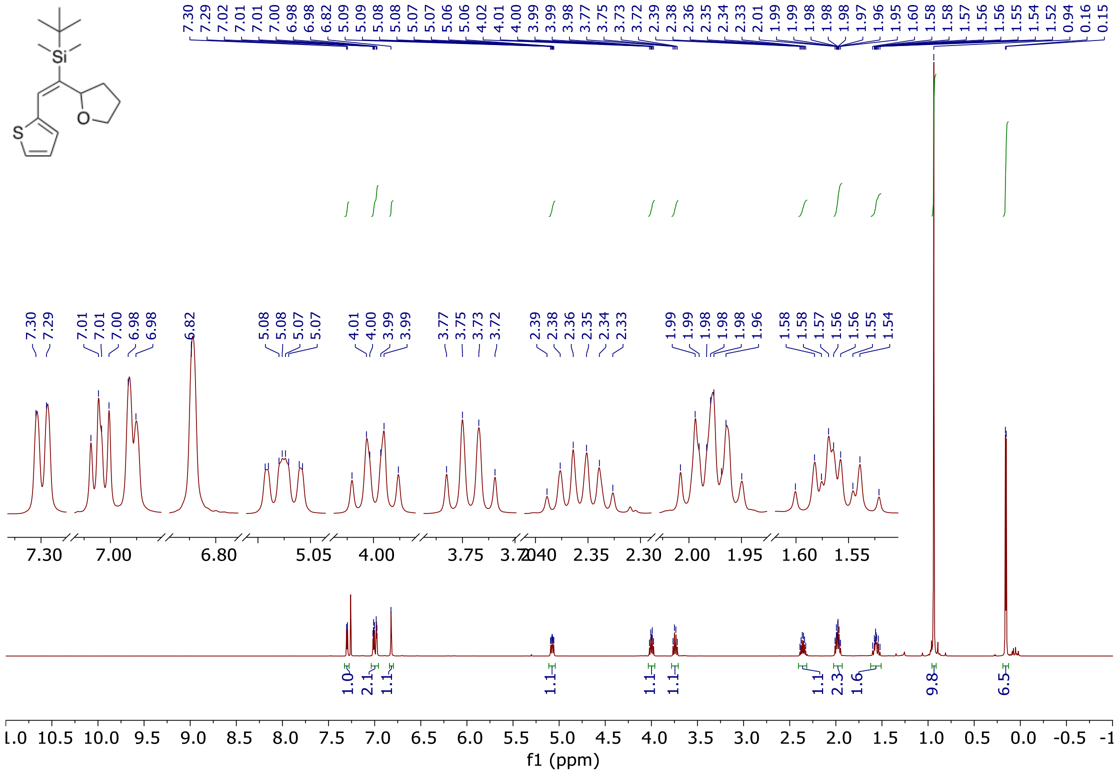

$^{13}C\{^{1}H\}$ NMR (126 MHz, $CDCl_3$)

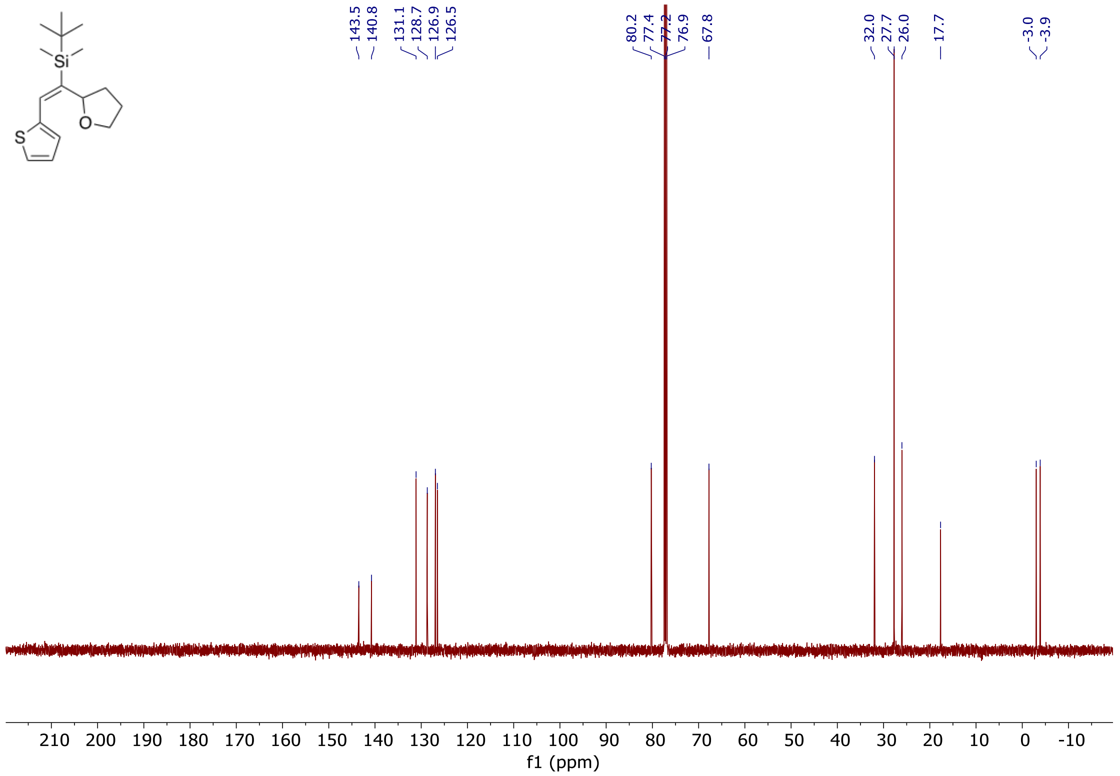

(*E*)-5-(1-(*tert*-Butyldimethylsilyl)-2-phenylvinyl)dihydrofuran-2(3*H*)-one (**8t**)

^{1}H NMR (500 MHz, DCM-d_2):

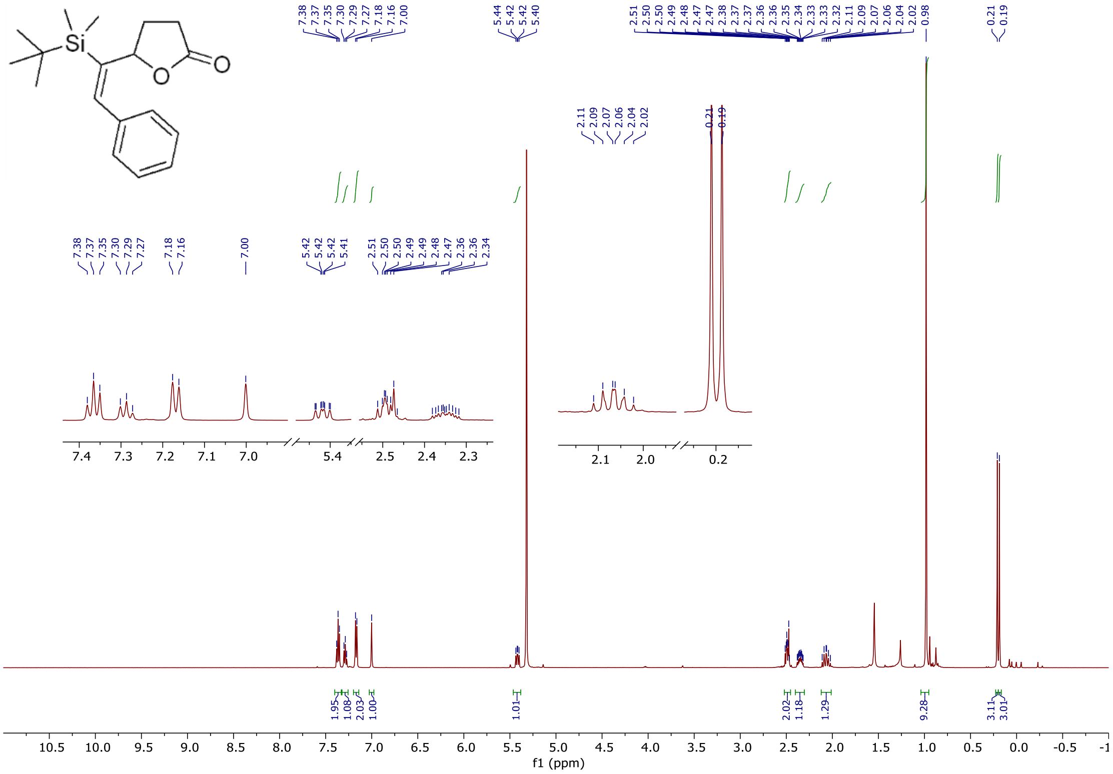

^{13}C {^{1}H} NMR (126 MHz, $CDCl_3$)

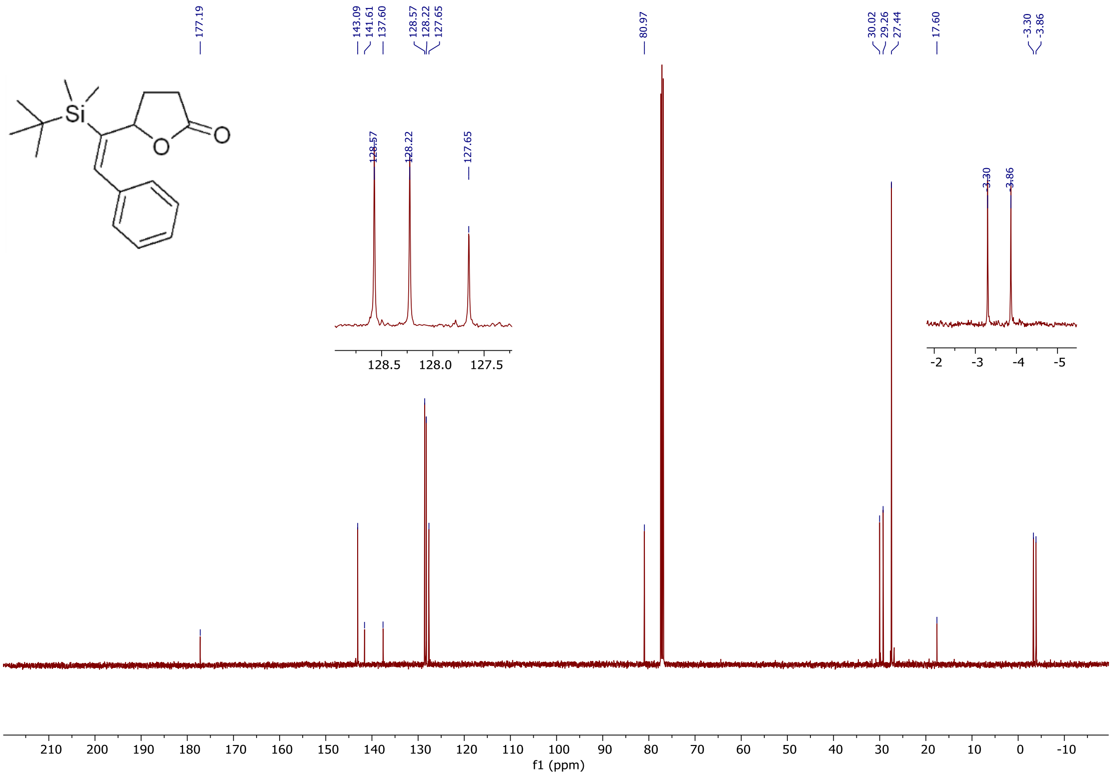

(*E*)-(2-(1-(*tert*-Butyldimethylsilyl)-2-phenylvinyl)pyrrolidin-1-yl)(phenyl)methanone (**8u**)

^{1}H NMR (500 MHz, DMSO-d_6, 80 °C):

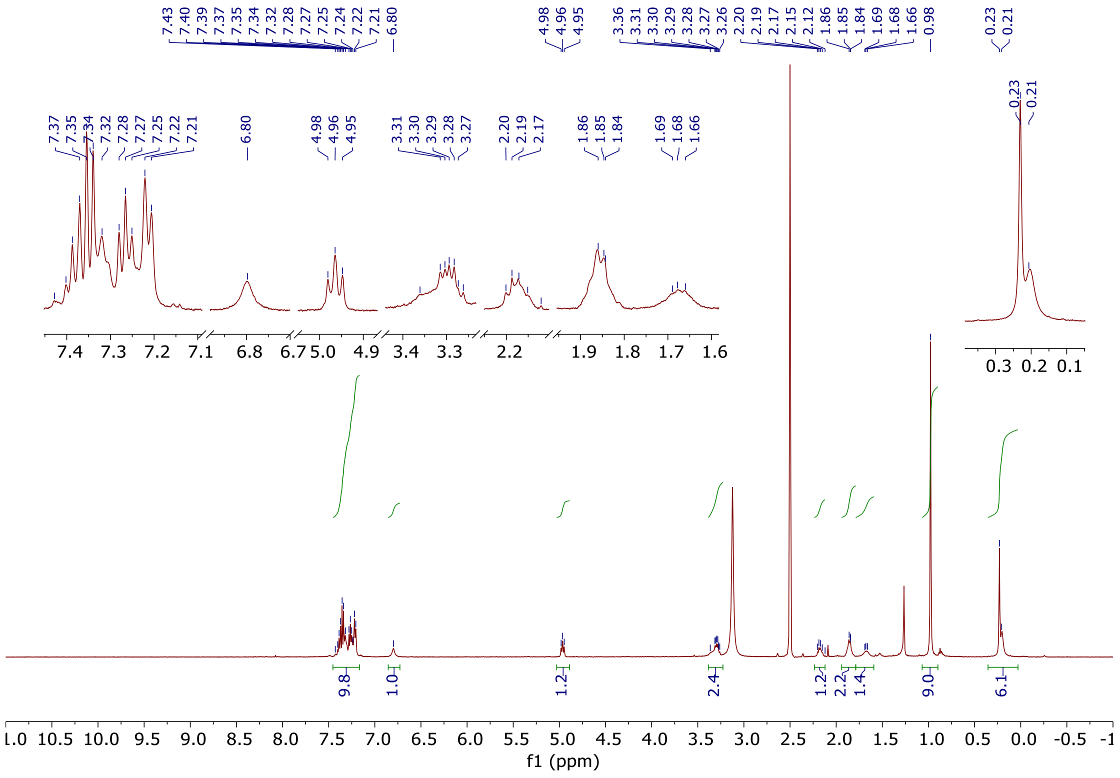

^{13}C {^{1}H} NMR (126 MHz, DMSO-d_6, 25 °C)

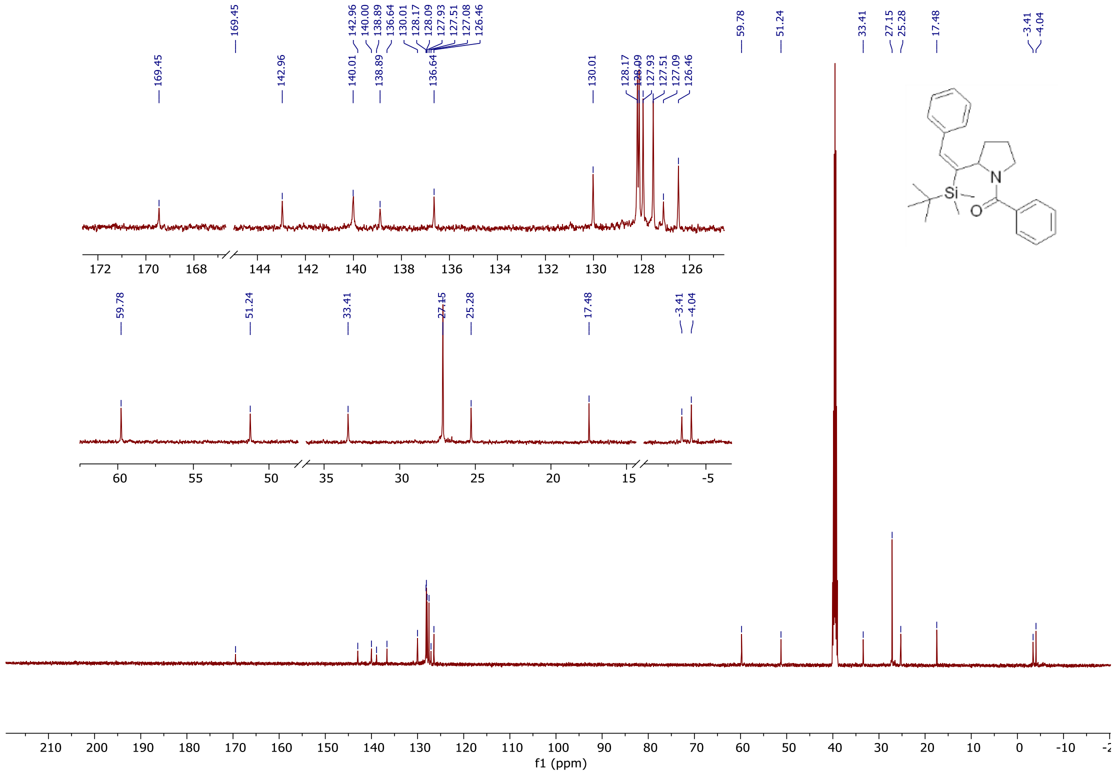

^1H-^1H-NOESY NMR (DMSO-d_6, 25 °C)

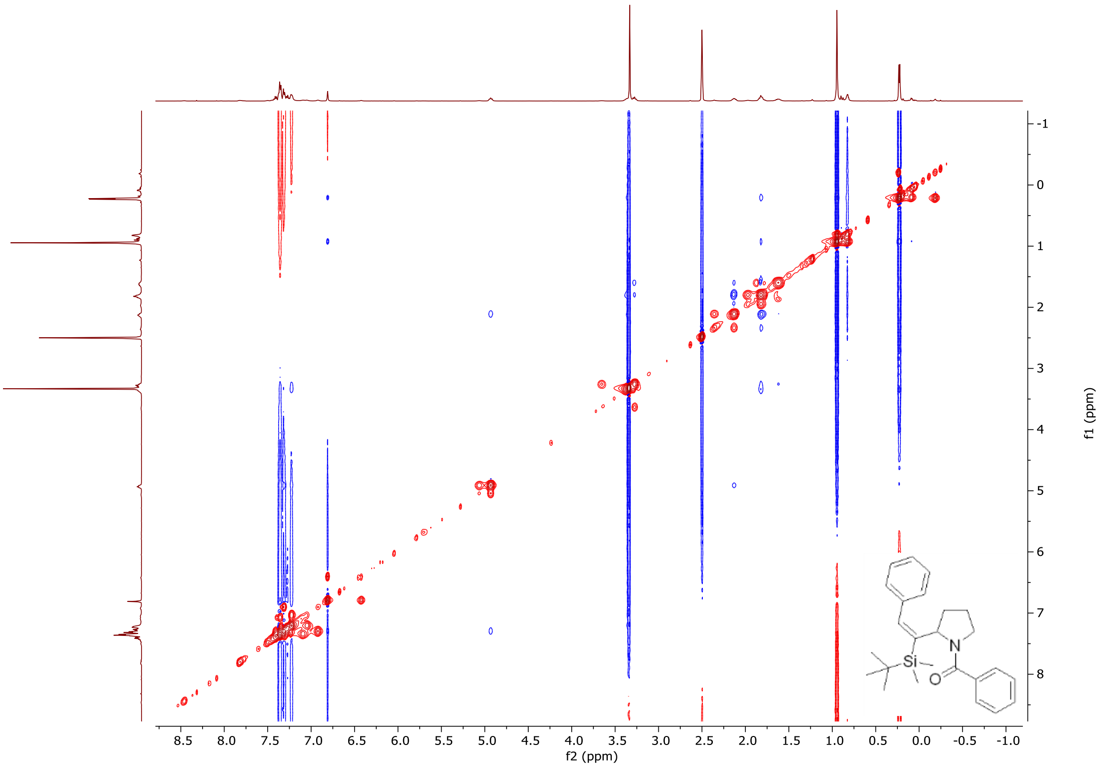

1-(5-(*tert*-Butyldimethylsilyl)-6-phenyl-3,6-dihydropyridin-1(2*H*)-yl)ethan-1-one (**9a**)

^{1}H NMR (500 MHz, $CDCl_3$):

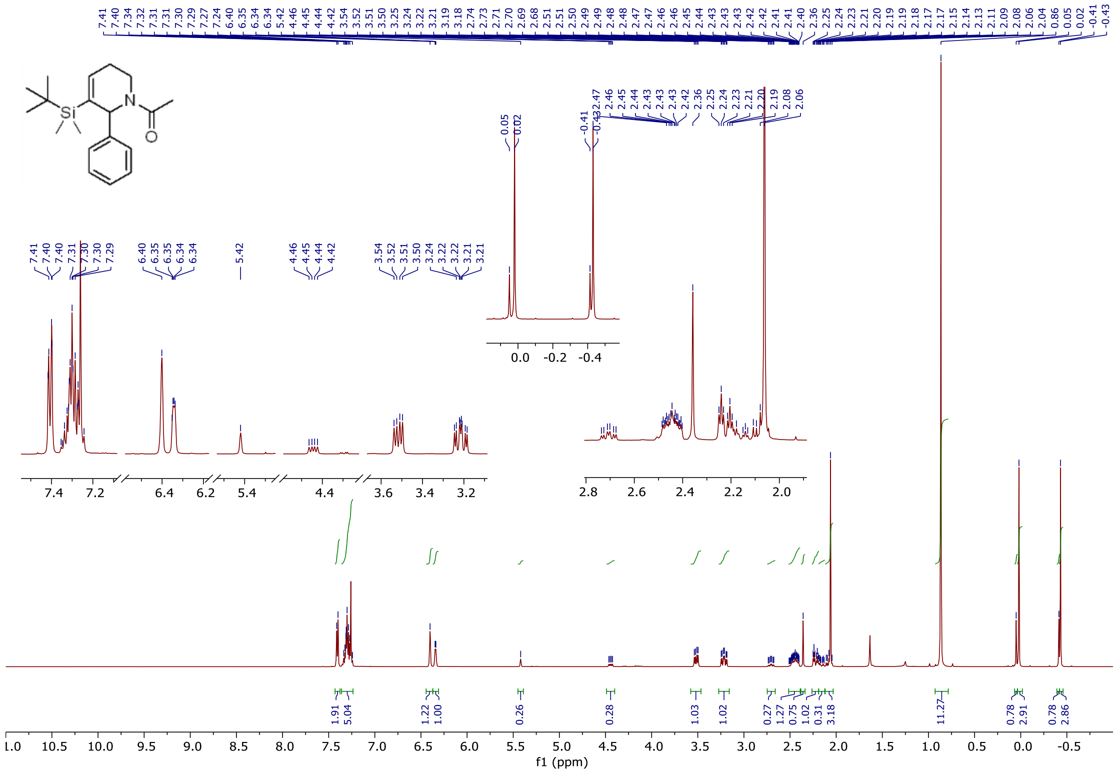

^{1}H NMR (500 MHz, DMSO-d_6, 80 °C)

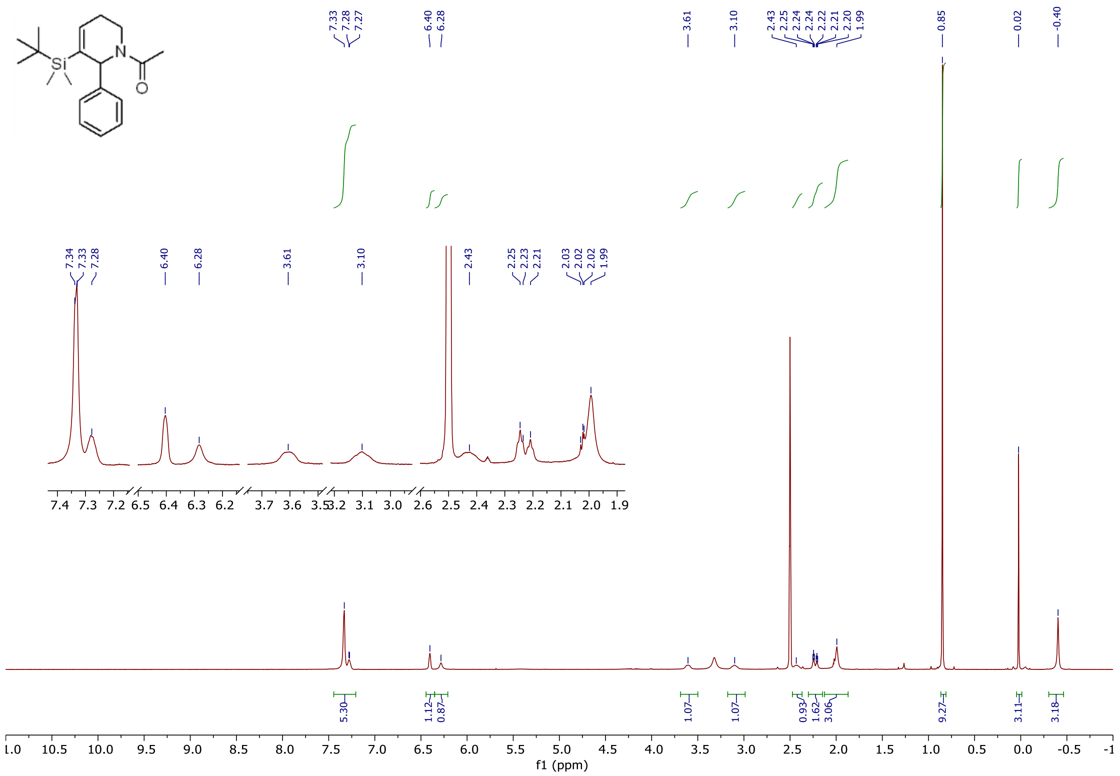

$^{13}C\ \{^{1}H\}$ NMR (126 MHz, $CDCl_3$)

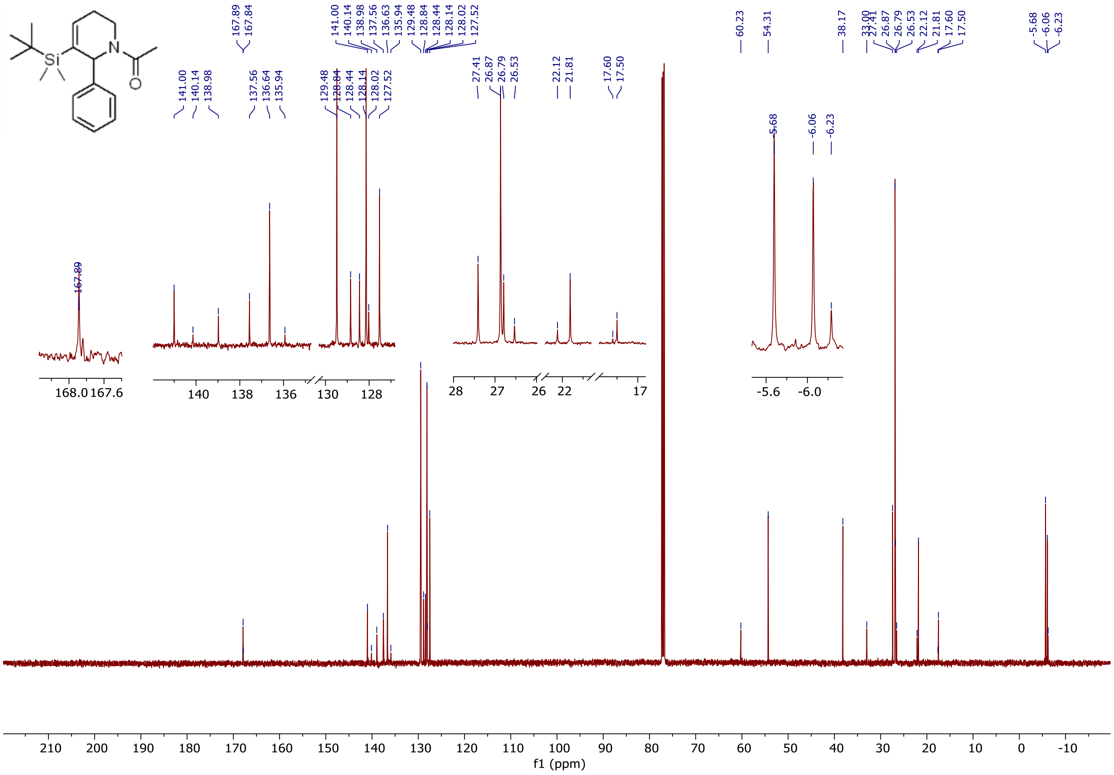

^{1}H-^{13}C-HSQC NMR ($CDCl_3$)

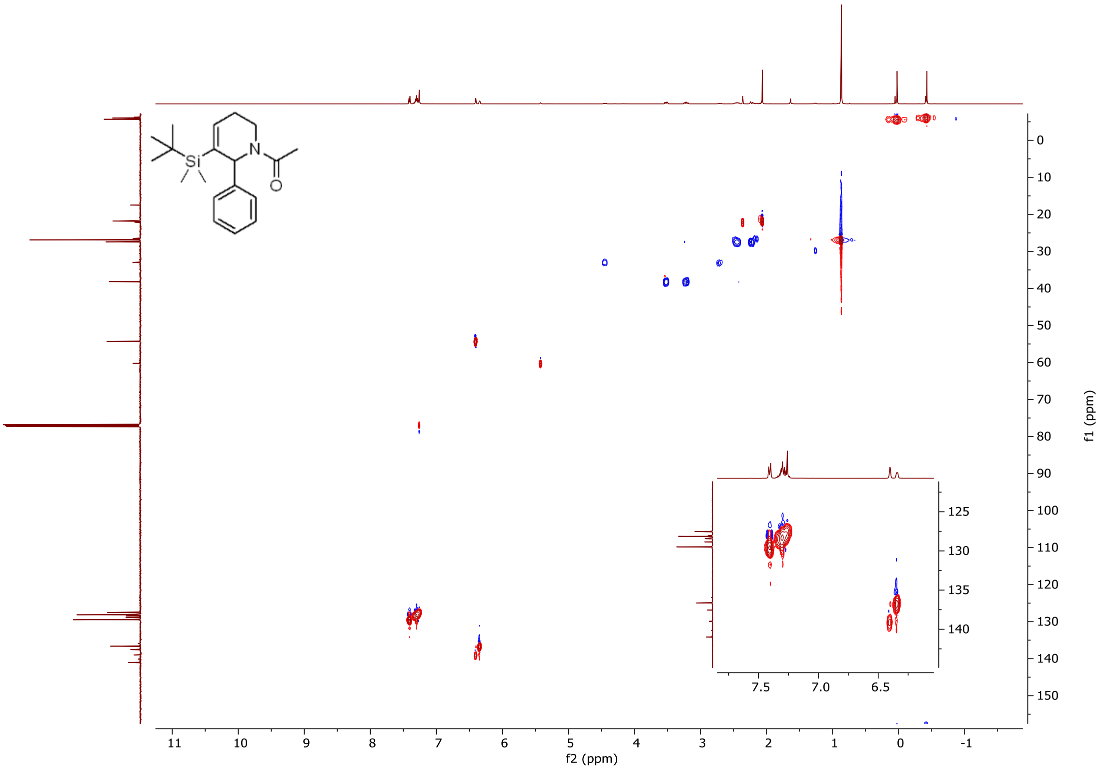

^{1}H-^{13}C-HMBC NMR ($CDCl_3$)

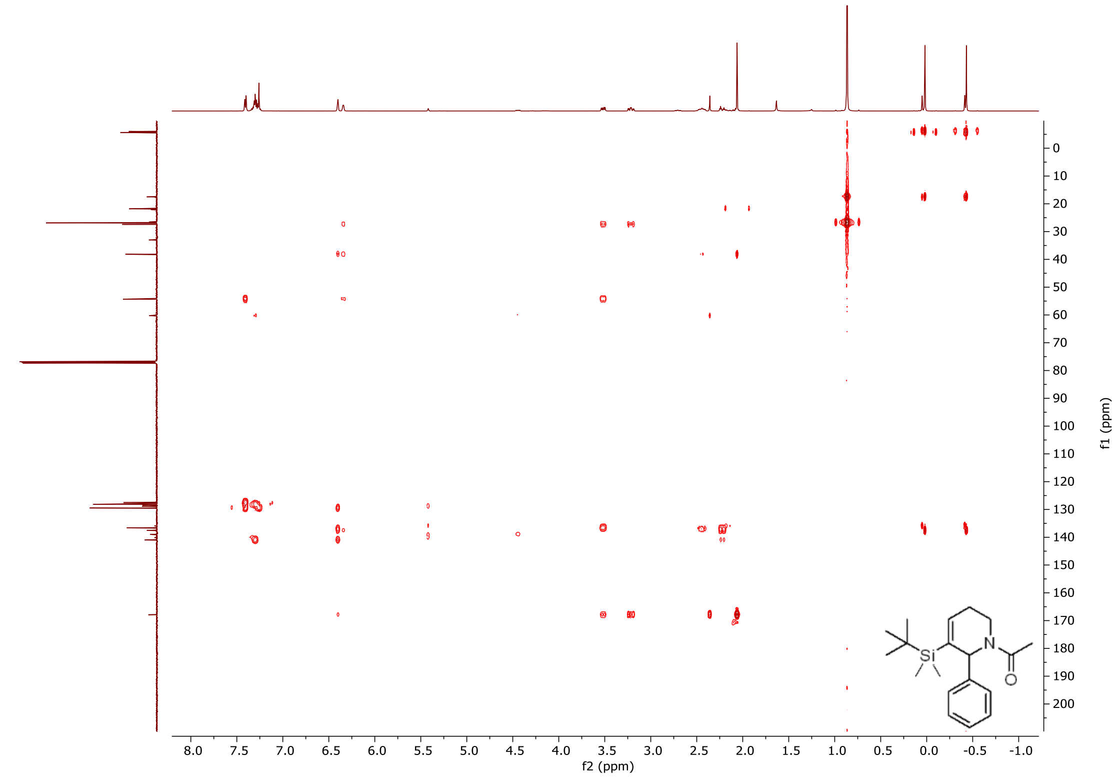

^{1}H-^{1}H-COSY NMR ($CDCl_3$)

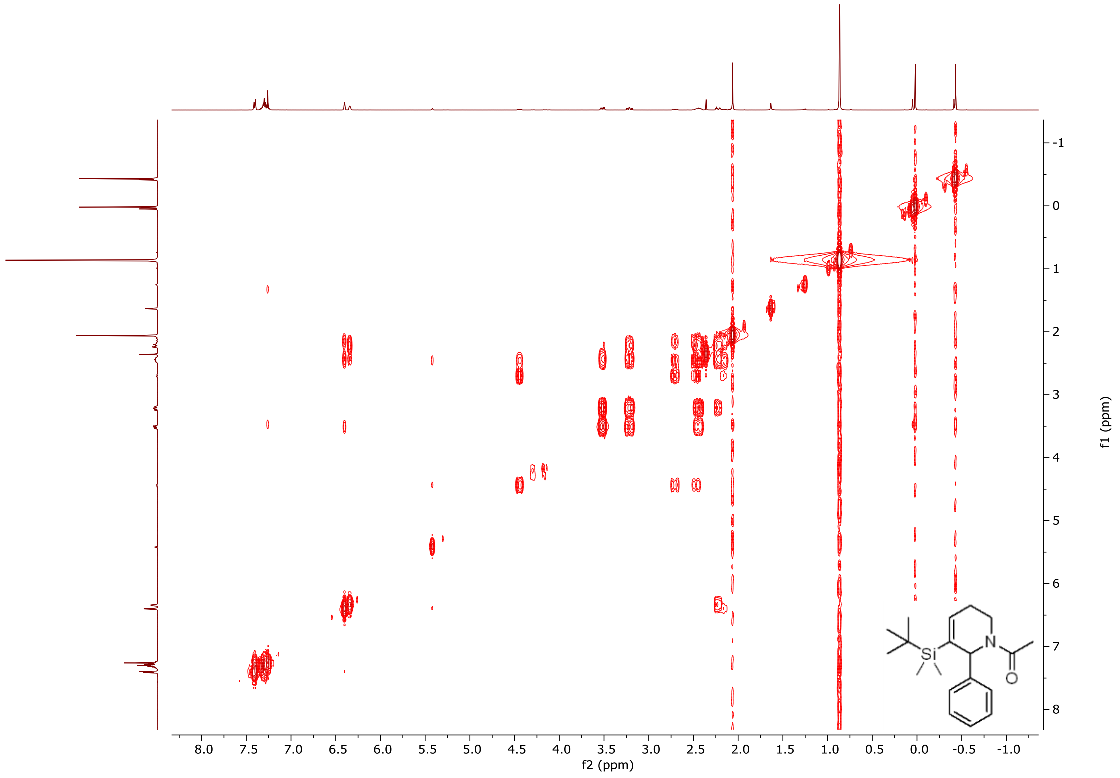

^{1}H-^{1}H-NOESY NMR ($CDCl_3$)

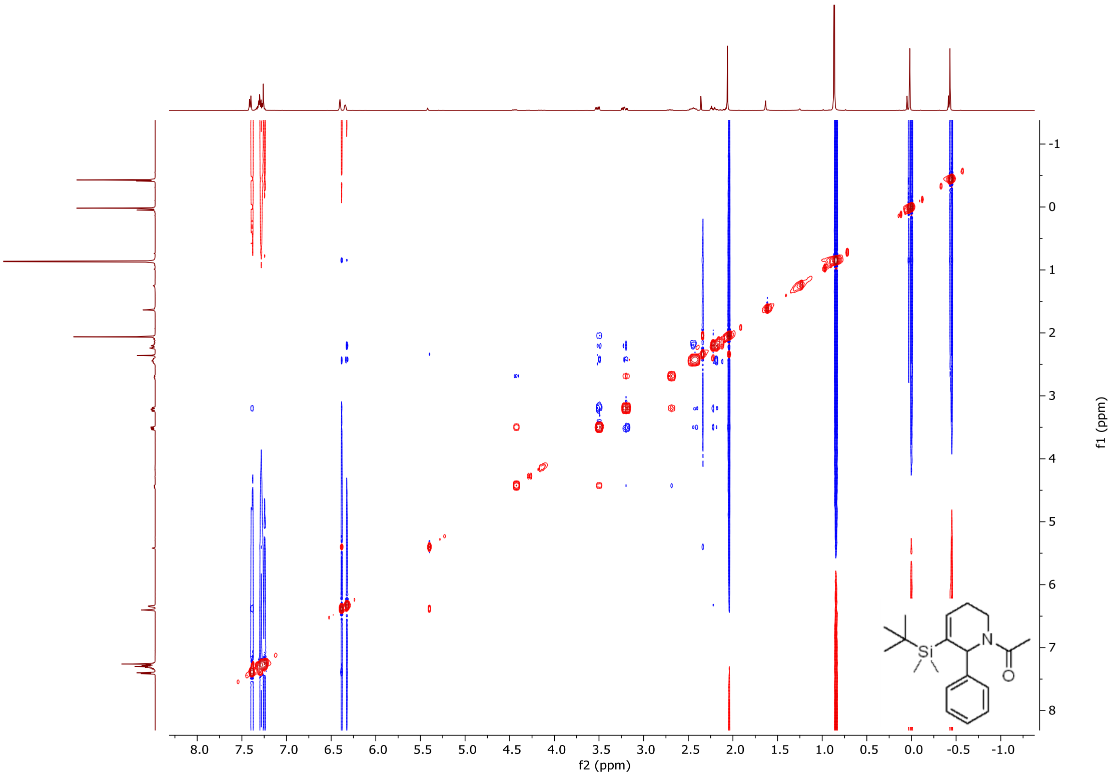

(5-(*tert*-Butyldimethylsilyl)-6-phenyl-3,6-dihydropyridin-1(2*H*)-yl)(phenyl)methanone (**9b**)

^{1}H NMR (500 MHz, $CDCl_3$):

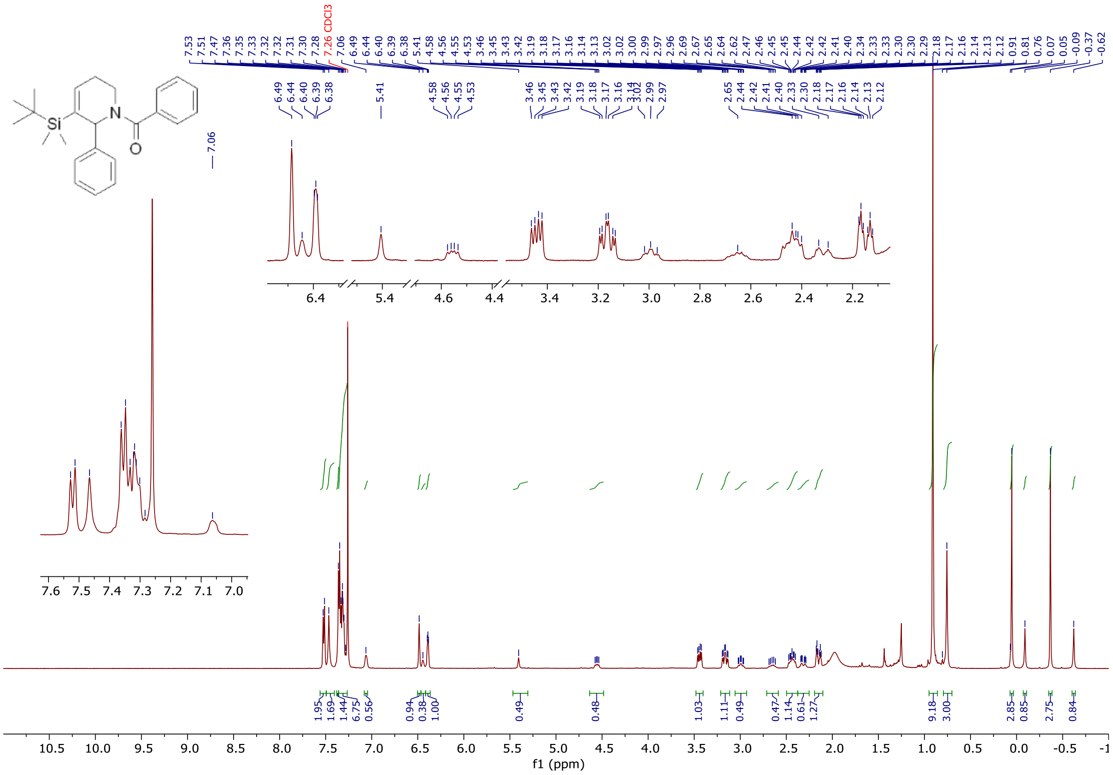

^{1}H NMR (500 MHz, DMSO-d_6, 80 °C)

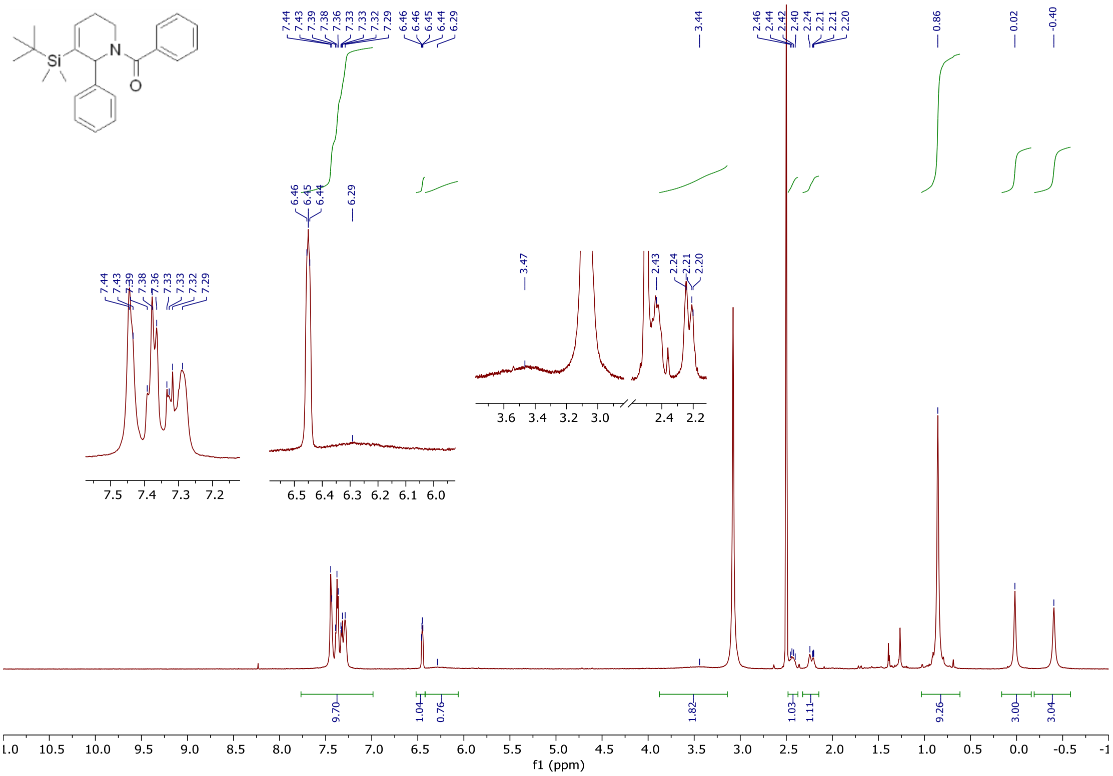

$^{13}C\ \{^{1}H\}$ NMR (126 MHz, DMSO-d_6, 25 °C)

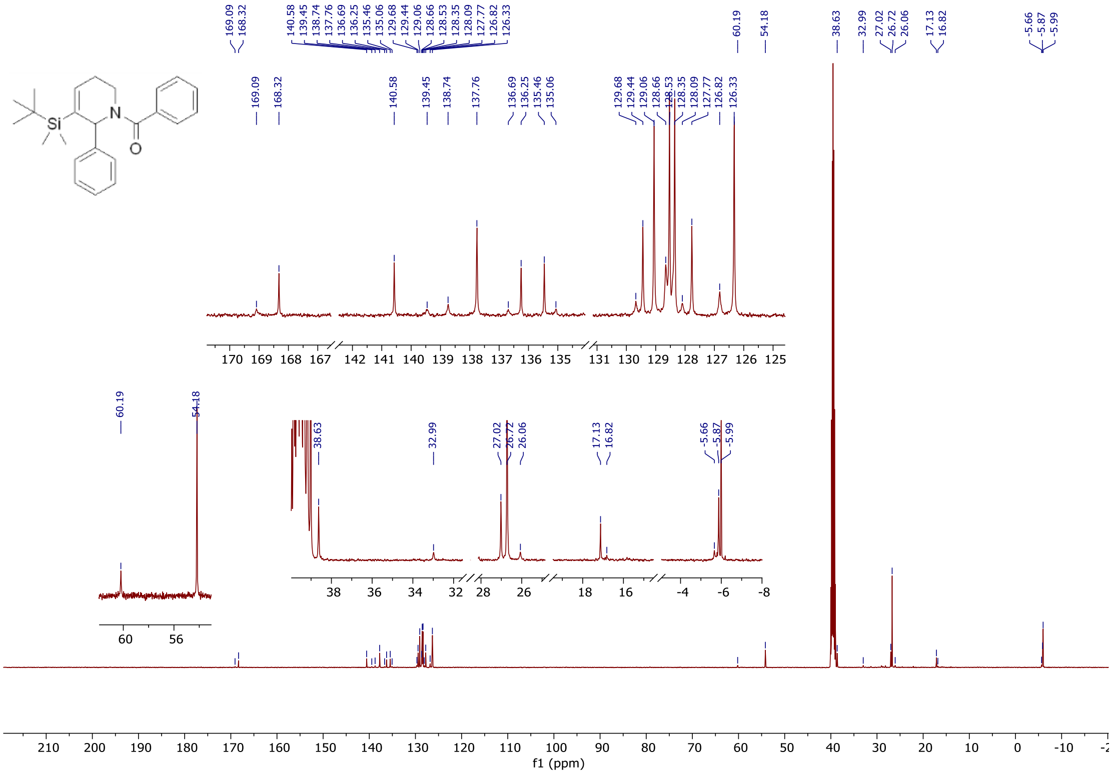

(5-(*tert*-Butyldimethylsilyl)-6-phenyl-3,6-dihydropyridin-1(2*H*)-yl)(3,5-dinitrophenyl)methanone (**9c**)

^{1}H NMR (500 MHz, CD_3CN, 25 °C)

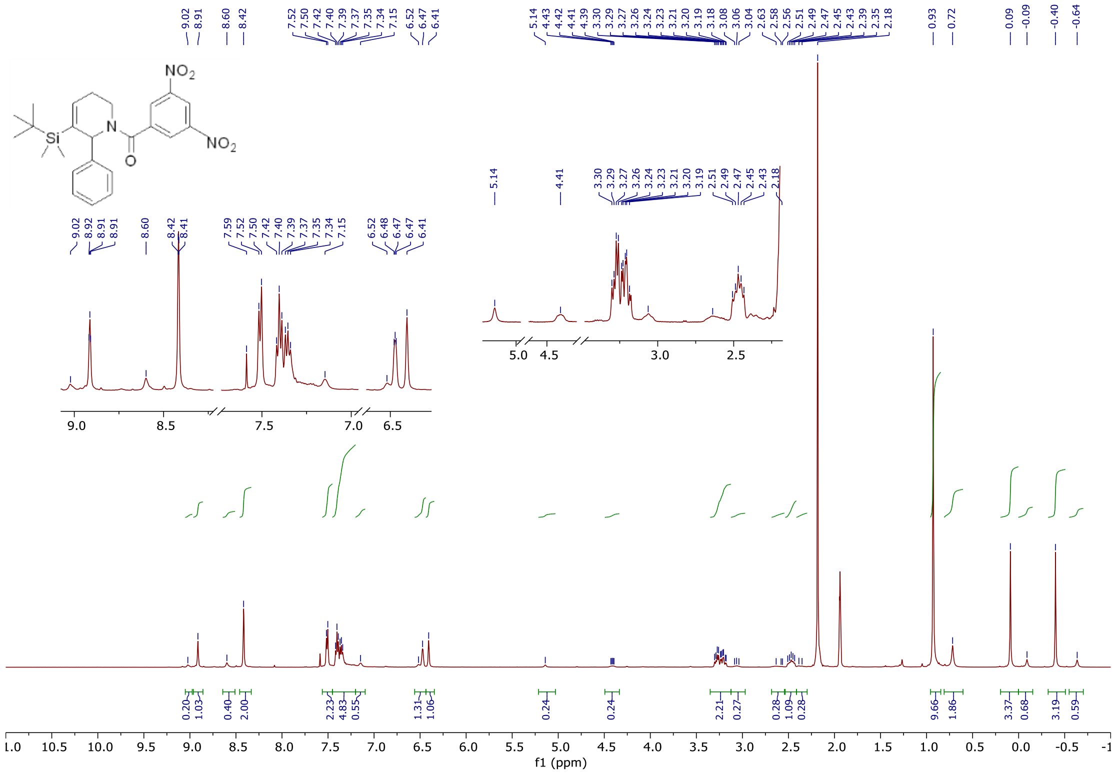

^{1}H-NMR (500 MHz, CD_3CN, 60 °C)

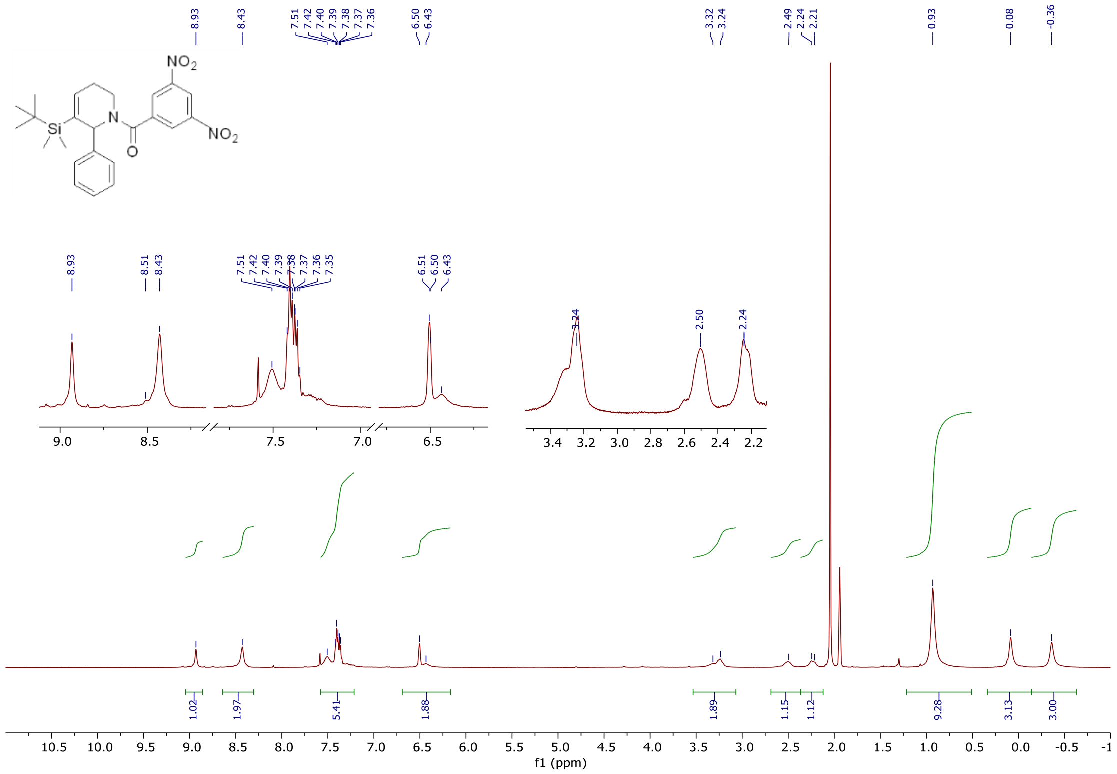

$^{13}C\ \{^{1}H\}$ NMR (126 MHz, CD_3CN, 25 °C)

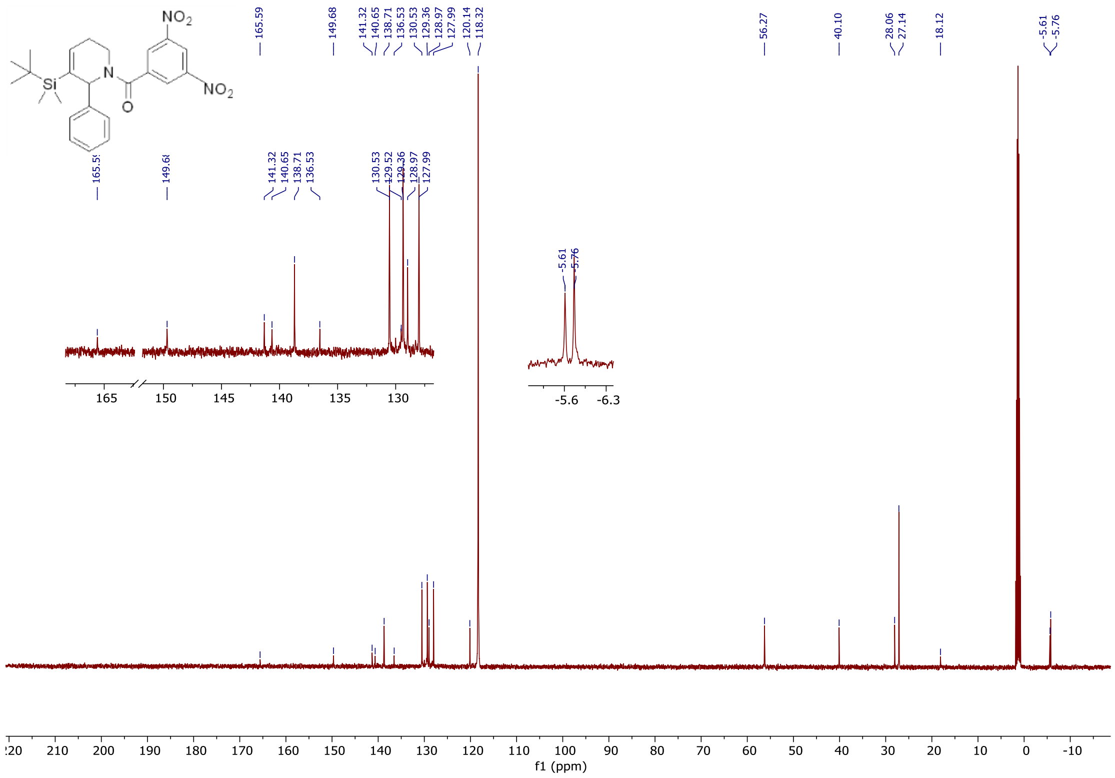

X-ray data

ORTEP diagram of compound 9c:

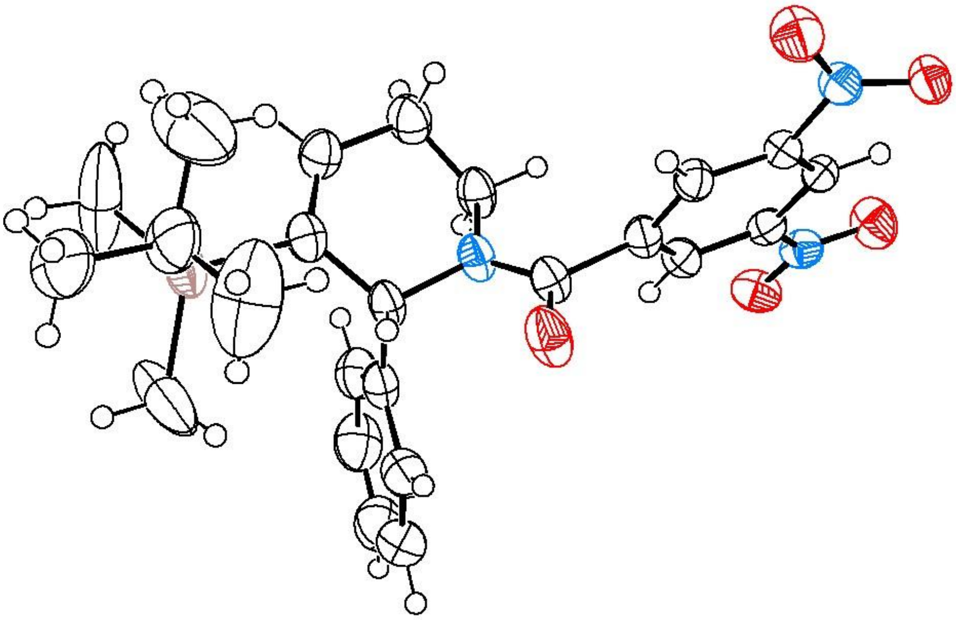

Figure S1. The molecular structure of the compound **9c**, showing 50 probability displacement ellipsoids. Crystallographic data have been deposited with the Cambridge Crystallographic Data Centre as a supplementary publication No. CCDC-2456515.

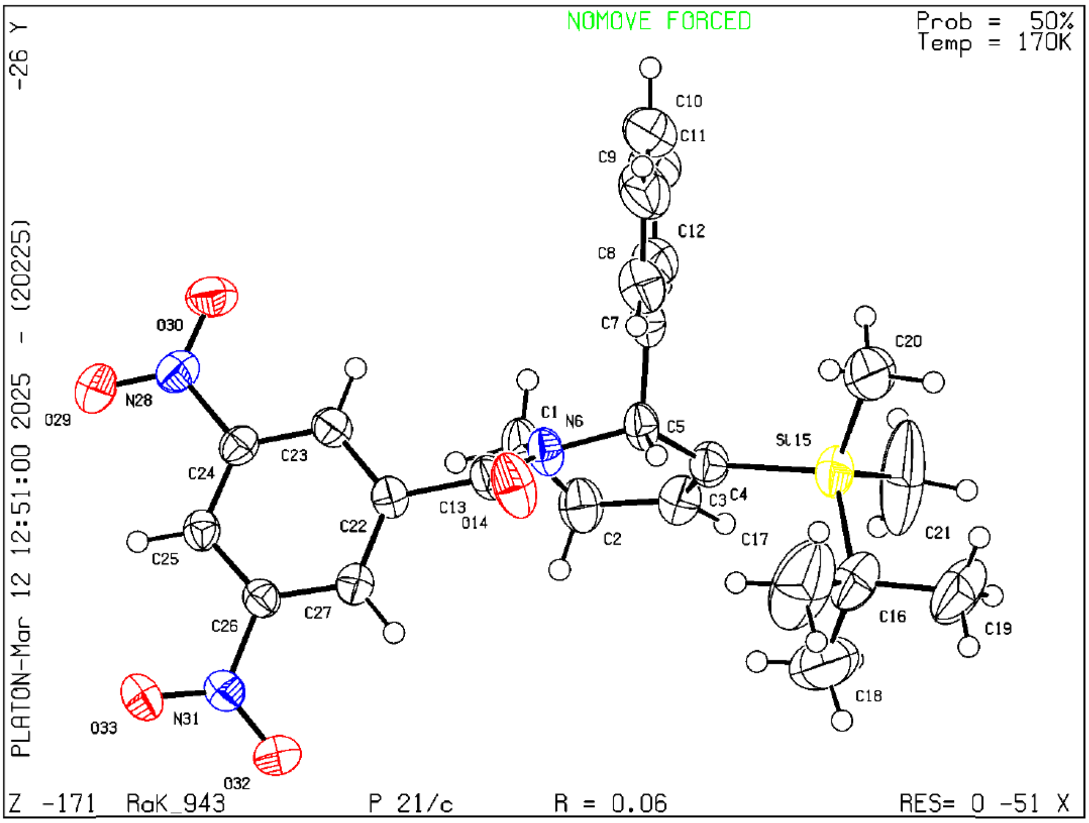

Figure S2. Ellipsoid plot for compound **9c**

Crystal parameters and refinement metrics of compound 9c

Crystallization conditions: 3 mg of the compound **9c** was dissolved in a mixture of EtOH (1.0 mL) and DCM (0.2 mL) and left to recrystallize to afford monocrystals suitable for X-ray crystallography analysis.

Table S4: Experimental details

Crystal data	
Chemical formula	$C_{24}H_{29}N_3O_5Si$
M_r	467.59
Crystal system, space group	Monoclinic, $P2_1/c$
Temperature (K)	170
a, b, c (Å)	20.6524 (3), 11.7506 (2), 10.1132 (2)
β (°)	91.639 (1)
V (Å^3)	2453.25 (7)
Z	4
Radiation type	Cu $K\alpha$
μ (mm^{-1})	1.17
Crystal size (mm)	0.2 × 0.08 × 0.05
Data collection	
Diffractometer	XtaLAB Synergy, Dualflex, HyPix
Absorption correction	Multi-scan<br>*CrysAlis PRO* 1.171.42.93a (Rigaku Oxford Diffraction, 2023) Empirical absorption correction using spherical harmonics, implemented in SCALE3 ABSPACK scaling algorithm.
T_{min}, T_{max}	0.685, 1.000
No. of measured, independent and observed [$I > 2\sigma(I)$] reflections	23606, 4960, 4305
R_{int}	0.043
$(\sin \theta/\lambda)_{max}$ (Å^{-1})	0.630
Refinement	
$R[F^2 > 2\sigma(F^2)]$, $wR(F^2)$, S	0.062, 0.178, 1.06
No. of reflections	4960
No. of parameters	303
H-atom treatment	H-atom parameters constrained
$\Delta\rho_{max}$, $\Delta\rho_{min}$ (e Å^{-3})	0.65, -0.56

References

(1) Wang, X.; Gao, Q.; Buevich, A. V; Yasuda, N.; Zhang, Y.; Yang, R.; Zhang, L.-K.; Martin, G. E.; Williamson, R. T. *J. Org. Chem.* **2019**, *84*, 10024–10031. doi:10.1021/acs.joc.9b01190

(2) Puriņš, M.; Mishnev, A.; Turks, M. *J. Org. Chem.* **2019**, *84*, 3595–3611. doi:10.1021/acs.joc.8b02735

(3) Kroņkalne, R.; Beļaunieks, R.; Ubaidullajevs, A.; Mishnev, A.; Turks, M. *J. Org. Chem.* **2023**, *88*, 13857–13870. doi:10.1021/acs.joc.3c01481

(4) Jiang, X.; Zhang, J.; Ma, S. *J. Am. Chem. Soc.* **2016**, *138*, 8344–8347. doi:10.1021/jacs.6b03948
